# Social marketing interventions for the prevention and control of neglected tropical diseases: A systematic review

**DOI:** 10.1371/journal.pntd.0008360

**Published:** 2020-06-17

**Authors:** Nathaly Aya Pastrana, Maria Lazo-Porras, J. Jaime Miranda, David Beran, L. Suzanne Suggs

**Affiliations:** 1 BeCHANGE Research Group, Institute for Public Communication, Università della Svizzera italiana, Lugano, Switzerland; 2 CRONICAS Centre of Excellence in Chronic Diseases, Universidad Peruana Cayetano Heredia, Lima, Peru; 3 Division of Tropical and Humanitarian Medicine, Geneva University Hospitals and University of Geneva, Geneva, Switzerland; 4 Department of Medicine, School of Medicine, Universidad Peruana Cayetano Heredia, Lima, Peru; 5 Swiss School of Public Health+, Zurich, Switzerland; Federal University of Agriculture Abeokuta, NIGERIA

## Abstract

**Background:**

Social marketing is an approach to behavior change that contributes to disease prevention and control. This study aimed to understand how social marketing interventions have addressed neglected tropical diseases (NTDs). It examined the characteristics, breadth of coverage, and outcomes of social marketing interventions focused on the prevention and control of these diseases.

**Methodology/Principal findings:**

Studies published in any language between January 1971 and April 2017, targeting at least one of the 17 NTDs prioritized in the World Health Organization (WHO) NTD Roadmap were considered. Included studies had interventions that applied both, at least one core social marketing concept, “social behavioral influence”, and one social marketing technique, “integrated intervention mix”, described in the Hierarchical Model of Social Marketing. This review is registered with PROSPERO CRD42017063858. Twenty interventions, addressing eight NTDs, met the inclusion criteria. They focused on behaviors related to four of the five WHO public health strategies for NTDs. Most interventions incorporated the concepts “relationship building” and “public / people orientation focus”, and the technique “insight-driven segmentation”. All the interventions reported changing behavioral determinants such as knowledge, 19 reported behavior change, and four influenced health outcomes.

**Conclusion/Significance:**

Evidence from this study shows that social marketing has been successfully used to address behaviors related to most of the five public health strategic interventions for NTDs recommended by the WHO. It is suggested that social marketing interventions for the prevention and control of NTDs be grounded on an understanding of the audience and adapted to the contexts intervened. Building stakeholder relationships as early as possible, and involving the publics could help in reaching NTD outcomes. Elements of the intervention mix should be integrated and mutually supportive. Incorporating health education and capacity building, as well as being culturally appropriate, is also relevant. It is recommended that ongoing discussions to formulate the targets and milestones of the new global Roadmap for NTDs integrate social marketing as an approach to overcome these diseases.

## Author summary

Neglected tropical diseases (NTDs) affect the life of more than a billion people, particularly those living in vulnerable conditions. Five priority strategies exist to prevent and control these diseases that require the adoption of preventive or control behaviors. Social marketing is an approach to behavior change that can help address NTDs. This study aimed to examine social marketing interventions focusing on NTDs using a systematic review of the literature. This qualitative study examined the characteristics, breadth of coverage, and outcomes of interventions for the prevention and control of NTDs. This was done using the Hierarchical Model of Social Marketing framework consisting of one principle, four concepts, and five techniques. The 20 Interventions retrieved were implemented in 13 countries, focused on eight diseases, and addressed four public health strategic interventions for NTDs. Most interventions incorporated the concepts “relationship building” and “public / people orientation focus”, and the technique “insight-driven segmentation”. Findings show that the interventions were successful in generating changes in behavioral determinants, behavior change, and health outcomes and that social marketing can be used to prevent and control NTDs. It is recommended that the new NTD Roadmap integrates this approach to behavior change.

## Introduction

The World Health Organization (WHO) prioritizes a group of neglected tropical diseases (NTDs) [1,2], which affect approximately one billion people around the world [3]. These are also known as “diseases of poverty” [4] or diseases of neglected populations [5]. These diseases affect the most vulnerable populations everywhere, not exclusively those living in lower-income economies [4,6]. NTDs pose a challenge for the health, social, and economic development of countries [5,7]. These challenges are exacerbated by changes in the global landscape, such as climate change, conflicts, migration, and urbanization processes [6,8–10].

Varied strategies exist to address NTDs. The WHO prioritizes five public health strategic interventions that include promoting behavior change [4,11]. The priority strategies are: 1) preventive chemotherapy and transmission control (PCT), 2) innovative and intensified disease management (IDM), 3) vector ecology and management (VEM), 4) veterinary public health measures, and 5) the provision of safe water, sanitation, and hygiene (WASH). Efforts to support behavior change to prevent and control NTDs are needed [4,12], and social marketing could contribute to such efforts.

“Social marketing seeks to develop and integrate marketing concepts with other approaches to influence behaviors that benefit individuals and communities for the greater social good. Social marketing practice is guided by ethical principles. It seeks to integrate research, best practice, theory, audience and partnership insight, to inform the delivery of competition sensitive and segmented social change programmes that are effective, efficient, equitable and sustainable.”

[13]

Social marketing, formally defined in 1971 [14], is a systematic approach to influence voluntary behaviors at multiple levels: downstream (individual), midstream (e.g. community), and upstream (e.g. policy) [15,16]. Social marketing interventions are characterized by adhering to a framework for planning, developing, implementing, and evaluating interventions. Many individual studies and systematic reviews illustrate the effectiveness of social marketing across a variety of health problems [17–20]. These reviews have used a variety of frameworks to describe social marketing interventions, some have focused on the traditional marketing mix, also known as the four or six Ps (product, price, place, promotion, policy, partnerships) [21,22], other have used the social marketing benchmarks [18,19,23–25]. The latter is the most widely used by social marketing scholars. This framework that was originally devised to distinguish social marketing from other approaches [26] such as social advertising, health education, and health communication [27,28], consists on a set of criteria, known as “the social marketing benchmarks”. Through the years, these benchmarks have been updated [29,30]. The most recent version includes eight criteria, namely: Behavior, citizen orientation, theory, insight, segmentation, exchange/value, methods mix, and competition [29]. To date, there is no consensus on which benchmarks are more relevant than others for planning, implementing, and evaluating social marketing interventions [15].

More recently, French and Russell-Bennet proposed a new framework to categorize the social marketing benchmark criteria in a hierarchy of importance [15]. The *Hierarchical Model of Social Marketing* [15] proposes that characteristics of social marketing can be grouped in three clusters or categories and that a hierarchy exists among these. The first category is *principle* and refers to social value creation through exchange, a characteristic exclusive of social marketing. This is generated through the second category, *concepts*, grouping four concepts that are consistent with the definition of social marketing and that are essential to creating social value. The four concepts are: (1) social behavioral influence, (2) citizen/customer/civic society orientation focus, (3) social offerings, and (4) relationship building. According to the authors of the framework, the uniqueness of social marketing lies in the interaction of the principle and the four concepts [15]. The third category is *techniques* and refers to methods and tactics that are commonly, but not exclusively, found in social marketing interventions. The five techniques listed in the model are: (1) integrated intervention mix, which includes the Ps of the marketing mix, (2) competition analysis and action, (3) systematic planning and evaluation, (4) insight-driven segmentation, and (5) co-creation through social markets.

This qualitative study intends to fill a scientific gap by carrying out a systematic review examining the characteristics and breadth of coverage of social marketing interventions studied for the prevention and control of NTDs. The study aims to understand how social marketing interventions have been used to address NTDs. The Hierarchical Model of Social Marketing is used to identify the concepts and techniques used. Differences in the use of these elements of the Hierarchical Model are highlighted when present. The behavioral determinants, behavior changes, and health outcomes addressed by the interventions are summarized according to the five WHO public health strategic interventions for NTDs. The overall success of the interventions in attaining the desired outcomes is also presented as reported by the interventions.

## Methods

### Study protocol

This systematic review was registered with PROSPERO (CRD42017063858) [31]. The protocol, published elsewhere [32], followed the guidelines of the Preferred Reporting Items for Systematic Review and Meta-Analysis Protocols (PRISMA-P) [33].

### Eligibility criteria

Peer-reviewed primary studies published between January 1971, the year the social marketing concept was coined [14], and April 2017, in any language, were included if they targeted the prevention or control of any of the 17 NTDs prioritized in the WHO NTD Roadmap [34]. No restrictions were made in the countries to include because NTDs affect the most vulnerable in low income and wealthier economies. No limits were set on the diseases as the study intended to identify the variety of priority diseases addressed by social marketing interventions.

Studies were retrieved if the interventions were labeled as social marketing or used social marketing terminology in their title or abstract, such as “marketing mix”, “segmentation”, “exchange”, “social offering”, and “behavior influence”. Studies that applied at least one concept, “social behavioral influence”, and one technique, “integrated intervention mix” were included in the review. These two criteria were established because they are common in social marketing interventions [17,24,35–38], and because interventions dated before 2015, the year the Hierarchical Model of Social Marketing was published, might explicitly state that they adhered to these criteria. Moreover, to be included, the interventions had to report their intended or actual influence on behavioral determinants (e.g., attitudes, awareness, knowledge, infrastructure or policy change), behavior change, or health outcomes. Reviews and systematic reviews were excluded.

### Information sources and search strategies

A literature search was conducted in PubMed, EbscoHost, ProQuest, Web of Science, Global Index Medicus, and Virtual Health Library Regional Portal. The search strategies are available in the protocol [32], at PROSPERO (CRD42017063858) [31], and in the supporting information (see S1 File). Reference lists of the selected studies were searched to identify additional relevant articles. When full-text articles were not available, corresponding authors were contacted. Some authors provided further publications describing the interventions and papers about interventions that had not been found through the literature search.

### Study selection

Records obtained through database searches were imported to Covidence [39]. Titles and abstracts were screened for eligibility criteria. The full text of each article was screened independently by two reviewers to assess eligibility. A third reviewer helped reach consensus when there were discrepancies.

### Data extraction

Data extraction, coding, and analysis processes were conducted in Microsoft Excel by one reviewer. The unit of analysis was the social marketing intervention, and multiple publications reporting the same social marketing intervention were grouped together. Some authors were also contacted for clarifications or to confirm data.

### Modifications to the Hierarchical Model of Social Marketing

The Hierarchical Model of Social Marketing was used as a framework for analysis for several reasons. It is built on other social marketing frameworks, especially on the social marketing benchmark criteria; it is the most recent framework to describe components of social marketing interventions; and it uncovers elements of social marketing interventions that are not explicitly mentioned by other frameworks (e.g. relationship building, co-creation). Modifications were made to the labels of the items in the framework, as well as to some descriptions. The name of the concept “citizen/customer/civic society orientation focus” was changed to “public/people orientation focus”. Part of the description of the technique “co-creation” was removed because it was repeated in the concept “relationship building. Some other texts were paraphrased. The adapted model is presented in Table 1.

**Table 1:** The Hierarchical Model of Social Marketing: Principle, concepts and techniques.

**Social Marketing Principle**				
Aim: To bring social value through reciprocal exchange of resources or assets at the individual, community, societal or global level. Social policy, strategy, products, services, and/or experiences are developed that will enable or assists publics to derive social benefits individually or collectively.				
**Core Social Marketing Concepts**				
*1—Social behavioral influence* Behavioral analysis is undertaken. Interventions seek to influence specific behaviors or clusters of related behaviors. Specific actionable and measurable objectives and indicators are established. Behavioral theory is used. Behaviors could be upstream, midstream or downstream.	*2 –Public / people orientation focus* Planning, delivery and evaluation are focused on building understanding and interventions around people’s beliefs, attitudes, behaviors, needs and wants. Different research analyses combining qualitative and quantitative data gathering are used and synthesized to plan, deliver and review interventions.	*3—Social offerings* Publics (citizens, policy-makers or stakeholders) are offered products, ideas, understanding, services, experiences, systems and environments that provide value and advantage. Social offerings are in most cases positive in nature, although some can involve the imposition of restrictions that have collective support and benefit.	*4—Relationship building* Publics are engaged in the selection of priorities, and the development, design, implementation and evaluation of interventions.	
**Social Marketing Techniques**				
*1—Integrated intervention mix* Strategies for change that include the marketing mix and other strategies. Driven by target market insight data, segmentation analysis, competition analysis and feasibility analysis. Mix of “types” and “forms” of interventions that are selected and coordinated to produce an effective and efficient program.	*2—Competition analysis and action* Internal (e.g. internal psychological factors, pleasure, desire, risk taking, genetics, and addiction, etc.) and external competition is assessed (e.g. economic, social, cultural and environmental influences). Strategies are developed to reduce the impact of negative competition on the target behavior.	*3—Systematic planning and evaluation* Interventions use proven strategy and planning theory and models to construct robust intervention plans that include formative research, pretesting, situational analysis, monitoring, evaluation, and the implementation of learning strategies.	*4—Insight-driven segmentation* Segmentation using demographic, observational data and psycho-graphic data is used to identify groups that are similar and can be influenced in common ways. Segmentation leads to the development of an intervention mix directly tailored to specific target market needs, values and circumstances.	*5—Co-creation* Strategies are developed to maximize the contribution of partner and stakeholder coalitions in achieving targeted behaviors.

Adapted from: French and Russell-Bennet [15]

### Data items

Extracted information included: author(s), paper title, location, world region classified according to the six WHO regional groupings, country income category as classified by the World Bank, the aim of the intervention, NTD, population targeted, social marketing concepts, and social marketing techniques. Changes in behavioral determinants, behavior change, and health outcomes were also extracted. The behavior focus was categorized according to the five WHO public health strategic interventions for NTDs.

### Quality assessment

The quality of included studies was assessed in the final stage of the review. The assessment was conducted using the QATSDD Critical Appraisal Tool [40] that assesses “the congruency, transparency and organized reporting of the research processes” [41] of studies of diverse designs (qualitative, quantitative, mixed). This tool was selected because it can be used to assess both quantitative and qualitative studies, and social marketing studies commonly use both. The tool, used in other systematic reviews [42,43], comprises 16 criteria, each having a scoring scale from 0 to 3 (0 = not at all, 1 = very slightly, 2 = moderately, 3 = complete). Following the scoring guidelines [40] two reviewers, who did not participate in the screening or data extraction processes, assigned a score to the studies describing intervention results, calculated overall scores per intervention, and then consolidated results. To compare the quality across studies and following the QATSDD guidelines [40], the total scores of the interventions were converted to percentages. All studies were included independently of the appraisal results. This to have an overall understanding of the concepts and techniques incorporated by the interventions, and not limit findings only to studies of high-quality scores. Nevertheless, the results and implications of the quality assessment are presented in the following sections.

### Synthesis of results

Extracted data were synthesized according to the five social marketing concepts, the four social marketing techniques, behavioral determinants, behavior changes, and health outcomes, and the five WHO public health strategic interventions for NTDs. Comparisons and meta-analysis were not planned nor conducted in this review.

## Results

### Study selection

**Fig 1:**
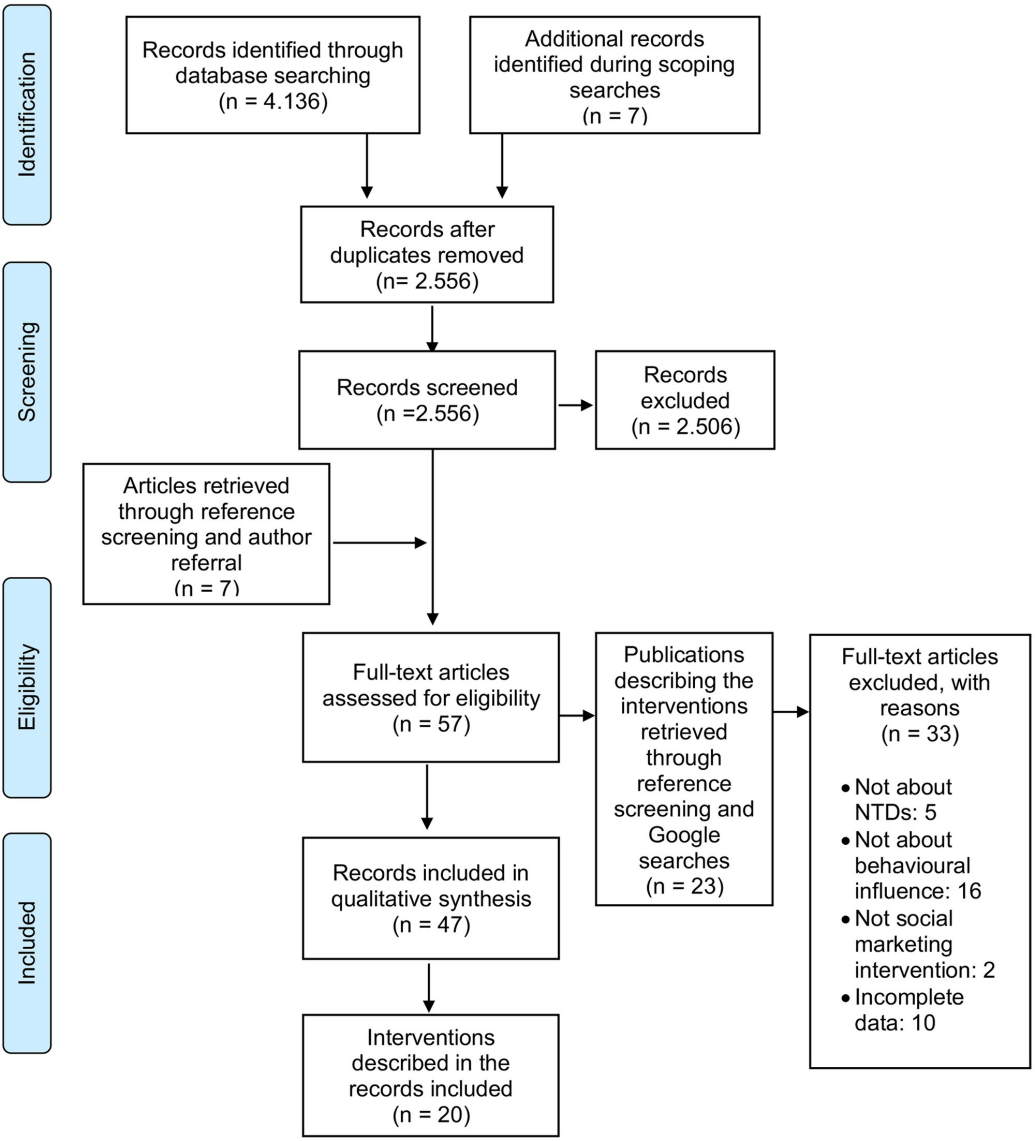
PRISMA 2009 flow diagram. Study selection process [46].

Out of 2,556 records screened, 57 full-text articles were retrieved to confirm eligibility. Additional publications (n = 23) complementing the interventions described in those 57 articles were retrieved through reference screening and Google searches. This led to the inclusion of 47 publications describing the 20 interventions that were included in the analysis. Most were published in English, with the exception of one in Spanish [44] (see Fig 1). As specified in the research protocol, a separate analysis of the gender responsiveness of the interventions included in this systematic review is reported elsewhere [45].

**Fig 2:**
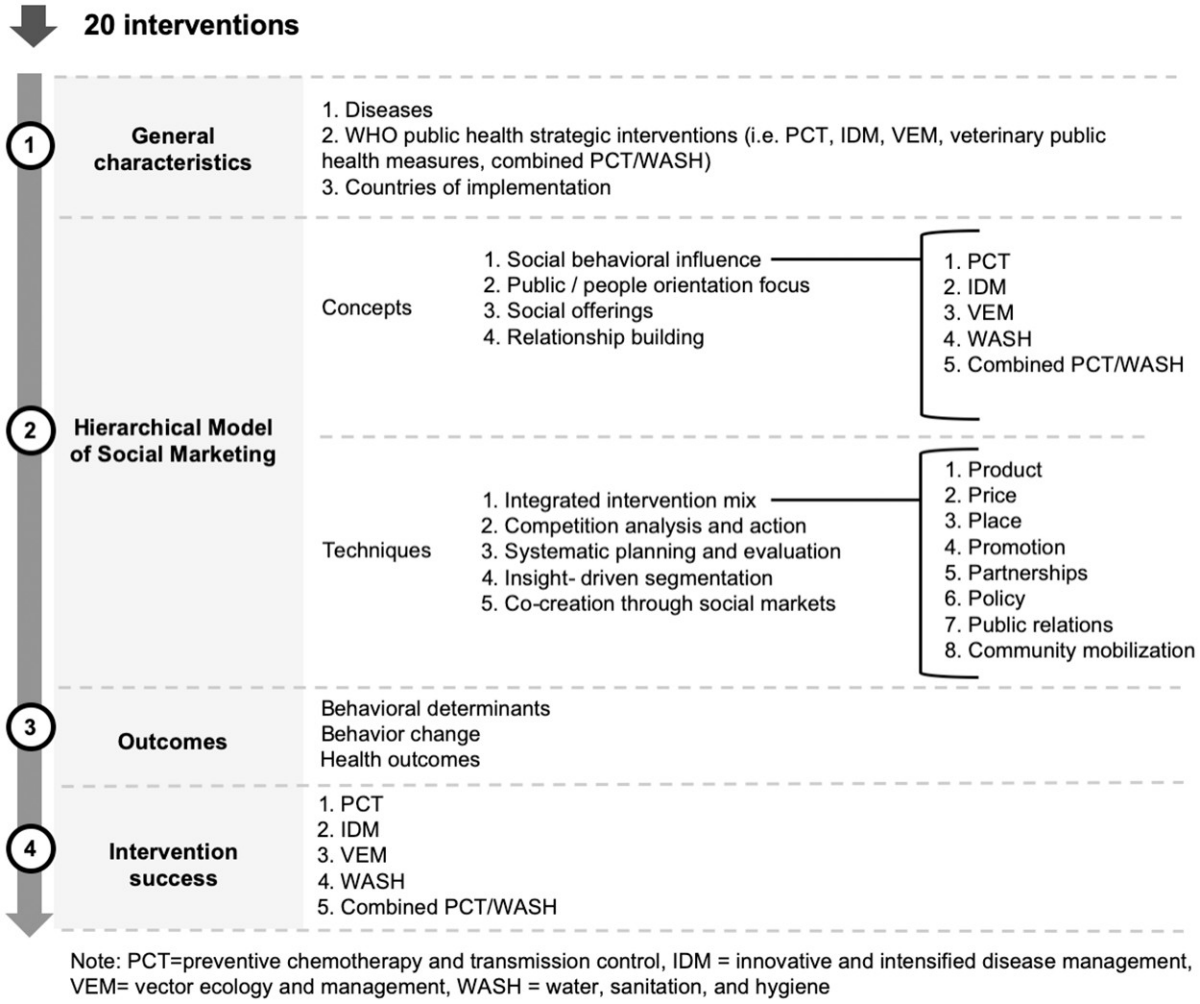
Presentation of the results section.

The results section is organized in four parts (see Fig 2). First, the characteristics of the interventions in terms of diseases of focus, the five WHO public health strategic interventions for NTDs they related to, and the countries of implementation are presented. Then, following the elements of the Hierarchical Model of Social Marketing, the concepts and techniques used by the interventions are highlighted. This is followed by a section focused on the outcomes reported by the interventions in terms of behavioral determinants, behavior change, and health outcomes. Finally, the success in reaching the outcomes is showcased. These last two sections are presented according to the five WHO public health strategic interventions for NTDs.

### General characteristics of the interventions

Interventions addressed eight NTDs: cysticercosis (n = 1), dengue (n = 7), Guinea worm disease (n = 2), leprosy (n = 1), lymphatic filariasis (n = 3), schistosomiasis (n = 4), soil-transmitted helminths (n = 1), and trachoma (n = 1). The interventions focused on four of the five WHO public health strategic interventions for NTDs, namely: PCT (n = 4), IDM (n = 1), VEM (n = 7), and WASH (n = 6). Two interventions used combined PCT and WASH strategies, and none focused on veterinary public health measures.

According to the WHO regional classification, the interventions were implemented in Africa (n = 3), the Americas (n = 5), the Eastern Mediterranean (n = 1), South-East Asia (n = 4), and the Western Pacific (n = 7). More precisely in the following countries: Australia (n = 1), Brazil (n = 1), China (n = 5), Colombia (n = 1), Honduras (n = 2), India (n = 1), Indonesia (n = 1), Mexico (n = 1), Nigeria (n = 2), Saudi Arabia (n = 1), Sri Lanka (n = 2), Tanzania (n = 1), and America Samoa a territory of the United States (n = 1).

Five of the seven dengue interventions were conducted in Latin America, three of the four schistosomiasis interventions were implemented in China, and five of the seven interventions carried out in the Western Pacific region focused on WASH. See Table 2 for a list of the interventions.

**Table 2:** Included interventions.

WHO Priority Strategy	NTD	Author, year [reference]	Location, WHO region	Country Income level	Public	Aim
Preventive chemotherapy and transmission control (PCT)	Lymphatic filariasis	Ramaiah et al., 2006 [50]	Tamil Nadu State—India, SEA	LMIC	General population, State, district and village level administrations	Motivate the population to participate and take treatment (DEC, DEC-albendazole) offered via MDA on Filaria Day.
		King et al., 2011 [48]	American Samoa—United States territory, WP	UMIC	General population	Increase coverage of the annual MDA with albendazole and DEC.
		Krentel et al., 2006 [49]	Alor District -Indonesia, SEA	LMIC	Communities	Motivate the population to take the required treatment for filariasis that was offered during MDA for prevention and control.
	Schistosomiasis (SCH)	Yuan et al., 2005 [53]	Junshan district—Hunan Province—China, WP	UMIC	Schoolchildren	Increase children’s knowledge and adherence to screening and chemotherapy treatment.
Innovative and intensified disease management (IDM)	Leprosy	Salgado, 1993 [51]; Williams et al., 1998 [52]; Wong, 2002 [117]; Brown, 2006 [91]	Sri Lanka, SEA	LMIC	Individuals with suspicious lesions on the skin, health care providers, and the general public	Prompt individuals with suspicious lesions on the skin to seek treatment by self-referral; have health care providers to identify and refer leprosy cases for treatment; and reduce fear to leprosy among the general population.
Vector ecology and management (VEM)	Dengue	Caprara et al., 2015 [57]; Alfonso-Sierra et al., 2016 [56]	Fortaleza—Brazil, AM	UMIC	Communities	Reduce *Aedes aegypti* vector density by controlling productive container types and discarded containers through an ecohealth approach.
		Escudero-Támara and Villareal-Amaris, 2015 [44]	Sincelejo—Colombia, AM	UMIC	Family members of schoolchildren	Prompt behavior change to eliminate breeding places of dengue vector.
		Abeyewickreme et al., 2012 [55]; Arunachalam et al., 2010 [90]	Gampaha District Sri Lanka, SEA	LMIC	Communities	Waste management to reduce vector breeding places at the household level.
		NK Ibrahim et al., 2009 [59–61]	Jeddah—Saudi Arabia, EM	HIC	Female high-school students, teachers and supervisors	Improve knowledge, attitudes and practices related to dengue fever.
		Lloyd et al., 1992 [64]; Winch et al., 1991 [66]; Lloyd et al., 1994 [65]; Kendall et al., 1991 [62]	Merida—Yucatan—Mexico, AM	LMIC	Communities	Eliminate or control larval production sites at the household.
		Leontsini et al., 1993 [63]; Kendall et al., 1991 [62]	El Progreso—Honduras, AM	LMIC	Communities	Reduce Ae. aegypti larval infestation indices by promoting the control of four types of containers (i.e. tyres, pilas, can, drums).
		Fernández et al., 1998 [58]	El Progreso–Honduras, AM	LMIC	Community householders	Promote the use of the five-steps cleaning method "La Untadita" to reduce mosquito infestation in cement washbasins and metal drums.
Water, sanitation and hygiene (WASH)	Cysticercosis (CYS)	Dickey et al., 2015 [77]; Dickey et al., 2016 [78]; Dickey, 2014 [76]	Eryuan County—Yunnan—China, WP	UMIC	Communities of Bai people (minority ethnic group)	Increase household toilet building and use for CYS reduction.
	Guinea-worm disease (GUI)	Brieger et al., 1989 [74]; Brieger et al., 1986 [73]; Brieger et al., 1990 [75]; Adeniyi and Brieger, 1983 [67]	Idere—Nigeria, AF	LMIC	Communities	Prompt the communities to buy and use a monofilament nylon cloth filter to prevent GUI.
		Adeyanju, 1987 [68]	Lagon and Ogun States—Nigeria, AF	LIC	Community members	Increase knowledge and promote the adoption of preventive measures against GUI.
	Schistosomiasis (SCH)	Yuan et al., 2000 [89]	Donting Lakes region—China, WP	UMIC	Schoolchildren	Reduce contact with infested water by discouraging children from playing with it.
	Soil-transmitted helminths	Bieri, Yuan, et al., 2013 [72]; Bieri, Gray, et al., 2013 [71]	Linxiang City District—Hunan province—China, WP	UMIC	Schoolchildren	Increase knowledge about soil-transmitted helminths, promote behavior change and reduce the rate of infection.
	Trachoma	Atkinson et al., 2014 [69]; Lange et al., 2014 [85]; Lange et al., 2017 [86]; Baunach et al., 2012 [70]; Stanford et al., 2016 [87]; Lange et al., 2016 [84]; Taylor et al., 2012 [88]; Lange et al., 2012 [82]; Lange et al., 2015 [83]; Jones et al., 2015 [79]; Lange, JR Atkinson, et al., 2013 [80]; Lange, J Atkinson, et al., 2013 [81]	Northern Territory (NT)—Australia, WP	HIC	Health, education and community support settings staff, children and their carers	Improve the knowledge, attitudes and practices of health, education and community support settings staff and their ability to teach others about trachoma prevention; and improve hand and facial hygiene practices among children and carers.
Combined PCT and WASH	Schistosomiasis (SCH)	Freudenthal et al., 2006 [54]	Kileo and Kivulini–Tanzania, AF	LIC	Schoolchildren	Create enabling environments for communities to adopt practices to reduce SCH transmission.
		Hu et al., 2005 [47]	Poyang Lake area—China, WP	UMIC	Schoolchildren, adult women, adult men	Increase awareness and knowledge about SCH, reduce the frequency of infested water contact and increase compliance with praziquantel-based chemotherapy.

Note: DEC = diethylcarbamazine, MDA = Mass Drug Administration, Africa = AF, Americas = AM, Eastern Mediterranean = EM, South-East Asia = SEA, Western Pacific = WP, low-income economies = LIC, lower-middle-income economies = LMIC, upper-middle-income economies = UMIC, high-income economies = HIC

### Social marketing concepts and techniques used

The Hierarchical Model of Social Marketing concepts are: (1) social behavioral influence, (2) public / people orientation focus, (3) social offerings, and (4) relationship building. The five techniques listed in the model are: (1) integrated intervention mix, (2) competition analysis and action, (3) systematic planning and evaluation, (4) insight-driven segmentation, and (5) co-creation through social markets. See Table 3 for an overall summary and the supporting information (S2 File) for more details of the elements from the hierarchical model of social marketing included in the interventions.

**Table 3:** Hierarchical model of social marketing incorporated by the interventions.

Author, year [reference]	Core Social Marketing Concepts				Social Marketing Techniques				
	Social Behavioral Influence	Public Orientation Focus	Social Offerings	Relationship Building	Integrated Intervention Mix	Competition Analysis and Action	Systematic Planning and Evaluation	Insight-driven Segmentation	Co-Creation
Brieger et al., 1989 [74]; Brieger et al., 1986 [73]; Brieger et al., 1990 [75]; Adeniyi and Brieger, 1983 [67]	✓	✓	✓	✓	✓	✓	✓ (p, e)	✓	✓
Dickey et al., 2015 [77]; Dickey et al., 2016 [78]; Dickey, 2014 [76]	✓	✓	✓	✓	✓	✕	✓ (p, e)	✓	✓
Caprara et al., 2015 [57]; Alfonso-Sierra et al., 2016 [56]	✓	✓	✓	✓	✓	✕	✕ (e)	✕	✓
Ramaiah et al., 2006 [50]	✓	✓	✓	✓	✓	✕	✓ (p, e)	✓	✕
Atkinson et al., 2014 [69]; Lange et al., 2014 [85]; Lange et al., 2017 [86]; Baunach et al., 2012 [70]; Stanford et al., 2016 [87]; Lange et al., 2016 [84]; Taylor et al., 2012 [88]; Lange et al., 2012 [82]; Lange et al., 2015 [83]; Jones et al., 2015 [79]; Lange, JR Atkinson, et al., 2013 [80]; Lange, J Atkinson, et al., 2013 [81]	✓	✓	✓	✓	✓	✓	✓ (p, e)	✓	✓
King et al., 2011 [48]	✓	✓	✓	✓	✓	✕	✕ (e)	✓	✕
Krentel et al., 2006 [49]	✓	✓	✓	✓	✓	✓	✕ (e)	✓	✓
Salgado, 1993 [51]; Williams et al., 1998 [52]; Wong, 2002 [117]; Brown, 2006 [91]	✓	✓	✓	✓	✓	✓	✓ (p, e)	✓	✕
Freudenthal et al., 2006 [54]	✓	✓	✓	✓	✓	✕	✕ (p)	✓	✓
Bieri, Yuan, et al., 2013 [72]; Bieri, Gray, et al., 2013 [71]	✓	✓	✓	✓	✓	✕	✓ (p, e)	✓	✓
Escudero-Támara and Villareal-Amaris, 2015 [44]	✓	✓	✕	✓	✓	✕	✓ (p, e)	✓	✕
Abeyewickreme et al., 2012 [55]; Arunachalam et al., 2010 [90]	✓	✓	✓	✓	✓	✕	✓ (p, e)	✓	✓
NK Ibrahim et al., 2009 [59–61]	✓	✓	✕	✕	✓	✕	✓ (p, e)	✕	✕
Hu et al., 2005 [47]	✓	✓	✓	✓	✓	✕	✓ (p, e)	✓	✕
Adeyanju, 1987 [68]	✓	✓	✓	✓	✓	✓	✓ (p, e)	✓	✓
Lloyd et al., 1992 [64]; Winch et al., 1991 [66]; Lloyd et al., 1994 [65]; Kendall et al., 1991 [62]	✓	✓	✕	✓	✓	✓	✓ (p, e)	✓	✓
Leontsini et al., 1993 [63]; Kendall et al., 1991 [62]	✓	✓	✕	✓	✓	✕	✕ (e)	✕	✓
Yuan et al., 2005 [53]	✓	✓	✕	✓	✓	✕	✕ (e)	✓	✓
Fernández et al., 1998 [58]	✓	✓	✓	✓	✓	✕	✓ (p, e)	✓	✕
Yuan et al., 2000 [89]	✓	✓	✕	✕	✓	✕	✕ (e)	✓	✕
Total	20	20	14	18	20	6	13	17	12

Note: evidence of planning only (p); evidence of evaluation only (e); evidence of planning and evaluation (p, e)

#### Concept 1: Social behavioral influence

This concept refers to behaviors or clusters of behaviors the interventions intended to influence, and the use of behavioral theories, analyses, and models to understand them [15]. All the interventions integrated characteristics of this concept (see Table 3, S2 File). The behaviors of focus of the interventions are consistent with four of the WHO public health strategic interventions for NTDs. Interventions addressing behaviors related to PCT focused mostly on influencing the target audiences to take treatment [47–53] as well as on general preventive measures [54], and increasing screening [53]. The intervention related to IDM focused on individuals with suspicious skin lesions to seek diagnosis and comply with multidrug therapy (MDT) for leprosy [51,52]. Prevention and control interventions related to VEM, focused on the control or elimination of mosquito breeding places [44,55–66]. WASH behaviors focused on general preventive measures [47,54,67–89], and more specific behaviors such as clean faces [69]. The interventions did not report their objectives following the SMART (specific, measurable, attainable, relevant, time-bound) structure, nor presented specific indicators. The behaviors of focus per intervention are listed in the supporting information (S2 File), as well as the stream levels the interventions focused on.

Some publications describing the interventions explicitly mentioned the use of behavioral theories. One intervention focusing on schistosomiasis in China considered the Social Cognitive Theory of Multimedia Learning [71,72]. Another intervention addressing dengue in Colombia was designed following the stages of the Precaution Adoption Process Model [44]. Lastly, an intervention for Guinea-worm implemented in Nigeria, used the PRECEDE model [68]. Furthermore, the formative research conducted helped understand the contexts influencing people’s behaviors. This is reported in the following concept.

#### Concept 2: Public / people orientation focus

This refers to understanding what influences people’s behaviors (e.g. beliefs, attitudes, needs, wants) [15], through the collection and analysis of data using different research methods [26,29]. This informed understanding guides the development and implementation of actions along all phases and components of the intervention. This concept focuses on the research conducted to build that understanding of the audience, to ensure the interventions were oriented to the people.

All the interventions developed an understanding of the target audiences and their settings prior to design and implementation (see Table 3, S2 File). All the interventions carried out formative research, four of which considered the local cultural context [54,70–72,76–78,85]. This background work included focus groups discussions (FGDs) [48–50,55,78,90]; interviews [44,48–50,53,55,57,66,71,72,76,77,90]; the use of observational methods [50,54,57,71,72,76,77,89]; household surveys [50,55,72,76]; knowledge surveys [63,89]; knowledge and attitude surveys [53]; knowledge, attitudes, and practices (KAP) surveys [47–49,51,59,68,71,86]; knowledge, beliefs, and practices (KBP) surveys [44,64]; knowledge, intervention exposure, and reported behaviors surveys [58]; surveys to understand potential acceptability of a tangible product and sanitation practices [74], and a neighborhood background survey [55]. Additionally, one intervention used a questionnaire to understand past mass drug administration (MDA) experiences of drug distributors [48], and a cysticercosis intervention conducted a rapid ethnographic assessment and baseline prevalence studies [76,77]. Examples of other formative studies conducted by the interventions include a situational analysis to characterize and map the urban ecosystem [56,57], stakeholder analysis [55], and gender analysis [55]. Baseline entomological surveys were carried out by five dengue interventions [55,57,58,63,64].

#### Concept 3: Social offerings

Social offerings can take diverse forms (e.g. products, policies, environments, ideas, services) and can be positive (e.g. protection) or negative (e.g. restrictions) in nature [15]. A total of 14 interventions integrated aspects of this social marketing concept (see Table 3, S2 File). Tangible social offerings were provided by nine interventions [47–50,52,54,55,57,71], treatment was offered by seven [47–50,52,54,71], three offered tools to control the production of mosquitoes (e.g. garbage bags, lids and covers for water tanks) [55,57,58], and one intervention had an intangible offering, a cleaning method called “La Untadita” (The Little Dab) [58]. Structural offerings were facilitated by some interventions focusing on water, sanitation, and hygiene (WASH) [71,74,75,77], such as the building of household toilets giving householders a choice over the preferred design and placement within the household [76,77]. Some interventions used economic incentives [50,68,73,77,91] such as honorarium [50,68], distribution of profits [73] or partial subsidies [77]. The leprosy intervention sometimes provided reimbursement for travel costs and lost pay [91]. Other incentives such as rewards for children participating in hygiene stations [69], and disincentives in the form of criticism [47] were also present.

#### Concept 4: Relationship building

This last concept is present along all stages of the intervention and entails engaging and having valuable exchanges with different stakeholders, including but not exclusively with the selected publics of the intervention [15]. Eighteen interventions provided evidence of processes of engagement of stakeholders in the selection of priorities, development, or implementation of the activities (see Table 3, S2 File). Some interventions initiated stakeholder engagement from early stages [53,55,57,71,72] and others benefited from previous experience in the settings [54,58,74]. Two interventions explicitly mentioned the engagement of intersectoral groups [53,57]. Government officials or health authorities were approached by nine interventions [44,49–53,53,55,57,63,76]. Other publics were also engaged (see Table 4).

**Table 4:** Other publics engaged.

Public [reference]
University / research centers working in health [70–72,85]
Public control services [57]
Football clubs [69]
News media [48]
Health staff [49,50,52,53,57,86]
Actors [51,52]
School staff [47,48,50,54,57,58,68,74,86]
Parents or family members [53,54,69,72]
Local NGO [54]
Local music group [49]
Village heads [51,68,73] such as Kings [68,73]
Key community leaders [48,51,57,58,68] such as religious leaders [48,68]
Volunteers [48,49,55,58,68,77]

Community participation in activities was encouraged by many interventions [44,55,57,63–65,68–70,73,76]. Community groups [57,64,65] or health committees [63,68] were formed or mobilized by some. Creating and using local labor [77,86] and the participation of the research team in field activities [76] were also important to build relationships with the communities.

#### Technique 1: Integrated intervention mix

This refers to the types of strategies used to deliver value and influence behaviors [15]. These include the expanded marketing mix (i.e. product, price, place/distribution, promotion, partnerships, policy) and others such as advocacy, lobbying, and public and media relations [15,92]. All the interventions developed a mix of strategies that when integrated, provided value to the audiences (see Table 3, S2 File). The interventions included in this review used eight types of strategies: product, price, place, promotion, partnerships, policy, public relations, and community mobilization.

Both intangible and tangible products were used by the interventions. These included a cleaning method [58], toilets [77], garbage bags [55,57], and treatment [48–50,54,71]. Some interventions developed educational resources [47,53,64,68,70,72,89]. A capacity building component was present in eight interventions [49,50,52–54,63,71,76]. This included, training to local men in toilet construction [77], and training teachers, volunteers and health staff supporting in intervention delivery [49,50,52–54,63,71]. Health education was present in 16 of the 20 interventions [44,47,50,53,54,57–59,63–65,68,69,71,73,75–77,86,89].

In social marketing, price refers to what the audience has to exchange or give up to receive the benefits offered by the intervention [28,93]. The element of price was considered by some interventions [49,52,58,73–76]. This included the purchase of water filters [74], assuming the costs of materials and manpower [76,77], fear of adverse reactions to treatment [49], and psychological costs (e.g. fear of the disease) [51,52].

The place component of interventions included household visits to sell water filters [73,75], provide information or education [57,58,63,64,76], follow up on participants [44] or distribute treatment [49,50]. Two interventions increased the number of places where treatment was distributed [48,52].

Promotion was implemented by 19 interventions to raise awareness and disseminate their activities, this encompassed branding tactics [49–52,54,86], the development and use of promotional material [44,47–49,51–54,58,59,63–65,69–72,76], the use of mass media [44,48,50–52,58,69,86], sales promotion [73,75–77], endorsement [51,54,68,69,73], events [48,50,55,69,76,87] and other tactics [44,47,48,50,55,59,73,76]. See Table 5 for a detailed list of promotion activities.

**Table 5:** Type of promotion used by the interventions.

Category	Type of promotion activity [references]
Branding	Logos [50,51,69] Slogans [49,54,69] Mascot [69]
Promotion material	Flyers, pamphlets and brochures [49,50,65,71,77] Posters [49,50,52,53,59,69,77] Flipcharts [49,91] Danglers [50] Ribbon flags [50] Banners [58] Videos [49] Songs [49,70] Stickers [49,52,58,59] Branded cups [69] Branded wristband [69] Mascot tattoos [69] Branded key chains [58] T-shirts [50,54] Calendars with key activities and reminding behaviors [57,63] Comic book for promotion, not as augmented product [63] High transmission areas warning boards [47]
Use of mass media	Television advertisements [48,50,52,86] Television programs [48,51,52], such as interviews [48], discussions [48] and teledramas [51,52] Radio programs [44,52,58] Radio advertisements [48,50,52,58,86] Loudspeakers in rural areas [50] Newspaper [48,50,52]
Sales promotion	PHWs and town criers [73,74] Local toilet building supervisor [77]
Endorsement	Local Kings facilitated sales promotion and engagement [68,73] Schoolchildren who became schistosomiasis ambassadors [54] An actor who played a leprosy patient in a TV teledrama [51] Senior Indigenous Melbourne Football Club (MFC) players [69]
Events	Promotion of activities at Australian Football League games [69,86] Events [48,77] Community performances [69] Cinema presentations in rural and urban areas [50] Product/behavior demonstration [44,55,73,77]
Other	Announcements at churches and mosques [73] Testimonials of affected persons [48] Mosquito/schistosomes educational models/samples [44,47,59] Games [44,50,77] FPAs wore branded badges and fabric bags [50] Touring bicycle teams of 8–12 members wearing branded t-shirts drove around the villages [50]

Formal partnerships were explicitly mentioned by three interventions [51,52,69,70,76,80,83]. The trachoma intervention targeting aboriginal communities had several, for example, one to develop culturally appropriate resources that involved, among others, past and present aboriginal health workers; and another one with the Melbourne Football Club (MFC) to allocate two Indigenous players as trachoma ambassadors [69,70,80,83].

Policy was mentioned by two interventions. One obtained political support from local authorities to facilitate garbage collection [55], and another was designed considering the provincial policy on health education for schistosomiasis [53].

Public relations activities were mentioned by two interventions [50,64,65]. One conducted meetings with State and District administrations that resulted in press releases that facilitated participant engagement [50], and the second one delivered invitations to each household inviting to participate in the final community meeting [64,65].

Community mobilization strategies included environmental management activities [57], clean up campaigns [44,55,57,63], wash and cover tanks journeys [44] activities to educate neighbors [44], and in one intervention some villages initiated money collection to construct wells [68].

#### Technique 2: Competition analysis and action

This technique refers to internal and external factors that compete with or impede the publics from adopting the desired behavior, which includes other behaviors [15,24,26,29]. Six interventions provided information about this social marketing technique [49,51,52,64,66,68,69,73–75,85,86] (see Table 3, S2 File). One addressed fear to adverse reactions to treatment [49], and another addressed misconceptions about leprosy, guilt, and shame by leprosy sufferers and their lack of acceptance of the biomedical explanations of the disease, and the difficulty to recruit health care staff due to stigma [51,52].

Other competing factors mentioned by the interventions included the existence of community misperceptions of effective mosquito breeding control measures [62,64–66], preference to wells and alum instead of filters, and perception of water filters as having low efficacy [67,73–75]; and expectations on government agencies solving problems and beliefs in traditional methods [68]. One intervention focusing on trachoma, challenged embarrassment and community shame about personal hygiene, and that ‘dirty faces’ were considered as normal in young children in remote indigenous communities by staff from clinics, schools, and community workplaces.

#### Technique 3: Systematic planning and evaluation

This technique is about systematically planning the interventions, for example, through the use of planning models and frameworks and about using diverse methods for monitoring and evaluation [15]. Of the 20 interventions included in this study, one provided evidence of systematic planning only [54], six of evaluation only [48,49,53,57,63,89] and 13 of systematic planning and evaluation [44,47,50–52,55,58–61,64–66,68,71–78,86] (see Table 3, S2 File). Seven interventions explicitly mentioned the stages of the intervention processes [44,47,59–61,64–66,68,71,72,76–78] and three were planned considering their sustainability [54,55,76] and replicability [76,77]. Evaluation was explicitly mentioned by 19 interventions all of which conducted summative evaluations and seven also conducted mid-term evaluations [47,48,51,52,55,58,63,73–75].

The interventions evaluated: awareness [48,49,51–53], attitudes [47,49,53,59,71,86], beliefs [64], knowledge [44,47,49–52,58,59,64,68,71,73,75,86], self-reported practices [47,49,58,59,64], role of medical officers and health workers in the intervention [50], exposure to the intervention [50,58,63,86], effect of the intervention [44,53,55], resources used [57] and cost effectiveness [47], water filter purchasing patterns [74,75], cases identified through blood examination [53], adoption of preventive measures [68,71,89], compliance with treatment or MDA [47–50], self-reported adverse reactions to treatment [49], and infection rates [47,72]. The cysticercosis intervention evaluated increased household toilet building, use of and satisfaction with the new toilet [76,77]. Dengue interventions evaluated different elements with regards to mosquitoes, including, vector densities [57,58], larval indices [55], presence of larvae [64], larval densities [63], and presence of intradomiciliary breeding places [44]. One intervention evaluated processes of empowerment, collaboration and mobilization [57] and another community mobilization [55].

#### Technique 4: Insight-driven segmentation

This technique refers to identifying in the study population subgroups (segments) of people with similarities, selecting among them segments to target, and developing specific strategies to influence their behaviors [15]. Seventeen of the 20 interventions applied this technique (see Table 3, S2 File). Evidence of the use of segmentation was provided for dividing the market into smaller segments [44,47,52,65,69,86], offering reasons for target audience selection [47,58,59,69,86], developing different strategies for varied groups of people [44,47,50,52,65,69,76,86], and by mentioning how insights from daily lives of the participants or from formative research were incorporated in the design or delivery of the intervention [44,48,49,53,55,58,65,72,73,89]. For example, one intervention mentioned that they used drama techniques (e.g. role-plays, storytelling) because they are part of African performance traditions [54]. A Guinea worm intervention promoting the purchase and use of monofilament nylon cloth filters allocated the salesforce to sectors considering the usual agenda of primary health workers (PHWs) [67,73–75]. Another intervention classified participants according to the stages of the Precaution Adoption Process Model and implemented activities according to the stages at which they were [44].

#### Technique 5: Co-creation

This technique refers to strategies used to facilitate the participation of the main publics, for example, in the development of value propositions [15]. Twelve interventions incorporated this technique [27,31–33,35,41,43,46,48,49,51,54] (see Tables 3 and 6, S2 File). Examples of co-creation strategies include having workshops with teachers and schoolchildren to learn how to develop educational videos and to create them, and feedback meetings at schools and communities to share results from formative research and to envision possible activities to undertake to reduce transmission [54]. Other examples are the use of community meetings to co-create messages and layout of pamphlets, and photo sessions to develop a photonovel using pictures from community members carrying out control activities [65].

**Table 6:** Examples of co-created activities implemented by the interventions.

Author, year [reference]	Activities
Brieger et al., 1989 [74]; Brieger et al., 1986 [73]; Brieger et al., 1990 [75]; Adeniyi and Brieger, 1983 [67]	Young Tailors Association produced filters. Primary Health Workers (PHWs) involved in all phases of the intervention.
Dickey et al., 2015 [77]; Dickey et al., 2016 [78]; Dickey, 2014 [76]	Homeowners decided toilet placement and responsible for the toilet building materials and construction.
Caprara et al., 2015 [57]; Alfonso-Sierra et al., 2016 [56]	Workshops with varied stakeholders, including community members, to discuss the results of the situational analysis and planned actions considering the needs of each locality.
Ramaiah et al., 2006 [50]	NA
Atkinson et al., 2014 [69]; Lange et al., 2014 [85]; Lange et al., 2017 [86]; Baunach et al., 2012 [70]; Stanford et al., 2016 [87]; Lange et al., 2016 [84]; Taylor et al., 2012 [88]; Lange et al., 2012 [82]; Lange et al., 2015 [83]; Jones et al., 2015 [79]; Lange, JR Atkinson, et al., 2013 [80]; Lange, J Atkinson, et al., 2013 [81]	Trachoma Story Kits co-developed with the Ngumpin Reference Group (NRG) in a 12-month consultation process. The NRG and Aboriginal Health Workers from the Katherine West Health Board (KWHB) recommended where to place the resources used (e.g. Trachoma Story Kits).
King et al., 2011 [48]	NA
Krentel et al., 2006 [49]	Collaboration of a local music group to develop a song. Drug delivery method decided between the health workers and the villagers.
Salgado, 1993 [51]; Williams et al., 1998 [52]; Wong, 2002 [117]; Brown, 2006 [91]	NA
Freudenthal et al., 2006 [54]	Schoolchildren participated in developing slogans for t-shirts. Grade 6 pupils conducted household sanitation survey. Modification to the survey done by them and the teachers. Video recorded dramas, songs and dances by the schoolchildren. Community members created safe swimming places.
Bieri, Yuan, et al., 2013 [72]; Bieri, Gray, et al., 2013 [71]	Brainstorming sessions with a multidisciplinary team to draft the cartoon narrative. Chinese scientists consulted on Chinese cultural aspects. Key informants, including teachers, doctors, parents and schoolchildren contributed to video development. Chinese educators participated in the development and piloting of KAP questionnaire.
Escudero-Támara and Villareal-Amaris, 2015 [44]	NA
Abeyewickreme et al., 2012 [55]; Arunachalam et al., 2010 [90]	Volunteer groups organized environment cleaning campaigns in their clusters. Local authorities, health workers (PHI, MOH), and religious leaders of the area participated.
NK Ibrahim et al., 2009 [59–61]	NA
Hu et al., 2005 [47]	NA
Adeyanju, 1987 [68]	Regular community meetings by the village health workers (trainees) to share with the community what they had learned, identify community problems and identify possible solutions.
Lloyd et al., 1992 [64]; Winch et al., 1991 [66]; Lloyd et al., 1994 [65]; Kendall et al., 1991 [62]	During community meetings, residents designed the layout and messages for pamphlets specific to their target group. Two photo sessions: one to determine the photonovel storyline and the second to select the pictures and retake if needed.
Leontsini et al., 1993 [63]; Kendall et al., 1991 [62]	Community meetings and meetings with health committees were used to plan activities. Health committees created per suggestion of residents. These organized and carried out clean-up campaigns and sewage maintenance work.
Yuan et al., 2005 [53]	Students designed posters to motivate behavior change among peers.
Fernández et al., 1998 [58]	NA
Yuan et al., 2000 [89]	NA

Note: This is a summary of the co-created activities per intervention. Further details in S2 File.

### Behavioral determinants, behavior changes, and health outcomes

Behavioral determinants are considered mediating outcomes to influence behavior [17]. Changes in behavior are needed to attain positive health outcomes (e.g. reducing infection rates). In this review, the five public health strategic interventions prioritized by the WHO are used to present the behavior focus and outcomes reached by the interventions. These outcomes were not predefined by the study; instead, these were coded inductively according to what the interventions reported. Due to the variety of outcomes considered in this review, comparisons nor meta-analysis were carried out.

Changes in behavioral determinants, behavior change, or health outcomes were reported by the 20 interventions (See Table 7). The outcomes on behavioral determinants included awareness (n = 5) [48,49,51,52,54,86]; attitudes (n = 5) [49,53,58,86]; beliefs (n = 1) [44]; knowledge (n = 17) [44,47–50,53,57–59,63,64,68,71,73–75,77,86,89]; policy (n = 2) [49,57]; structure (n = 6) [52,54,55,68,68,73–75,77] such as increasing the network of field clinics, improving garbage collection services, facilitating the construction of sanitary wells or safe swimming places, and inspiring teachers to incorporate schistosomiasis education in primary schools; and skills [86], more precisely increased in the ability of clinic staff to screen for trachoma, and of schools and community staff to teach others about trachoma prevention. Changes in behavior were reported by 19 interventions. One only reported behavioral determinant outcomes [54]. Four interventions reported health outcomes, including prevalence [47,52,86], incidence [71], and re-infection rates [47].

**Table 7:** Outcomes reported per intervention according to WHO NTD priority strategies.

WHO Priority Strategy	Neglected Tropical Disease	Author, year [reference]	Outcomes Reported		
			Behavioral Determinants	Behavior Change	Health Outcomes
Preventive chemotherapy and transmission control (PCT)	Lymphatic filariasis	Ramaiah et al., 2006 [50]	Knowledge	✓	✕
		King et al., 2011 [48]	Awareness Knowledge	✓	✕
		Krentel et al., 2006 [49]	Awareness Attitudes Knowledge Policy	✓	✕
	Schistosomiasis	Yuan et al., 2005 [53]	Attitudes Knowledge	✓	✕
Innovative and intensified disease management (IDM)	Leprosy	Salgado, 1993 [51]; Williams et al., 1998 [52]; Wong, 2002 [117]; Brown, 2006 [91]	Awareness Structural	✓	✓
Vector ecology and management (VEM)	Dengue	Caprara et al., 2015 [57]; Alfonso-Sierra et al., 2016 [56]	Knowledge Policy	✓	✕
		Escudero-Támara and Villareal-Amaris, 2015 [44]	Beliefs Knowledge	✓	✕
		Abeyewickreme et al., 2012 [55]; Arunachalam et al., 2010 [90]	Structural	✓	✕
		NK Ibrahim et al., 2009 [59–61]	Attitudes Knowledge	✓	✕
		Lloyd et al., 1992 [64]; Winch et al., 1991 [66]; Lloyd et al., 1994 [65]; Kendall et al., 1991 [62]	Knowledge	✓	✕
		Leontsini et al., 1993 [63]; Kendall et al., 1991 [62]	Knowledge	✓	✕
		Fernández et al., 1998 [58]	Awareness Knowledge	✓	✕
Water, sanitation and hygiene (WASH)	Cysticercosis	Dickey et al., 2015 [77]; Dickey et al., 2016 [78]; Dickey, 2014 [76]	Knowledge Structural	✓	✕
	Guinea-worm disease	Brieger et al., 1989 [74]; Brieger et al., 1986 [73]; Brieger et al., 1990 [75]; Adeniyi and Brieger, 1983 [67]	Knowledge Structural	✓	✕
		Adeyanju, 1987 [68]	Knowledge Structural	✓	✕
	Schistosomiasis	Yuan et al., 2000 [89]	Knowledge	✓	✕
	Soil-transmitted helminths	Bieri, Yuan, et al., 2013 [72]; Bieri, Gray, et al., 2013 [71]	Knowledge	✓	✓
	Trachoma	Atkinson et al., 2014 [69]; Lange et al., 2014 [85]; Lange et al., 2017 [86]; Baunach et al., 2012 [70]; Stanford et al., 2016 [87]; Lange et al., 2016 [84]; Taylor et al., 2012 [88]; Lange et al., 2012 [82]; Lange et al., 2015 [83]; Jones et al., 2015 [79]; Lange, JR Atkinson, et al., 2013 [80]; Lange, J Atkinson, et al., 2013 [81]	Awareness Attitudes Knowledge Skills	✓	✓
Combined: PCT and WASH	Schistosomiasis	Freudenthal et al., 2006 [54]	Structural	✕	✕
		Hu et al., 2005 [47]	Attitudes Knowledge	✓	✓
**Total**			**20**	**19**	**4**

### Self-reported results of the interventions

Behavior influence is central to social marketing [15,26,27]. According to the evaluation results reported by 19 interventions, overall, they were successful in changing behaviors related to NTD prevention and control. A direct relationship between the number of concepts or techniques used and the success of the interventions in reaching the planned outcomes was not observed. It was not possible to quantify the effectiveness of the interventions due to the heterogeneity of the measures and outcomes of the interventions. For this reason, it was also not feasible to present effectiveness according to the concepts and techniques used. However, the interventions were successful in achieving positive outcomes, and these are reported in this section following the five WHO public health strategic interventions for NTDs (see S3 File).

The interventions focusing on PCT were able to increase the number of participants receiving [50], and taking treatment [48–50,53], MDA coverage [48], and adherence to screening [53]. These interventions used at least three concepts and two techniques.

The intervention focusing on IDM increased the number of individuals seeking treatment and early detection [52]. It reported a decrease in the national prevalence rate for leprosy and was successful in reaching most of its objectives. It used all concepts and techniques, except for the technique co-creation.

Interventions focusing on VEM were successful in eliminating breeding places [44,64] and reducing vector densities [55,57]. One reported a relative reduction of larval infestation indices in the intervention neighborhoods [63]. The “La Untadita” intervention reported that changes in human behavior were achieved, but the level of reduction in the overall infestation indices was not enough for adequate control of mosquitoes [58]. The interventions not conducting entomological evaluations reported increasing the number of participants in the stages six (action) and seven (maintenance) of the Precaution Adoption Process Model [44], and a positive impact on knowledge and practices related to dengue fever (DF) prevention [59]. The interventions focusing on VEM [44,55,57–59,63,64] used at least two concepts and two techniques.

Interventions focusing on WASH were able to motivate the communities to build toilets and use them [77] and to purchase and use water filters [75]. These interventions successfully prompted children to clean their faces [69,86], hand wash after toilet use [71], and decrease contact with unsafe water [89]. In one intervention, villagers adopted preventive measures, and village health volunteers referred cases to the health center [68]. An intervention addressing soil-transmitted helminths “was associated with 50% efficacy (95% CI, 30 to 65) in preventing infection” [71]. Of these, almost all used the four social marketing concepts and four [69–72,76,86] or five [68,73–75] techniques.

Of the two interventions using combined PCT and WASH strategies, one only reported behavioral determinants [54]. The other one was successful in decreasing the frequency of water contact among women and children, but not among men, and increased compliance with chemotherapy in the three segments [47]. Both applied four concepts and three techniques, none addressed competition.

### Methodological quality

**Fig 3:**
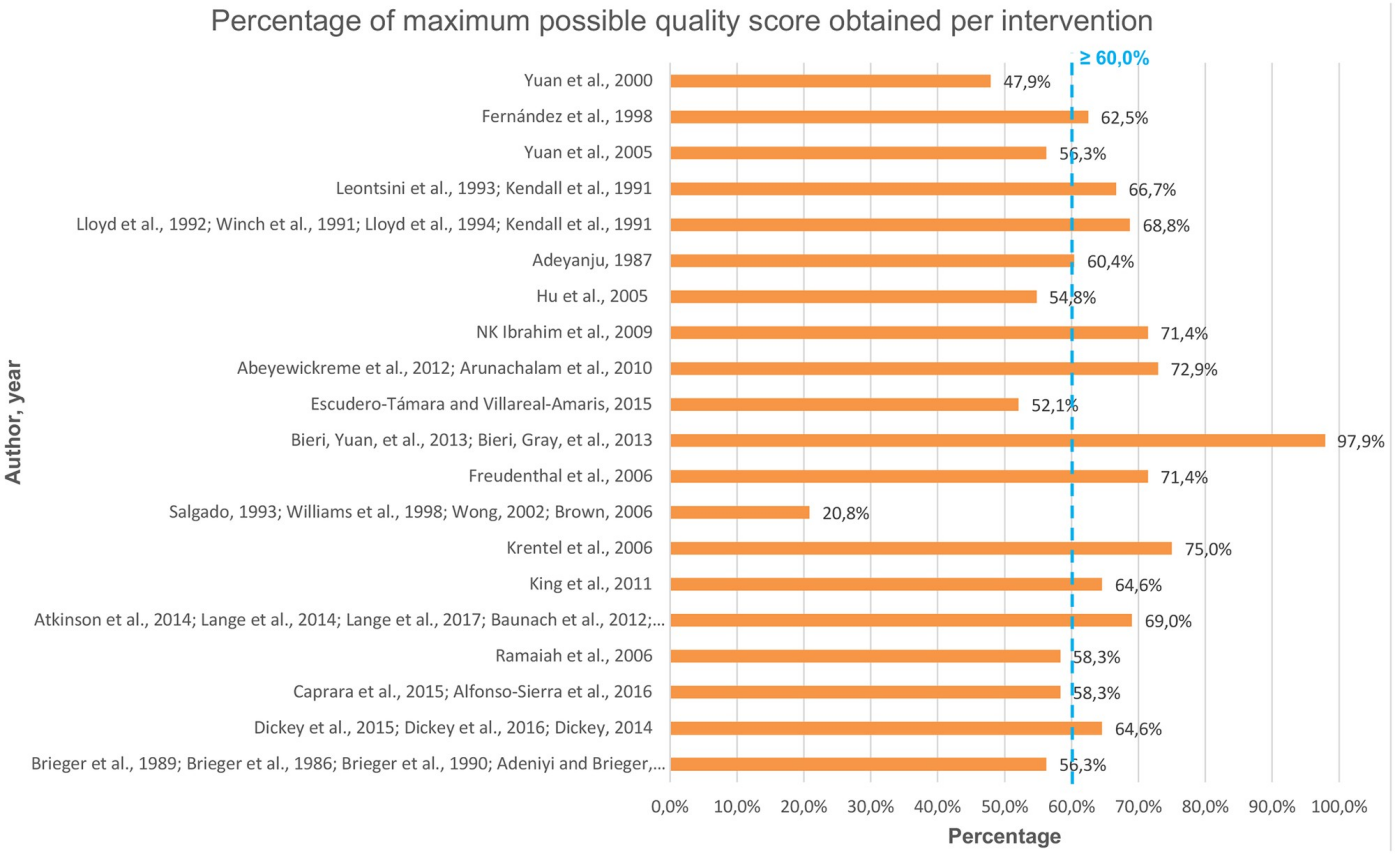
Results quality assessment.

According to the results of the QATSDD critical appraisal tool [40], the average quality score for all papers describing the interventions was 62.5% (see Fig 3). The methodological quality scores ranged from 20.8% [51,52] to 97.9% [71,72]. Most of the interventions (n = 12) achieved a score of 60% or higher (see Fig 3, S4 File). The lowest scores were reported on the criteria “assessment of reliability of analytical process” not applicable for quantitative studies, and “statistical assessment of reliability and validity of measurement tool(s)” not applicable to qualitative studies. Criteria for which most studies score high were “statement of aims/objectives in main body of report” and “clear description of research setting”, both applicable to quantitative, qualitative, and mixed methods studies.

Two interventions achieved scores lower than 50%, namely the intervention addressing leprosy in Sri Lanka [52], and an intervention focused on schistosomiasis implemented in China [89]. The former reported positive changes in behavioral determinants, behavior change, and health outcomes (i.e. prevalence), but did not report their methodological decisions and processes thoroughly. The latter received low scores in most of the criteria assessed, mainly for providing minimal descriptions and justification of their methodological processes. In contrast, only one intervention achieved a quality score higher than 80% [71,72]. This intervention focused on soil-transmitted helminths in China, obtained a 97.9% score. It thoroughly reported the methods used and rationale, as well as provided data on behavioral determinants, behavior change, and health (i.e. incidence) outcomes.

## Discussion

Social marketing interventions for the prevention and control of NTDs have focused on eight of the 17 diseases prioritized in the WHO NTD Roadmap [34]. Although soil-transmitted helminths are the largest cause of NTD-related disability-adjusted life-years (DALYs), only one of the 20 interventions focused on this disease, whereas seven focused on dengue which is only the sixth leading cause of NTD-related DALYs [94]. The 20 interventions relate to four out of five of the WHO priority strategies for preventing and controlling NTDs, with one intervention addressing behaviors related to IDM, and none focusing on veterinary public health measures. Although NTDs represent a major burden in sub-Saharan Africa [94,95], only three of the 20 interventions identified were from this region.

To our knowledge, this review is the first to use the Hierarchical Model of Social Marketing [15]. Overall the interventions integrated most concepts and techniques of the model. More than 15 interventions incorporated the concepts “relationship building” and “public / people orientation focus”, and the technique “insight-driven segmentation”. Only the technique “competition analysis and action” had less than ten interventions applying it.

A direct comparison with findings from other reviews is not possible, except to the extent that some elements of the Hierarchical Model of Social Marketing are similar or equivalent to the social marketing benchmarks [26,29,30] on which the model is grounded. The concept “public / people orientation focus” is equivalent to the benchmark “citizen orientation”. Other social marketing systematic reviews of health interventions also found that the interventions well incorporated this benchmark [17,24]. The technique “insight-driven segmentation” is equivalent to the benchmarks “insight” and “segmentation”. These benchmarks have also been among the most commonly used in social marketing global health interventions [17]. The technique “competition analysis and action” is equivalent to the benchmark “competition”. Other systematic reviews of social marketing also found a low level of adherence to this benchmark [17,24,35,36]. There is no equivalent benchmark to the concept “relationship building”.

Social marketing concepts and techniques should be adapted to the specific characteristics of the context intervened. Nevertheless, some general insights that could be considered by any social marketing NTD intervention are drawn from the studies included in this review. One is that all actions and their outputs should be guided by a strong understanding of the audience and the local context. This was an important characteristic of the interventions included in this review. Other systematic reviews of social marketing have also highlighted the benefits of understanding the publics before developing interventions, such as being better equipped to influence behaviors [17,19]. Therefore, NTD social marketing interventions should understand the audience and context before designing possible solutions.

Another social marketing concept to highlight from the findings of this study is relationship building. Engaging audiences from the early stages of the intervention could be favorable to achieve NTD prevention and control outcomes. Primary beneficiaries, as well as other stakeholders, could cooperate in designing and delivering parts of the intervention [96]. This was observed in several interventions included in this study. Their involvement could help develop a sense of ownership [8,97], design realistic solutions based on local knowledge and capacities [98], and facilitate the sustainability of actions [55,97]. This is consistent with recommendations from other studies and reports calling for placing engagement of communities at the center [98], as well as for promoting intersectoral and cross-sectoral collaboration to prevent, control, and eliminate NTDs [8,99].

Regarding social marketing techniques, the intervention mix of strategies should be integrated. The strategies should not be implemented in isolation, but rather as complementary to each other. The interventions assessed in this review combined varied strategies to achieve their planned outcomes. This is something to replicate by other social marketing interventions. Additionally, taking into consideration internal and external factors that impede audiences adopting the desired behavior is important. Considering competing factors such as embarrassment, shame, and misconceptions helped some interventions in this review attain their planned outcomes. However, analysis of the competition and acting upon that was a missed opportunity for most interventions reviewed. Additionally, NTD social marketing interventions that showed positive results, more often than not, integrated health education, had a capacity building component and were culturally appropriate. These elements should also be considered in planning and delivering future interventions.

This study identified the need to expand the application of social marketing to address behaviors related to IDM and veterinary public health measures. Most of the interventions included in this review focused on behaviors associated with the priority public health strategic interventions that receive more considerable attention from the NTD community and funders, namely PCT, VEM, and WASH. The focus on these could be the result of imbalances observed at the global policy level, where diseases that were once considered tool-deficient were found to be less prioritized [100]. Therefore, moving IDM and veterinary public health measures up in the global policy agenda could help in driving more resources to implement social marketing interventions focused on behaviors related to these strategic interventions.

Social marketing interventions can focus simultaneously on more than one of the five priority strategies to prevent and control NTDs. Although this study only found two interventions combining PCT and WASH strategies to address schistosomiasis, other interventions could have also benefited from using multiple priority strategies. Only one intervention addressing schistosomiasis screened and provided treatment for intestinal helminths in parallel to the disease of focus, but it did not implement measures beyond that, missing the opportunity to address both diseases simultaneously. The benefits of clustering NTDs have been highlighted in the literature, including its cost-effectiveness [103]. Combining strategies may provide more effective control of one or several diseases, even if one strategy dominates [101,102].

Only four of the 20 interventions self-identified as social marketing interventions. This raises questions as to whether public health programs use social marketing without knowing it or decide not to label it as such. The challenges in identifying social marketing interventions have been raised by other scholars [17,19,104,105]. Quinn et al. [105] found that some self-labeled social marketing interventions were only focused on disseminating health messages and, therefore, were not social marketing. While conducting reviews, other scholars have found that the characteristics of the interventions rather than the label help determine whether they have applied social marketing elements [104]. An establishing standard and measurable criteria to operationalize the social marketing definition and elements (e.g. benchmark, concepts, techniques) is warranted.

According to the QATSDD critical appraisal tool, overall, the studies in this review were of variable quality. For this reason, the outcomes reported should be considered with caution, especially for studies with lower quality scores. Social marketing interventions addressing NTDs need to improve their quality of report and provide more details about the methodological rationale guiding their actions, as specified in the QATSDD.

Behavior change is necessary to attain NTD outcomes. This has been highlighted in reports of progress towards NTD control, elimination and eradication targets [8,101,102,106]. The five WHO public health strategic interventions involve influencing behaviors of downstream (individual), midstream (e.g. communities), and upstream (e.g. policy makers) audiences. Social marketing as an approach to behavior change can contribute in promoting voluntary behavior change at all these levels [16,107]. Incorporating social marketing elements in developing interventions for NTDs would also be aligned with the new NTD roadmap for the period 2021–2030, prioritizing integrated approaches and cross-sectoral collaboration [108]. This has also been postulated at the global NTD policy level, more precisely by the Director General of Mexico’s National Center for Disease Prevention and Control during the 2017 Global Partners Meeting on NTDs when he said: “for us, it is important to have a tactical approach and continuous action in epidemiological surveillance, entomological surveillance, health promotion, and social marketing, insecticide resistance and monitoring that resistance, the vector control, and the health care guidelines” [109].

However, NTD policies do not explicitly mention social marketing as an approach to behavior change as do other national and global health policies or reports suggesting its use for health promotion and disease prevention [110–115]. While further research is warranted, the interventions reviewed in this study show that social marketing has been used successfully in NTD prevention and control. Our findings show that social marketing is aligned with the current priority strategies for the prevention and control of NTDs. Thus, ongoing discussions to formulate the targets and milestones of the new global Roadmap for NTDs, presently under development by the WHO in consultation with the NTD community, should integrate social marketing as an approach to overcome these diseases.

### Strengths and limitations

The use of the Hierarchical Model of Social Marketing [15] for data extraction and analysis was both a strength and a limitation in this study. It is the first time it was used in a systematic review of social marketing, and while this is thus innovative, this innovation meant a lack of precedent, and we found overlapping concepts and techniques in the model. An additional limitation was found in the difficulty to operationalize the elements of the framework due to their broad definitions. The challenges in operationalizing social marketing criteria have been raised in other studies [17,19,104]. It is recommended that future systematic reviews using the Hierarchical Model of Social Marketing, test and develop tools and methods to operationalize the concepts and techniques better. In doing this, studies should adapt the model to respond to the context of LMICs, settings with the highest burden of NTDs.

Moreover, this review focused on the 17 NTDs prioritized by the WHO at the time of study design, three new diseases (i.e. chromoblastomycosis and other deep mycoses, scabies and other ectoparasites, snakebite envenoming) added to the WHO NTD portfolio as of 2017 were not included. The reason for this was that these diseases were included after the study selection process was finalized, and when data extraction was in progress.

A notable limitation in this study was the absence of a validated measurement tool for the Hierarchical Model of Social Marketing. Similarly, this review is limited by the fact that social marketing interventions may have been missed simply because they lacked explicit terminology related to social marketing. We aimed to compensate for these by having experts in social marketing involved in the study selection and coding processes, and by using broad terms in the search strategies. These strategies did not restrict papers to any specific language. Where data were missing, we contacted the authors of the papers. However, not all authors we contacted in the study selection process responded to inquiries, and due to missing information, some interventions could not be included. Therefore, as reported in the PRISMA diagram, this review did not include some interventions due to missing data.

### Conclusion

NTDs have gained recognition globally as a result of the interplay of various determinants of political priority [100]. These include having a common set of goals and the involvement of actors from multiple sectors to reach them [8,34,100]. Progress has been made to reach global NTD targets, but collaboration across sectors and disciplines has been highlighted as fundamental to attain global goals [8,116]. Social marketing can contribute in reaching NTD targets. Evidence from this study shows that social marketing has been successfully used to address behaviors related to most of the public health strategic interventions for NTDs.

Moving forward, social marketing interventions for the prevention and control of NTDs need to be grounded on an understanding of the audience and adapted to the contexts. They should also build stakeholder relationships as early as possible and involve the publics. Elements of the intervention mix should be integrated and mutually supportive. Incorporating health education and capacity building, as well as being culturally appropriate, is also important. Challenges that remain include expanding the application of social marketing to address behaviors related to IDM and veterinary health measures as well as addressing several diseases with similar characteristics simultaneously, and combining NTD priority strategies. Addressing these challenges, building from positive experiences highlighted in this review, could be beneficial to attain NTD prevention and control outcomes.

## Supporting information

S1 File Search strategies. (DOCX)

S2 File Summary of the interventions. (DOCX)

S3 File Intervention outcomes per priority strategy and disease. (DOCX)

S4 File Results of the quality assessment per intervention. (DOCX)

S5 File List of abbreviations. (DOCX)

S1 Checklist PRISMA checklist. (DOC)
