# Supplementary material for: Social marketing interventions for the prevention and control of neglected tropical diseases: A systematic review
Source: PLoS Negl Trop Dis. 2020 Jun 17;14(6):e0008360. doi: 10.1371/journal.pntd.0008360 (PMC7299328; doi:10.1371/journal.pntd.0008360)
Supplement: S1 File — (DOCX) [file pntd.0008360.s001.docx]

**S1. Search strategies**

**PubMed**

| **#** | **Searches** | **Results** |
| --- | --- | --- |
| **1** | (Ulcer, Buruli) OR mycobacterium ulcerans | 1,169 |
| **2** | ((Disease, Chagas) OR american trypanosomiasis) OR trypansosoma cruzi | 14,229 |
| **3** | (dengue) OR mosquito | 52,709 |
| **4** | (chikungunya) OR Chikungunya virus | 3,189 |
| **5** | (((dracunculiasis) OR guinea-worm disease) OR dracunculus medinensis) OR Dracunculiases | 844 |
| **6** | (((((((((((echinococcosis) OR echinococcus granulosus) OR cystic echinococcosis) OR hydatidosis) OR hydatid disease) OR alveolar echinococcosis) OR echinococcus multilocularis) OR polycystic echinococcosis) OR echinococcus vogeli) OR unicystic echinococcosis) OR echinococcus oligarthrus) OR Echinococcoses | 16,008 |
| **7** | (((((((((((foodborne trematodiases) OR trematode worms) OR flukes) OR freshwater snail) OR freshwater fish) OR clonorchis sinensis) OR opisthorchis viverrini) OR opisthorchis Felineus) OR fasciola hepatica) OR fasciola gigantica) OR paragonimus) OR Disease, Foodborne | 68,193 |
| **8** | (((((((human african trypanosomiasis) OR sleeping sickness) OR trypanosoma brucei gambiense) OR tsetse flies) OR glossina genus) OR trypanosoma brucei rhodesiense) OR nagana) OR Trypanosomiases, African | 8,354 |
| **9** | (((((((leishmaniasis) OR visceral leishmaniasis) OR cutaneous leishmaniasis) OR mucocutaneous leishmaniasis) OR kala-azar) OR leishmania parasites) OR phlebotomine sandflies) OR Leishmaniases | 25,901 |
| **10** | ((((leprosy) OR hansen's disease) OR mycobacterium leprae) OR Leprosies) OR Hansen Disease | 28,111 |
| **11** | ((((((((((lymphatic filariasis) OR elephantiasis) OR nematodes) OR roundworms) OR wuchereria bancrofti) OR brugia malayi) OR brugia timori) OR culex) OR anopheles) OR aedes) OR Elephantiases, Filarial | 99,638 |
| **12** | (((((onchocerciacis) OR river blindness) OR onchocerca volvulus) OR simulium) OR blackflies) OR Onchocerciases | 5,512 |
| **13** | (rabies) OR Lyssa | 11,274 |
| **14** | ((((((((((((schistosomiasis) OR parasitic worms) OR blood flukes) OR trematode worms) OR Intestinal schistosomiasis) OR schistosoma mansoni) OR schistosoma japonicum) OR schistosoma mekongi) OR schistosoma guineensis) OR schistosoma intercalatum) OR urogenital schistosomiasis) OR schistosoma haematobium) OR Schistosomiases | 117,465 |
| **15** | ((((((((((soil-transmitted helminthiases) OR soil-transmitted helminth infection) OR roundworm) OR ascaris lumbricoides) OR whipworm) OR trichuris trichiura) OR hookworms) OR necator americanus) OR ancylostoma duodenale) OR Helminthiases) OR Infections, Nematomorpha | 130,821 |
| **16** | (((((((((((taeniasis/cysticercosis) OR taeniasis) OR cysticercosis) OR tapeworms) OR taenia solium) OR pork tapeworm) OR taenia saginata) OR beef tapeworm) OR taenia asiatica) OR cysticerci) OR cysticercus) OR Cysticercoses | 16,558 |
| **17** | ((trachoma) OR chlamydia trachomatis) OR Trachomas | 16,325 |
| **18** | ((((endemic treponematoses) OR yaws) OR treponema pallidum) OR pertenue) OR Frambesia | 4,699 |
| **19** | ((((Disease, Neglected) OR "neglected tropical disease") OR "tropical disease") OR "neglected disease") OR NTD | 14,404 |
| **20** | 1 OR 2 OR 3 OR 4 OR 5 OR 6 OR 7 OR 8 OR 9 OR 10 OR 11 OR 12 0R 13 OR 14 OR 15 OR 16 OR 17 OR 18 OR 19 | 335,835 |
| **21** | ((Marketing, Social) OR Communication, Health) OR Promotion, Health | 220,223 |
| **22** | (((((Campaign, Health) OR Campaign) OR Intervention) OR Program) OR Strategy) OR Project | 1,581,579 |
| **23** | #20 AND #21 AND #22 AND ("1971/01/01"[PDAT] : "2017/04/26"[PDAT]) | 1,088 |

**EbscoHost**

**Limiters** - Scholarly (Peer Reviewed) Journals; Published Date: 19710101-20170426; Publication Type: Academic Journal; Publication Type: Academic Journal; Publication Type: Academic Journal; Publication Year: 1971-2017; Publication Type: Peer Reviewed Journal

**Search modes** - Boolean/Phrase

**Databases** - CINAHL, Business Source Premier, Communication & Mass Media Complete, GreenFILE, Psychology and Behavioral Sciences Collection, PsycINFO

| **#** | **Search Terms** | **Results** |
| --- | --- | --- |
| **1** | "social marketing" OR "health promotion" OR "health communication" | 100,804 |
| **2** | buruli ulcer OR mycobacterium ulcerans OR chagas disease OR american trypanosomiasis OR trypanosoma cruzi OR dengue OR mosquito OR chikungunya OR dracunculiasis OR guinea worm disease OR dracunculus medinensis OR echinococcosis | 4,994 |
| **3** | echinococcus granulosus OR cystic echinococcosis OR hydatidosis OR hydatid disease OR alveolar echinococcosis OR echinococcus multilocularis OR polycystic echinococcosis OR echinococcus vogeli OR unicystic echinococcosis OR echinococcus oligarthrus OR foodborne trematodiases OR trematode worms | 249 |
| **4** | flukes OR freshwater snails OR freshwater fish OR clonorchis sinensis OR opisthorchis viverrini OR opisthorchis Felineus OR fasciola hepatica OR fasciola gigantica OR paragonimus OR human african trypanosomiasis OR sleeping sickness OR trypanosoma brucei gambiense | 2,768 |
| **5** | tsetse flies OR glossina genus OR trypanosoma brucei rhodesiense OR nagana OR leishmaniasis OR visceral leishmaniasis OR cutaneous leishmaniasis OR mucocutaneous leishmaniasis OR kala-azar OR phlebotomine sandflies OR leprosy OR hansen's disease | 2,123 |
| **6** | mycobacterium leprae OR lymphatic filariasis OR elephantiasis OR nematodes OR roundworms OR ( roundworms or nematodes ) OR wuchereria bancrofti OR brugia malayi OR brugia timori OR culex OR anopheles OR aedes | 3,885 |
| **7** | onchocerciasis OR river blindness OR onchocerca volvulus OR simulium OR blackflies OR rabies OR schistosomiasis OR parasitic worms OR blood fluke OR trematode worms OR Intestinal schistosomiasis OR schistosoma mansoni | 2,537 |
| **8** | schistosoma japonicum OR schistosoma mekongi OR schistosoma guineensis OR schistosoma intercalatum OR urogenital schistosomiasis OR schistosoma haematobium OR soil-transmitted helminthiases OR soil-transmitted helminth infection OR roundworm OR ascaris lumbricoides OR whipworm OR trichuris trichiura | 858 |
| **9** | hookworms in humans OR hookworms OR necator americanus OR ancylostoma duodenale OR taeniasis/cysticercosis OR taeniasis OR cysticercosis OR tapeworm OR taenia solium OR pork tapeworm OR taenia saginata OR beef tapeworm | 741 |
| **10** | taenia asiatica OR cysticerci OR trachoma OR chlamydia trachomatis OR endemic treponematoses OR yaws OR treponema pallidum OR pertenue | 3,429 |
| **11** | neglected tropical diseases OR tropical diseases OR neglected diseases OR ntds | 2,712 |
| **12** | 2 OR 3 OR 4 OR 5 OR 6 OR 7 OR 8 OR 9 OR 10 OR 11 | 21,199 |
| **13** | intevention OR campaign OR program OR project OR strategy | 1,775,092 |
| **14** | 1 AND 12 AND 13 | 188 |

**ProQuest**

Databases: ABI/INFORM Global‎, ABI/INFORM Trade & Industry‎, ERIC, MEDLINE

Limiters: Peer reviewed, Scholarly journals

Publication date: 01 Jan 1971 - 26 Apt 2017

| **#** | **Search** | **Results** |
| --- | --- | --- |
| **1** | all("social marketing") OR all("health promotion") OR all("health communication") | 70,731 |
| **2** | all("neglected tropical diseas*") OR all("neglected diseas*") OR all("tropical diseas*") AND all("NTD*") | 7,363 |
| **3** | all("buruli ulcer") OR all("mycobacterium ulcerans") OR all("chagas disease") OR all("american trypanosomiasis") OR all("trypansosoma cruzi") OR all("dengue") OR all("mosquito") OR all("chikungunya") | 45,362 |
| **4** | all("dracunculiasis") OR all("guinea-worm disease") OR all("dracunculus medinensis") OR all("echinococcosis") OR all("echinococcus granulosus") OR all("cystic echinococcosis") OR all("hydatidosis") OR all("hydatid disease") OR all("alveolar echinococcosis") OR all("echinococcus multilocularis") | 10,325 |
| **5** | all("polycystic echinococcosis") OR all("echinococcus vogeli") OR all("unicystic echinococcosis") OR all("echinococcus oligarthrus") OR all("foodborne trematodiases") OR all("trematode worms") OR all("flukes") OR all("freshwater snail") OR all("freshwater fish") OR all("clonorchis sinensis") | 5,711 |
| **6** | all("opisthorchis viverrini") OR all("opisthorchis Felineus") OR all("fasciola hepatica") OR all("fasciola gigantica") OR all("paragonimus") OR all("human african trypanosomiasis") OR all("sleeping sickness") OR all("trypanosoma brucei gambiense") OR all("tsetse flies") OR all("glossina genus") | 8,472 |
| **7** | all("trypanosoma brucei rhodesiense") OR all("nagana") OR all("leishmaniasis") OR all("visceral leishmaniasis") OR all("cutaneous leishmaniasis") OR all("mucocutaneous leishmaniasis") OR all("kala-azar") OR all("leishmania parasites") OR all("phlebotomine sandflies") | 18,278 |
| **8** | all(leprosy) OR all("hansen's disease") OR all("mycobacterium leprae") OR all("lymphatic filariasis") OR all("elephantiasis") OR all("nematodes") OR all("roundworms") OR all("wuchereria bancrofti") OR all("brugia malayi") | 31,506 |
| **9** | all("brugia timori") OR all("culex") OR all("anopheles") OR all("aedes") OR all("onchocerciasis") OR all("river blindness") OR all("onchocerca volvulus") OR all("simulium") OR all("blackflies") | 27,879 |
| **10** | all("rabies") OR all("schistosomiasis") OR all("parasitic worms") OR all("blood flukes") OR all("trematode worms") OR all("Intestinal schistosomiasis") OR all("schistosoma mansoni") OR all("schistosoma japonicum") OR all("schistosoma mekongi") | 25,209 |
| **11** | all("schistosoma guineensis") OR all("schistosoma intercalatum") OR all("urogenital schistosomiasis") OR all("schistosoma haematobium") OR all("soil-transmitted helminthiases") OR all("soil-transmitted helminth infection") OR all("roundworm") OR all("ascaris lumbricoides") OR all("whipworm") OR all("trichuris trichiura") | 4,914 |
| **12** | all("hookworms") OR all("necator americanus") OR all("ancylostoma duodenale") OR all(taeniasis/cysticercosis) OR all(taeniasis) OR all(cysticercosis) OR all(tapeworms) OR all("taenia solium") OR all("pork tapeworm") OR all("taenia saginata") | 7,269 |
| **13** | all("beef tapeworm") OR all("taenia asiatica") OR all("cysticerci") OR all("trachoma") OR all("chlamydia trachomatis") OR all("endemic treponematoses") OR all("yaws") OR all("treponema pallidum") OR all("pertenue") | 15,938 |
| **14** | 2 OR 3 OR 4 OR 5 OR 6 OR 7 OR 8 OR 9 OR 10 OR 11 OR 12 OR 13 | 174,796 |
| **15** | all(intervention) OR all(campaign) OR all(strategy) OR all(program) OR all(project) | 2,465,516 |
| **15** | #1 AND #14 AND #15 | 329 |

**Web of Science (WOS)**

All databases

Timespan=1971-2017

Search language=Auto

| **#** | **Searches** | **Results** |
| --- | --- | --- |
| **1** | TOPIC: ("buruli ulcer") OR TOPIC: ("mycobacterium ulcerans") OR TOPIC: ("chagas disease") OR TOPIC: ("american trypanosomiasis") OR TOPIC: ("trypansosoma cruzi") OR TOPIC: ("dengue") OR TOPIC: ("mosquito") OR TOPIC: ("chikungunya") OR TOPIC: ("dracunculiasis") OR TOPIC: ("guinea-worm disease") OR TOPIC: ("dracunculus medinensis") OR TOPIC: ("echinococcosis") OR TOPIC: ("echinococcus granulosus") OR TOPIC: ("cystic echinococcosis") OR TOPIC: (hydatidosis) OR TOPIC: ("hydatid disease") OR TOPIC: ("alveolar echinococcosis") OR TOPIC: ("echinococcus multilocularis") OR TOPIC: ("polycystic echinococcosis") OR TOPIC: ("echinococcus vogeli") OR TOPIC: ("unicystic echinococcosis") OR TOPIC: ("echinococcus oligarthrus") OR TOPIC: ("foodborne trematodiases") OR TOPIC: ("trematode worms") OR TOPIC: ("flukes") | 113,353 |
| **2** | TOPIC: ("freshwater snail") OR TOPIC: ("freshwater fish") OR TOPIC: ("clonorchis sinensis") OR TOPIC: ("opisthorchis viverrini") OR TOPIC: ("opisthorchis Felineus") OR TOPIC: ("fasciola hepatica") OR TOPIC: ("fasciola gigantica") OR TOPIC: ("paragonimus") OR TOPIC: ("human african trypanosomiasis") OR TOPIC: ("sleeping sickness") OR TOPIC: ("trypanosoma brucei gambiense") OR TOPIC: ("tsetse flies") OR TOPIC: ("glossina genus") OR TOPIC: ("trypanosoma brucei rhodesiense") OR TOPIC: (nagana) OR TOPIC: (leishmaniasis) OR TOPIC: ("visceral leishmaniasis") OR TOPIC: ("cutaneous leishmaniasis") OR TOPIC: ("mucocutaneous leishmaniasis") OR TOPIC: (kala-azar) OR TOPIC: ("leishmania parasites") OR TOPIC: ("phlebotomine sandflies") OR TOPIC: ("leprosy") OR TOPIC: ("hansen's disease") OR TOPIC: ("mycobacterium leprae") | 87,557 |
| **3** | TITLE: ("freshwater snail") OR TITLE: ("freshwater fish") OR TITLE: ("clonorchis sinensis") OR TITLE: ("opisthorchis viverrini") OR TITLE: ("opisthorchis Felineus") OR TITLE: ("fasciola hepatica") OR TITLE: ("fasciola gigantica") OR TITLE: ("paragonimus") OR TITLE: ("human african trypanosomiasis") OR TITLE: ("sleeping sickness") OR TITLE: ("trypanosoma brucei gambiense") OR TITLE: ("tsetse flies") OR TITLE: ("glossina genus") OR TITLE: ("trypanosoma brucei rhodesiense") OR TITLE: (nagana) OR TITLE: (leishmaniasis) OR TITLE: ("visceral leishmaniasis") OR TITLE: ("cutaneous leishmaniasis") OR TITLE: ("mucocutaneous leishmaniasis") OR TITLE: (kala-azar) OR TITLE: ("leishmania parasites") OR TITLE: ("phlebotomine sandflies") OR TITLE: ("leprosy") OR TITLE: ("hansen's disease") OR TITLE: ("mycobacterium leprae") | 42,273 |
| **4** | TI=("buruli ulcer") OR TI=("mycobacterium ulcerans") OR TI=("chagas disease") OR TI=("american trypanosomiasis") OR TI=("trypansosoma cruzi") OR TI=("dengue") OR TI=("mosquito") OR TI=("chikungunya") OR TI=("dracunculiasis") OR TI=("guinea-worm disease") OR TI=("dracunculus medinensis") OR TI=("echinococcosis") OR TI=("echinococcus granulosus") OR TI=("cystic echinococcosis") OR TI=(hydatidosis) OR TI=("hydatid disease") OR TI=("alveolar echinococcosis") OR TI=("echinococcus multilocularis") OR TI=("polycystic echinococcosis") OR TI=("echinococcus vogeli") OR TI=("unicystic echinococcosis") OR TI=("echinococcus oligarthrus") OR TI=("foodborne trematodiases") OR TI=("trematode worms") OR TI=("flukes") | 46,052 |
| **5** | TOPIC: ("lymphatic filariasis") OR TOPIC: (elephantiasis) OR TOPIC: (nematodes) OR TOPIC: (roundworms) OR TOPIC: ("wuchereria bancrofti") OR TOPIC: ("brugia malayi") OR TOPIC: ("brugia timori") OR TOPIC: (culex) OR TOPIC: (anopheles) OR TOPIC: (aedes) OR TOPIC: (onchocerciasis) OR TOPIC: ("river blindness") OR TOPIC: ("onchocerca volvulus") OR TOPIC: (simulium) OR TOPIC: (blackflies) OR TOPIC: (rabies) OR TOPIC: (schistosomiasis) OR TOPIC: ("parasitic worms") OR TOPIC: ("blood flukes") OR TOPIC: ("trematode worms") OR TOPIC: ("Intestinal schistosomiasis") OR TOPIC: ("schistosoma mansoni") OR TOPIC: ("schistosoma japonicum") OR TOPIC: ("schistosoma mekongi") OR TOPIC: ("schistosoma guineensis") | 258,919 |
| **6** | TITLE: ("lymphatic filariasis") OR TITLE: (elephantiasis) OR TITLE: (nematodes) OR TITLE: (roundworms) OR TITLE: ("wuchereria bancrofti") OR TITLE: ("brugia malayi") OR TITLE: ("brugia timori") OR TITLE: (culex) OR TITLE: (anopheles) OR TITLE: (aedes) OR TITLE: (onchocerciasis) OR TITLE: ("river blindness") OR TITLE: ("onchocerca volvulus") OR TITLE: (simulium) OR TITLE: (blackflies) OR TITLE: (rabies) OR TITLE: (schistosomiasis) OR TITLE: ("parasitic worms") OR TITLE: ("blood flukes") OR TITLE: ("trematode worms") OR TITLE: ("Intestinal schistosomiasis") OR TITLE: ("schistosoma mansoni") OR TITLE: ("schistosoma japonicum") OR TITLE: ("schistosoma mekongi") OR TITLE: ("schistosoma guineensis") | 85,801 |
| **7** | TOPIC: ("schistosoma intercalatum") OR TOPIC: ("urogenital schistosomiasis") OR TOPIC: ("schistosoma haematobium") OR TOPIC: ("soil-transmitted helminthiases") OR TOPIC: ("soil-transmitted helminth infection") OR TOPIC: (roundworm) OR TOPIC: ("ascaris lumbricoides") OR TOPIC: (whipworm) OR TOPIC: ("trichuris trichiura") OR TOPIC: (hookworms) OR TOPIC: ("necator americanus") OR TOPIC: ("ancylostoma duodenale") OR TOPIC: ("taeniasis/cysticercosis") OR TOPIC: (taeniasis) OR TOPIC: (cysticercosis) OR TOPIC: (tapeworms) OR TOPIC: ("taenia solium") OR TOPIC: ("pork tapeworm") OR TOPIC: ("taenia saginata") OR TOPIC: ("beef tapeworm") OR TOPIC: ("taenia asiatica") OR TOPIC: (cysticerci) OR TOPIC: (trachoma) OR TOPIC: ("chlamydia trachomatis") | 53,915 |
| **8** | TITLE: ("schistosoma intercalatum") OR TITLE: ("urogenital schistosomiasis") OR TITLE: ("schistosoma haematobium") OR TITLE: ("soil-transmitted helminthiases") OR TITLE: ("soil-transmitted helminth infection") OR TITLE: (roundworm) OR TITLE: ("ascaris lumbricoides") OR TITLE: (whipworm) OR TITLE: ("trichuris trichiura") OR TITLE: (hookworms) OR TITLE: ("necator americanus") OR TITLE: ("ancylostoma duodenale") OR TITLE: ("taeniasis/cysticercosis") OR TITLE: (taeniasis) OR TITLE: (cysticercosis) OR TITLE: (tapeworms) OR TITLE: ("taenia solium") OR TITLE: ("pork tapeworm") OR TITLE: ("taenia saginata") OR TITLE: ("beef tapeworm") OR TITLE: ("taenia asiatica") OR TITLE: (cysticerci) OR TITLE: (trachoma) OR TITLE: ("chlamydia trachomatis") | 20,129 |
| **9** | TOPIC: ("endemic treponematoses") OR TITLE: ("endemic treponematoses") OR TOPIC: (yaws) OR TITLE: (yaws) OR TOPIC: ("treponema pallidum") OR TITLE: ("treponema pallidum") OR TOPIC: (pertenue) OR TITLE: (pertenue) | 17,935 |
| **10** | TOPIC: ("neglected tropical disease") OR TITLE: ("neglected tropical disease") OR TOPIC: ("neglected disease") OR TITLE: ("neglected disease") OR TOPIC: ("tropical disease") OR TITLE: ("tropical disease") OR TOPIC: (NTD) OR TITLE: (NTD) | 6,169 |
| **11** | #10 OR #9 OR #8 OR #7 OR #6 OR #5 OR #4 OR #3 OR #2 OR #1 | 453,644 |
| **12** | TOPIC: ("social marketing") OR TITLE: ("social marketing") OR TOPIC: ("health communication") OR TITLE: ("health communication") OR TOPIC: ("health promotion") OR TITLE: ("health promotion") | 99,458 |
| **14** | TOPIC: (intervention) OR TOPIC: (campaign) OR TOPIC: (project) OR TOPIC: (program) OR TOPIC: (strategy) OR TITLE: (intervention) OR TITLE: (campaign) OR TITLE: (project) OR TITLE: (program) OR TITLE: (strategy) | 4,724,095 |
| **15** | 11 AND 12 AND 13 | 662 |

**Global Index Medicus**

**Regional Indexes** AIM (AFRO), LILACS (AMRO/PAHO), IMEMR (EMRO), IMSEAR (SEARO), WPRIM (WPRO)

**Global Index** Regional Indexes, MEDLINE, SciELO

**Institutional Repository** WHO IRIS

**Results:** 932

(tw:((tw:("social marketing")) OR (tw:("health promotion")) OR (tw:("health communication")) AND (tw:("neglected disease")) OR (tw:("neglected tropical disease")) OR (tw:("tropical disease")) OR (tw:("NTD")) OR (tw:(buruli ulcer)) OR (tw:(mycobacterium ulcerans)) OR (tw:(chagas disease)) OR (tw:(american trypanosomiasis)) OR (tw:(trypansosoma cruzi)) OR (tw:(dengue)) OR (tw:(mosquito)) OR (tw:(chikungunya)) OR (tw:(dracunculiasis)) OR (tw:(guinea-worm disease)) OR (tw:(dracunculus medinensis)) OR (tw:(echinococcosis)) OR (tw:(echinococcus granulosus)) OR (tw:(cystic echinococcosis)) OR (tw:(hydatidosis)) OR (tw:(hydatid disease)) OR (tw:(alveolar echinococcosis)) OR (tw:(echinococcus multilocularis)) OR (tw:(polycystic echinococcosis)) OR (tw:(echinococcus vogeli)) OR (tw:(unicystic echinococcosis)) OR (tw:(echinococcus oligarthrus)) OR (tw:(foodborne trematodiases)) OR (tw:(trematode worms )) OR (tw:(flukes)) OR (tw:(freshwater snail)) OR (tw:(freshwater fish)) OR (tw:(clonorchis sinensis)) OR (tw:(opisthorchis viverrini)) OR (tw:(opisthorchis felineus)) OR (tw:(fasciola hepatica)) OR (tw:(fasciola gigantica)) OR (tw:(paragonimus)) OR (tw:(human african trypanosomiasis)) OR (tw:(sleeping sickness)) OR (tw:(trypanosoma brucei gambiense)) OR (tw:(tsetse flies)) OR (tw:(glossina genus)) OR (tw:(trypanosoma brucei rhodesiense)) OR (tw:(nagana)) OR (tw:(leishmaniasis)) OR (tw:(visceral leishmaniasis )) OR (tw:(cutaneous leishmaniasis)) OR (tw:(mucocutaneous leishmaniasis)) OR (tw:(kala-azar)) OR (tw:(leishmania parasites)) OR (tw:(phlebotomine sandflies)) OR (tw:(leprosy)) OR (tw:(hansen's disease)) OR (tw:(mycobacterium leprae)) OR (tw:(lymphatic filariasis)) OR (tw:(elephantiasis)) OR (tw:(nematodes)) OR (tw:(roundworms)) OR (tw:(wuchereria bancrofti)) OR (tw:(brugia malayi)) OR (tw:(brugia timori)) OR (tw:(culex)) OR (tw:(anopheles)) OR (tw:(aedes)) OR (tw:(onchocerciasis)) OR (tw:(river blindness)) OR (tw:(onchocerca volvulus)) OR (tw:(simulium)) OR (tw:(blackflies )) OR (tw:(rabies)) OR (tw:(schistosomiasis)) OR (tw:(parasitic worms)) OR (tw:(blood flukes)) OR (tw:(trematode worms)) OR (tw:(intestinal schistosomiasis)) OR (tw:(schistosoma mansoni)) OR (tw:(schistosoma japonicum)) OR (tw:(schistosoma mekongi)) OR (tw:(schistosoma guineensis)) OR (tw:(schistosoma intercalatum)) OR (tw:(urogenital schistosomiasis)) OR (tw:(schistosoma haematobium)) OR (tw:(soil-transmitted helminthiases)) OR (tw:(soil-transmitted helminth infection)) OR (tw:(roundworm )) OR (tw:(ascaris lumbricoides)) OR (tw:(whipworm)) OR (tw:(trichuris trichiura)) OR (tw:(hookworms)) OR (tw:(necator americanus)) OR (tw:(ancylostoma duodenale)) OR (tw:(taeniasis/cysticercosis)) OR (tw:(taeniasis)) OR (tw:(cysticercosis)) OR (tw:(tapeworms)) OR (tw:(taenia solium)) OR (tw:(pork tapeworm)) OR (tw:(taenia saginata)) OR (tw:(beef tapeworm)) OR (tw:(taenia asiatica)) OR (tw:(cysticerci)) OR (tw:(trachoma)) OR (tw:(chlamydia trachomatis)) OR (tw:(endemic treponematoses)) OR (tw:(yaws)) OR (tw:(treponema pallidum)) OR (tw:(pertenue)))) AND (tw:((intervention) OR (tw:(strategy)) OR (tw:(program)) OR (tw:(campaign)) OR (tw:(project))))

**Virtual Health Library (VHL) Regional Portal**

**Results:** 975

(tw:((tw:("social marketing")) OR (tw:("health promotion")) OR (tw:("health communication")) AND (tw:("neglected disease")) OR (tw:("neglected tropical disease")) OR (tw:("tropical disease")) OR (tw:("NTD")) OR (tw:(buruli ulcer)) OR (tw:(mycobacterium ulcerans)) OR (tw:(chagas disease)) OR (tw:(american trypanosomiasis)) OR (tw:(trypansosoma cruzi)) OR (tw:(dengue)) OR (tw:(mosquito)) OR (tw:(chikungunya)) OR (tw:(dracunculiasis)) OR (tw:(guinea-worm disease)) OR (tw:(dracunculus medinensis)) OR (tw:(echinococcosis)) OR (tw:(echinococcus granulosus)) OR (tw:(cystic echinococcosis)) OR (tw:(hydatidosis)) OR (tw:(hydatid disease)) OR (tw:(alveolar echinococcosis)) OR (tw:(echinococcus multilocularis)) OR (tw:(polycystic echinococcosis)) OR (tw:(echinococcus vogeli)) OR (tw:(unicystic echinococcosis)) OR (tw:(echinococcus oligarthrus)) OR (tw:(foodborne trematodiases)) OR (tw:(trematode worms )) OR (tw:(flukes)) OR (tw:(freshwater snail)) OR (tw:(freshwater fish)) OR (tw:(clonorchis sinensis)) OR (tw:(opisthorchis viverrini)) OR (tw:(opisthorchis felineus)) OR (tw:(fasciola hepatica)) OR (tw:(fasciola gigantica)) OR (tw:(paragonimus)) OR (tw:(human african trypanosomiasis)) OR (tw:(sleeping sickness)) OR (tw:(trypanosoma brucei gambiense)) OR (tw:(tsetse flies)) OR (tw:(glossina genus)) OR (tw:(trypanosoma brucei rhodesiense)) OR (tw:(nagana)) OR (tw:(leishmaniasis)) OR (tw:(visceral leishmaniasis )) OR (tw:(cutaneous leishmaniasis)) OR (tw:(mucocutaneous leishmaniasis)) OR (tw:(kala-azar)) OR (tw:(leishmania parasites)) OR (tw:(phlebotomine sandflies)) OR (tw:(leprosy)) OR (tw:(hansen's disease)) OR (tw:(mycobacterium leprae)) OR (tw:(lymphatic filariasis)) OR (tw:(elephantiasis)) OR (tw:(nematodes)) OR (tw:(roundworms)) OR (tw:(wuchereria bancrofti)) OR (tw:(brugia malayi)) OR (tw:(brugia timori)) OR (tw:(culex)) OR (tw:(anopheles)) OR (tw:(aedes)) OR (tw:(onchocerciasis)) OR (tw:(river blindness)) OR (tw:(onchocerca volvulus)) OR (tw:(simulium)) OR (tw:(blackflies )) OR (tw:(rabies)) OR (tw:(schistosomiasis)) OR (tw:(parasitic worms)) OR (tw:(blood flukes)) OR (tw:(trematode worms)) OR (tw:(intestinal schistosomiasis)) OR (tw:(schistosoma mansoni)) OR (tw:(schistosoma japonicum)) OR (tw:(schistosoma mekongi)) OR (tw:(schistosoma guineensis)) OR (tw:(schistosoma intercalatum)) OR (tw:(urogenital schistosomiasis)) OR (tw:(schistosoma haematobium)) OR (tw:(soil-transmitted helminthiases)) OR (tw:(soil-transmitted helminth infection)) OR (tw:(roundworm )) OR (tw:(ascaris lumbricoides)) OR (tw:(whipworm)) OR (tw:(trichuris trichiura)) OR (tw:(hookworms)) OR (tw:(necator americanus)) OR (tw:(ancylostoma duodenale)) OR (tw:(taeniasis/cysticercosis)) OR (tw:(taeniasis)) OR (tw:(cysticercosis)) OR (tw:(tapeworms)) OR (tw:(taenia solium)) OR (tw:(pork tapeworm)) OR (tw:(taenia saginata)) OR (tw:(beef tapeworm)) OR (tw:(taenia asiatica)) OR (tw:(cysticerci)) OR (tw:(trachoma)) OR (tw:(chlamydia trachomatis)) OR (tw:(endemic treponematoses)) OR (tw:(yaws)) OR (tw:(treponema pallidum)) OR (tw:(pertenue)))) AND (tw:((intervention) OR (tw:(strategy)) OR (tw:(program)) OR (tw:(campaign)) OR (tw:(project))))
