# Supplementary material for: Social marketing interventions for the prevention and control of neglected tropical diseases: A systematic review
Source: PLoS Negl Trop Dis. 2020 Jun 17;14(6):e0008360. doi: 10.1371/journal.pntd.0008360 (PMC7299328; doi:10.1371/journal.pntd.0008360)
Supplement: S2 File — (DOCX) [file pntd.0008360.s002.docx]

**S2 File. Summary of the interventions.**

| **1. General Information** | **2. Basic Characteristics** | **3. Intervention** | **4. Social Marketing Characteristics** | |
| --- | --- | --- | --- | --- |
| **Intervention ID**: I1-GUI  **Author, year:**  Brieger et al., 1989; Brieger et al., 1986; Brieger et al., 1990; Adeniyi and Brieger, 1983  **Year of implementation**: 1985-1986  **Location:** Idere, Nigeria, Africa, Lower middle income | **NTD**: Guinea-worm disease (Dracunliasis)  **Targeted stream:** Midstream  **Public:**  - Communities.  - 27 PHWs involved as co-designers and co-implementers.  **Setting:** The rural community was divided in 3 sectors: (1) Main town, (2) farm hamlets located northeast of town and (3) farm hamlets located west of town.  **Sample size:** Pre-intervention survey: 371 residents.  Post-intervention: 779 households. | **Aim:** To prompt the communities to buy and use a monofilament nylon cloth filter to prevent Guinea-worm.  **Behavior:** Purchase and use of monofilament nylon cloth filter for water.  **Description:**  - 15-month intervention that included: baseline survey; technical development, production, and sales-distribution (6-month period) of a monofilament nylon water filter; training for sales people with a focus on health education; monitoring and a follow-up survey.  - Volunteer primary health workers members of the PHW Association actively participated in intervention design and delivery upon invitation from the research team. They engaged the Idere Young Tailors Association to produce the filters and the King of Idere to support in promotion. They also sold the filters and in the selling process educated community members on the correct use of the water filters (10-step process).  - A field assistant participated in intervention delivery and was responsible of monitoring. Follow-up survey by medical students.  **WHO NTD strategy:** Water, sanitation and hygiene | **Concepts:** **1) Social Behavioral influence**:  - Purchase and regular use of a monofilament nylon cloth filter.  - 10 steps for the correct use of the filter: (1) Place filter on pop with black thread facing upwards, (2) Allow filter to sag slightly so water will not splash, (3) Pour water slowly, (4) Let all the water drain out, (5) Remove filter carefully to prevent cyclops falling in, (6) Shake the filter after each use, (7) Wash the filter after use, (8) Hang the filter outside to dry, (9) Store in safe place to avoid sharp objects, (10) Inspect filter before use for holes and tears.  **2) Public / people orientation:**  - Previous experience of the research team working with the community and the volunteer primary health workers (PHWs) informed the intervention focus (short term: filters, long term post intervention: wells through fundraising) and facilitated involvement of PHWs.  - Before the start of the intervention, a TDR consultant developed the product prototype, tested by the research assistant. The TDR provided a small quantity of the monofilament cloth.  - Baseline survey of potential consumers informed product design and promotion. To determine potential acceptability of the product, monofilament filters where shown and appropriate pricing ideas collected from survey participants  **3) Social offerings**:  - Purchase and use of monofilament nylon water filters available in sizes and prices. Filters were sold by volunteer PHWs, local traders and the project research assistant, who provided health education on the correct and regular use of the filter (10 steps).  **4) Relationship building**:  - The TDR assumed the costs of staff time, transport, promotion and supervision of distribution and sales, aiming at offering the filters at a reasonable price to not inhibit purchase.  - Through the PHW Association, PHWs participated in: Filter design and production, identification/engagement of local tailors (Idere Young Tailors Association) to produce the filters, setting a fair price, distribution and health education, and filter promotion. PHW Association had a high sense of ownership to the project as selling filters helped them reach the goals of their Association.  - The PHWs Association engaged the King of Idere in product promotion. | **Techniques:** **1) Integrated intervention mix**:  - The filter was a microfilament cloth cut in circles and a rubber band (commonly sold in the market) sewn into the edges. It was produced in 3 sizes: small (diameter: less than 13 inches, 1,25 USD), medium (13-16 inches, 1,50 USD), and large (>16 inches, 2,5 USD).  - 10 steps were developed to educate about the safe and correct way to use the filter.  - The PHWs deliberated and agreed that a fixed profit of 20 cents per filter should be fixed to incentivize the salesforce.  - Profits from the sales were contributed by the intervention to a community well fund.  - Salesforce: 35 people (27 PHWs, 7 townspeople (4 women selling in the local markets, 1 tailor, 1 shoemaker and 1 famer/preacher)) and 1 project field assistant.  - Health education training to salespeople about the purpose and correct use of the filters, using demonstration. They were instructed that health education was their main task.  - PHW Association responsible for community awareness and promoting sales.  - Village and compound meetings and house-to-house visits to explain and demonstrate the use of the filters, announcements at churches and mosques.  - The King of Idere was reached by the PHWs and agreed to help in promotion by having the town criers announce the filters.  - Field assistant promoted sales and educated about the regular and correct use of the filters  Partnership with PHW Association involved in all aspects of the intervention as co-creators and with the Young Tailors Association in product production.  **2) Competition analysis and action**:  - Intervention addressed: (i) preference for wells instead of filters, (ii) preference to use alum, (iii) perception of low efficacy of water filters.  **3) Systematic planning and evaluation**:  - Prototype development based on study of the local drinking pots and pond water to determine cyclops species, and included visiting tailors and the market.  - Monitoring helped to test the usability of the product and to reinforce health education.  - The intervention team and PHW leaders monitored filter production quality.  - Research assistant visited each hamlet and a sample of family compounds of sector 1. The selected town compounds were those where a PHW was resident and an equal number without PHW as resident. A total of 30 compounds out of 75 in sector 1 were monitored.  - Monthly monitoring form consisting in two parts: (1) To report the current filter ownership status per home and demographic information, and (2) To record the condition of the filter, reported use, opinions and problems from filter use, on each visit post filter acquisition.  - Use monitored from the moment the family bought the filter (from 1 to 5 months).  - October 1986, follow up survey conducted by medical students in a cluster comprising 16 hamlets to: determine use, retained knowledge of proper use, and see filter condition.  **4) Insight-driven segmentation**:  - Baseline survey findings informed the development of a 10-step process for correct filter use.  - Cost of local production determined the selection of monofilament nylon filters instead of wooden framed filters  - Salespeople allocation to sectors considered the usual agenda of the PHWs.  **5) Co-creation**:  - Young Tailors Association used their own methods to produce filters that were feasible to use in the local context.  - One PHW had extra supplies of filers in her house.  - The health education team brought filters at every PHWs meeting.  - Receipts were handled by the PHWs to the health education team at the PHWs meeting. The field assistant collected receipts from non-PHWs and from PHWs not able to attend the meetings. |
| **Intervention ID**: I2-CYS  **Author, year:**  Dickey et al., 2015; Dickey et al., 2016; Dickey, 2014  **Year of implementation**: 2011 - 2013  **Location:**  Eryuan County, Yunnan, China, Western Pacific, upper middle income | **NTD**: Cysticercosis  **Targeted stream:** Midstream  **Public:**  Communities of Bai people (minority ethnic group)  **Setting:**  2 intervention villages, 2 comparison villages  **Sample size:**  NA | **Aim:** Increase household toilet building and use for cysticercosis reduction.  **Behavior:** Household toilet building and use.  **Description:**  - 18 months intervention  - Construction of three-chamber toilets accompanied with laminated instructions about flushing and water use.  - Project and government subsidies for each household given after toilet quality inspection.  - Toilets placed according to preference from homeowners.  - Homeowners responsible for building local materials and for construction.  - Local project building supervisor promoted and inspected the toilets, and trained local builders. He received monetary incentive per toilet built.  - Demonstration toilets, public kick-off event, door-to-door and word-of-mouth promotion.  - Promotion material: brochures with pictures for the illiterate, posters with pictures of households with demonstration toilets built, and branded baseball caps.  **WHO NTD strategy:**  Water, sanitation and hygiene | **Concepts:** **1) Social Behavioral influence**:  - Household toilet building and use.  **2) Public / people orientation:**  The formative research consisted on:  - Baseline door-to-door survey of sanitation practices  - Baseline human prevalence of NCC study. If positive results, free antigen-detecting and antibody-detecting ELISA serological testing was offered. If these were positive too, a free computerized tomography scan of the brain was provided.  - Baseline porcine prevalence study  - Rural participatory assessment tool (ten-seed technique)  - Gender-specific focus group discussions  - Key informant interviews, participant observation, and a rapid ethnographic assessment.  - Focus group questions piloted.  - Findings from formative research informed content of promotion material focusing on convenience, privacy, cleanliness, avoidance of embarrassment and progress.  **3) Social offerings**:  - Household toilets meeting sanitary quality standards and the preferences of homeowners, who participated in the selection, placement and construction processes.  **4) Relationship building**:  - 3-year Memorandum of Understanding signed by the National Institute for Parasitic Diseases (NIPD), the Dali Institute for Parasitic Diseases, the Eryuan Station for Parasitic Diseases, and the research project coordinator.  - Bai volunteers from the Dali Health College supported in formative research.  - The research team was involved in actions in the field which facilitated building relationships with community members.  - Government officials engaged and expressed public support to the project.  - Local toilet building supervisor trained local builders and promoted toilet building in the communities. | **Techniques:** **1) Integrated intervention mix**:  - Three-chamber mini-septic tank system toilet accompanied with laminated instructions about flushing and water use.  - Three-chamber toilet construction training to local men using local materials  Price:  - Subsidies for each household: 400 yuan (US$65) by the project, 600 yuan (US$98) by the government. Subsidies given at the end after toilet quality inspection.  - Toilets placed according to preference from homeowners, ensuring sanitary criteria and agreement with neighbors.  - Local project building supervisor promoted and inspected the toilets, and trained local builders. He was paid US$65 for each toilet built as an incentive.  - Demonstration toilets (2 per intervention village, one simple and one nicer looking)  - Public kick-off event at an elementary school, followed by a fair with games and prizes.  - Door-to-door and word-of-mouth promotion  - Promotion material: brochures with pictures for the illiterate, posters with pictures of households with demonstration toilets built, and branded baseball caps  **2) Competition analysis and action**: NA  **3) Systematic planning and evaluation**:  - Process (logic models, standards of procedure SOPs, calendar), outcome and impact evaluation.  - The sustainability and reproducibility of the actions post campaign were considered.  - Baseline human neurocysticercosis and porcine cysticercosis prevalence studies.  - Follow-up toilet satisfaction evaluations three months after a toilet was built, both in the intervention and comparison villages.  - Post-intervention household survey in the 4 villages.  **4) Insight-driven segmentation**:  - Tailored toilet selection and placement.  **5) Co-creation**:  - Homeowners decided where to place toilets and where in charge of the building materials and construction. |
| **Intervention ID**: I3-DEN  **Author, year:**  Caprara et al., 2015; Alfonso-Sierra et al., 2016   **Year of implementation**: 2012 - 2013  **Location:** Fortaleza, Brazil, Americas, upper middle income | **NTD**: Dengue  **Targeted stream:** Midstream  **Public:**  Communities  **Setting:**  Study design: Cluster randomized controlled trial, 10 intervention clusters, 10 control clusters (where routine vector control activities were implemented).  **Sample size:**  Entomological surveys in 2411 places (2353 households, 58 public spaces). | **Aim:** Reduce *Aedes aegypti* vector density by controlling productive container types and discarded containers through an ecohealth approach, involving the community and improving the relationship with the Endemic Disease Agents (EDAs) who visit the houses.  **Behavior:** Reduce small discarded and unused water containers stored in backyards and public spaces and cover the elevated containers and in-house rubbish disposal without larvaciding.  **Description:**  - Community workshops.  - Clean-up campaigns with community involvement.  - Covering the elevated containers and in-house rubbish disposal without larvaciding.  - Partnerships with schools and public control services.  - Mobilization of schoolchildren, senior inhabitants and Endemic Disease Agents (EDAs).  - Distribution of information materials.  **WHO NTD strategy:**  Vector ecology and management. | **Concepts:** **1) Social Behavioral influence**:  Reduce small discarded and unused water containers stored in backyards and public spaces and cover the elevated containers and in-house rubbish disposal without larvicide.  **2) Public / people orientation:**  - Situational analysis to characterize and map the urban ecosystem. This informed the container focus (water tanks on the roof/ground, small discarded containers).  - Entomological surveys, use of participatory research methods, key informant interviews and participant observations informed intervention design and implementation.  **3) Social offerings**:  - A participatory approach for adopting routine vector control activities without using larvicides.  **4) Relationship building**:  - Key stakeholders engaged since the onset: community members (C ), community leaders (L), professionals related to the municipal endemic diseases control program (E ), professionals working at the health centers (S).  - Meetings with intersectorial groups to explain objectives and procedures of the activities in the homes.  - Waste collection truck requested to the Regional Secretariat.  - Active community involvement in organizing meetings and participating in clean-up campaigns.  - Partnerships with schools focusing on dengue control and solid waste management and with public control services.  - Educational calendars co-developed by research team and the health authorities.  - Social mobilization groups formed by National Health Service professionals, educators and Endemic Disease Agents (EDAs).  - Community groups (elder's groups, students, community members) were mobilized and empowered.  - Some clusters organized garbage collection with their Regional Secretariats and communities. | **Techniques:** **1) Integrated intervention mix**:  - Garbage bags, lids and covers for large water tanks provided  - Environmental management activities (removing discarded small recipients, covering large water containers without using larvicides or insecticides)  - Clean-up campaigns with community members and EDAs to clean backyard areas and public spaces.  - Workshops and community meetings.  - EDAs visited homes, delivered garbage bags, informed date of garbage collection by the garbage truck, provided general information.  - Elderly people distributed information material (e.g. leaflets).  - Educational calendar to remind dengue prevention actions.  **2) Competition analysis and action**: NA  **3) Systematic planning and evaluation**:  - Pre-intervention entomological survey (November-December 2012).  - Intervention developed in the rainy season (January - April 2013).  - Post-intervention entomological survey (May 2013).  **4) Insight-driven segmentation**: NA  **5) Co-creation**:  - Workshops with professionals from the Family Health Strategy and the Department of Endemic Disease Control, Social Educators, community leaders, and community members to discuss results of the situational analysis and planned actions considering the needs of each locality. |
| **Intervention ID**: I4-LYM  **Author, year:**  Ramaiah et al., 2006  **Year of implementation**: 2002  **Location:** Tamil Nadu State, India, South-East Asia, lower middle income | **NTD**: Lymphatic filariasis    **Targeted stream:**  Midstream / upstream  **Public:**  General population  State, district and village level administrations  **Setting:**  *- Baseline*: 18 villages in Cudalore district, 18 villages in Villapuram district.  *- Intervention:* 12 districts.  *- Evaluation:* 36 villages and 12 urban areas from 4 districts.  **Sample size:**  Pre-intervention baseline 875 households/4.182 people; post-intervention evaluation 792 households/3.862 people. | **Aim:** To motivate the population to participate and take treatment (DEC, DEC-albendazole) offered via MDA on Filaria Day.  **Behavior:** Accept and consume treatment (DEC, DEC-albendazole) offered via MDA.  **Description:**  - Intervention with advocacy and public relations at the state, district and village level administrations, social mobilization and communication via multiple media to promote compliance with MDA. The intervention had 2 variants: (1) COMBI(+) in 6 villages with DEC MDA funded by the State; (2) COMBI(++) in 6 villages with DEC-albendazole MDA funded by WHO.  **WHO NTD strategy:**  Preventive chemotherapy and transmission control (PCT) | **Concepts:** **1) Social Behavioral influence**:  - Receive and ingest DEC/DEC-albendazole tablets distributed during MDA on Filaria Day.  **2) Public / people orientation:**  - Observations in villages and a workshop informed the intervention design.  - Baseline research findings informed the design of the intervention, this included suggestions to improve compliance with treatment (e.g. have more people involved, selection of communication channels).  **3) Social offerings**:  - Multiple advocacy/PR, mobilization and communication channels used to engage key stakeholders and prompt communities to participate and take treatment offered during the Filaria Day MDA.  **4) Relationship building**:  - State level activities: (1) meeting with the chief minister of Tamil Nadu this resulted in a press release from him asking people's support with the MDA. (2) engagement of state-level heads of various departments. (3) Workshop with directors and deputy directors of various departments.  - District level activities: (1) Press releases from the chief of the district administration. (2) Meeting of the chief of the district administration with heads of several departments.  - Village level activities: (1) Meeting of health workers with the president and members of each village body of of elected representatives (panchayat), they were asked to encourage community participation. (2) Explanation of the program to villagers by health workers from the relevant PHC.  - Engagement of school teachers by health workers, they were invited to organize student rallies. | **Techniques:** **1) Integrated intervention mix**:  - 2 types of interventions: (1) MDA with DEC and COMBI(+); (2) MDA with DEC-albendazole and COMBI(++).  - Free treatment. Tablets offered on Filaria Day  - 3 Door-to-door visits per household by trained Filaria Prevention Assistants (FPA). 1st visit to collect basic information and build rapport, 2nd visit to provide LF education and show disease recognition card, third visit on Filaria Day to give treatment.  - FPA were wearing branded badge and fabric bags.  - FPA received a one-time honorarium (30-100 Indian Ruppies, US 0.67-2.22)  - Two logos, one with the date of the drug distribution and the other of the adult dose of DEC.  - Touring bicycle teams of 8-12 people wearing Filaria Day t-shirts drove around the villages.  - Groups of 5-8 danglers with the logo hung in strategic places around the villages.  - Posters one with State endorsement by the Chief Minister and other with the campaign message.  - Ribbon flags and pamphlets distributed.  - Registration slips delivered to households. Householders complying with treatment could participate in a lottery and win prizes.  - Advertising campaign 15 days prior Filaria Day via radio, TV and newspaper. Loudspeaker announcements used in rural areas. Cinema presentations in urban and rural areas.  - Schoolchildren received flyers, read them to their parents and returned a tear-off slip signed. - State level meeting of district health officers where they received information about the intervention and were trained in role-playing to explain how the program could be improved to PHC-level health staff.  - District medical officer convened medical officers of all the PHCs in the district, role playing used to explain how to improve distribution and compliance with treatment.  - At the PHC level, medical officers trained junior colleagues on LF transmission and MDA.  **2) Competition analysis and action**: NA  **3) Systematic planning and evaluation**:  - Strategic engagement and involvement of key actors using multiple methods.  - FPAs personal engagement with householders strategically planned in 3 moments.  - Baseline: 2 districts. Focus group discussions and semi-structured interviews on attitudes towards the MDA and factors influencing compliance with treatment. Household survey in 40 communities (village and urban wards, on knowledge of LF and exposure to media.  - Post-intervention: 2 districts that received DEC and COMBI (+), 2 districts that received DEC-albendazole and COMBI (++).  **4) Insight-driven segmentation**:  - Use of different media channels for rural (e.g. loudspeaker) and urban (e.g. mass media) settings.  - A high number of FPAs were involved per findings of baseline studies.  **5) Co-creation**: NA |
| **Intervention ID**: I5-TRA  **Author, year:**  Atkinson et al., 2014; Lange et al., 2014; Lange et al., 2017; Baunach et al., 2012; Stanford et al., 2016; Lange et al., 2016; Taylor et al., 2012; Lange et al., 2012; Lange et al., 2015; Jones et al., 2015**;** Lange, JR Atkinson, et al., 2013; Lange, J Atkinson, et al., 2013  **Year of implementation**: 2010- 2012  **Location:**  Northern Territory (NT), Australia, Western Pacific, High income | **NTD**: Trachoma  **Targeted stream:**  Midstream  **Public:**  - Health, education and community support settings staff  - Children and their carers.  **Setting:**  Work settings: clinics, schools, community support workplaces (e.g. child care centers, family well-being centers, sport and recreation programs)  63 remote Aboriginal communities in the Northern Territory (NT)  **Sample size:**  Study design: cross-sectional pre-post study using convenience sample  Pre-intervention KAP survey: 272 participants  Post-intervention KAP survey: 261 participants | **Aim:** To improve the knowledge, attitudes and practices of health, education and community support settings staff and their ability to teach others about trachoma prevention, and to improve hand and facial hygiene practices among children and carers.  **Behavior**: Clean faces and have good hygiene practices.  **Description:**  - Culturally appropriate intervention in partnership with multiple organizations and strong key stakeholder involvement.  - Activities implemented in staggered delivery over one-year.  - Methods mix included: Trachoma Story Kits, e-learning module, Australian Football League (AFL) footy clinics with hygiene stations, a mascot, merchandising, advertising via TV and radio.  **WHO NTD strategy:**  Water, sanitation and hygiene | **Concepts:** **1) Social Behavioral influence**:  Reduce acceptance of dirty faces as normal and teach others about trachoma prevention.  **2) Public / people orientation:**  - The intervention development was informed by findings from baseline KAP survey with 72 staff members at the KWHB Katherine office and six remote communities (August 2010 - March 2011).  - The KAP surveys were piloted.  **3) Social offerings**:  - Health, education and community support settings staff increased their knowledge of trachoma and trichiasis through the use of varied culturally sensitive resources, to be able to teach others about trachoma prevention.  - Children and their carers learned about appropriate hand and face hygiene practices via footy clinics.  **4) Relationship building**:  - KAP questionnaire developed and piloted with community members.  - A culturally appropriate toolkit with resources (the Trachoma Story Kits) were developed before the intervention by a partnership between Katherine West Health Board (KWHB), the Indigenous Eye Health Unit (IEHU) at the University of Melbourne and the Centre for Disease Control, Department of Health Northern Territory; and in consultation with the Ngumpin Reference Group (NRG) (group of past and present Aboriginal Health Workers and Board Members of KWHB).  - Partnership between the University of Melbourne and the Melbourne Football Club (MFC) for implementing trachoma football hygiene clinics in the NT. 16 partner organizations run the football clinics. 2 Indigenous players were selected by the MFC as trachoma ambassadors.  - Community engagement through footy clinics events.  - Creation of local employment opportunities for Indigenous people. | **Techniques:** **1) Integrated intervention mix**:  - Trachoma Story Kits: clinic, community and school flipcharts, teacher and student workbooks, poster series, trachoma resources book, key messages, correcting myths, WHO trachoma grading card, surveillance report, Milpa tatoos, Milpa stamp, trachoma resources DVD.  - Trachoma e-learning module based on Trachoma Story Kit developed for urban health practitioners doing short-term contracts in the remote Indigenous communities  - In schools, lessons plans in line with the curriculum were developed  - Australian Football League (AFL) footy clinics with hygiene stations.  - Hygiene stations: water source, water containers, tables, mirrors, bins and bin liners, tissues, pump-pack hand soap, paper towels, basins for water collection, posters, banners, tattoos, wristbands, "Milpa" the mascot, drinking water and cups.  - Participation in the hygiene stations required the children to practice the promoted behaviors. Upon completion of the process, participants were rewarded with promotional merchandising.  - Childhood setting safety mirrors installed at 50 work settings  - Football trachoma ambassadors present during AFL footy clinics and featured in promotion materials.  - Messages promoted: "Clean Faces, Strong Eyes", "Wash your face whenever is dirty".  - Mascot: Milpa the trachoma goanna named after the Warlpiri word "eye".  - Promotion at Australian Football League games  - Live community performances  - Posters, advert-TV, advert-Radio  **2) Competition analysis and action**:  - The intervention addressed embarrassment and community shame about personal hygiene, and that "dirty faces" were considered as normal in young children of remote Indigenous communities by staff from clinics, schools and community workplaces.  **3) Systematic planning and evaluation**:  - Development of resources, design of the intervention and evaluation tools, and evaluation carefully planned. Intervention implemented along different moments and in partnership. Partners leveraged in each-others know-how.  - Pre-post surveys to assess changes in trachoma KAP by staff in the three work settings  - Pre-intervention KAP survey: August 2010 - June 2011  - Intervention: 2011- 2012  - Post-intervention KAP survey: February-July 2012  **4) Insight-driven segmentation**:  - Resources that were culturally appropriate and responsive to remote community contexts were developed and used.  - Materials were adapted to each target audience and setting.  - Work-setting staff selected as target audience as they could teach and deliver message to children and their carers.  - AFL clinics were a fun way to engage children and adults.  - AFL players helped address the sensitive issue of personal hygiene by serving as role models.  **5) Co-creation**:  - Trachoma Story Kits co-developed with the NRG in a 12-month consultation process. Tools launched in August 2010.  - The NRG and Aboriginal Health Workers from the KWHB recommended placing resources at clinics, schools and community work-places. |
| **Intervention ID**: I6-LYM  **Author, year:** King et al., 2011  **Year of implementation**: 2003 - 2004  **Location:**  American Samoa, United States, Americas, high income | **NTD**: Lymphatic filariasis (LF)  **Targeted stream:** Midstream.  **Public:**  General population.  **Setting:**  Multiple, see integrated intervention mix.  **Sample size:**  - KAP survey (pre-MDA 2003): 153 respondents  - Key informant interviews (post-MDA 2003): leaders from 45 churches  - Coverage survey (post-MDA 2004): 278 respondents. | **Aim:** To increase coverage of the annual mass drug administration with albendazole and DEC.  **Behavior:** Take treatment (albendazole and diethylcarbamazine - DEC) in MDA.  **Description:**  Modifications to the annual lymphatic filariasis MDA campaign based on formative research to increase coverage. Changes made in drug distribution channels and dissemination channels. Intensified stakeholder engagement (e.g. church leaders) and use of mass media. Messages focused on promoting pill-taking and disease recognition.  **WHO NTD strategy:**  Preventive chemotherapy and transmission control (PCT) | **Concepts:** **1) Social Behavioral influence**:  Participate in annual mass drug administration campaign (MDA) with albendazole and diethylcarbamazine (DEC) and take the pills.  **2) Public / people orientation:**  - Formative research informed modifications in the annual campaigns and consisted on: (i) analysis of coverage of previous MDA campaigns (2000 - 2002); (ii) distributor evaluation after the 2002 MDA through focus groups and a self-administered questionnaire; (iii) KAP survey administered by volunteer students to community members prior to the 2003 MDA.  **3) Social offerings**:  Multiple communication channels, community mobilization activities and increased drug distribution channels were used to increase awareness/knowledge about LF and the LF MDA campaigns, and to motivate participation in the campaigns to eliminate transmission of filariasis.  **4) Relationship building**:  - Church leaders were engaged to support in disseminating messages and distributing DEC and albendazole to churchgoers after church services.  - Island news media involved.  - Increased involvement of school principals and workplaces employers.  - School administrators and employers engaged to schedule distribution dates and inform in advance. | **Techniques:** **1) Integrated intervention mix**:  - MDA with DEC and albendazole.  - Increased number of distributors.  - Condensed the campaign time period from 4 months to 2 months.  - Distribution of DEC and albendazole at all district health centers during the MDA period, churches, schools, workplaces, crowded public venues (e.g. bingo halls, shopping centers, airport).  - Events held in public venues (e.g. radio broadcasts).  - Messages focused on specific actions (e.g. pill-taking).  - Prior announcement of upcoming MDA dates in local newspapers.  - Radio: 30 second spots, 2-3 times per day on all local radio stations; morning show interviews.  - TV: news interviews during the campaign, filariasis discussions during regular health programming at the start of the campaign; spots before news and sport events.  - Radio/TV spots with testimonials, skits and news announcements promoting pill-taking and informing distribution places.  - Group presentations, LF affecter person testimonial.  - Engagement of church leaders, island news media, schools and workplaces.  **2) Competition analysis and action**: NA  **3) Systematic planning and evaluation**:  - Studies conducted during formative research, for monitoring and evaluation.  *After the 2003 MDA:*  (i) distributor evaluation (i.e. nurses, program directors, health assistants, volunteers) through focus groups and a self-administered questionnaire after the 2003 MDA, (ii) key informant interviews with religious leaders.  *After 2004 MDA:*  (i) household coverage survey.  **4) Insight-driven segmentation**:  - House-to-house distribution of drugs abandoned and church leaders were actively engaged addressing findings from formative research.  - Avoidance of public distribution places that could be overly intrusive.  **5) Co-creation**: NA |
| **Intervention ID**: I7-LYM  **Author, year:**  Krentel et al., 2006  **Year of implementation**: 2002  **Location:** Alor District, Indonesia, South-East Asia, lower middle income | **NTD**: Lymphatic filariasis (LF)  **Targeted stream:** Midstream  **Public:**  - General population of 5 villages.  **Setting:**  Villages; 3 located on the coastline (Alila Timur, Maukuru, Pante Deere), 1 in a montainous area (Probur) and 1 in a rice growing plateau (Kamot)  **Sample size:**  - Pre-intervention KAP survey: 375 respondents (51% males, 49% females).  - Post-intervention KAP survey: 386 respondents (51% males, 49% females). | **Aim:** To motivate the population to take the required treatment for filariasis that was offered during MDA for prevention and control.  **Behavior:** Take treatment (DEC and albendazole).  **Description:**  - 5 weeks pilot MDA intervention with community involvement.  - Training for interviewers and community volunteers  - Health promotion campaign using oral (i.e. film, song), visual material (e.g. poster) and interpersonal communication by community volunteers, drug distributors and health workers.  - MDA with DEC and albendazole.  **WHO NTD strategy:**  Preventive chemotherapy and transmission control (PCT) | **Concepts:** **1) Social Behavioral influence**:  Take the Lymphatic Filariasis treatment provided during MDA.  **2) Public / people orientation:**  - Findings from previous public health awareness activities and surveys and from discussions with civil society and key government individuals informed the campaign should he rooted in the community.  - KAP questionnaire tested in one village.  - Baseline KAP survey findings informed the development of the health promotion campaign.  - Communication material tested through focus groups were men and women were separated and through 12 interviews with people selected randomly on the streets. Editions made post-testing.  **3) Social offerings**:  - Community and health authority involvement in the planning and implementation of the health promotion campaign and in MDA activities.  **4) Relationship building**:  - Parasitologists from the University of Indonesia were involved in the design of the communication material to check the use of the terminology.  - Feedback from the health authority and villagers to the campaign slogan.  - Community volunteers were trained by health staff. | **Techniques:** **1) Integrated intervention mix**:  - 2-day training for interviewers on questionnaire administration.  - Community volunteers trained to conduct the village census and to disseminate information.  - MDA: Single dose of DEC (based on 6 mg/kg translated into age-based dosing table for simplicity) combined with albendazole (400mg). Done 10 days after the information campaign finished.  - Other medicine available to respond to adverse reactions through village drug distributors.  - Drug delivery: house-to-house or multiple treatment posts.  - Village drug distributors received medicine to administer to people with adverse reactions to treatment.  - Health staff remained the first night after the MDA to help respond to adverse reactions.  - Campaign slogan: "Berantas filaria" (Eliminate Filaria) with the word "Alor Sehat 2010" (Alor Healthy 2010) written bellow.  - Oral communication materials: 18-minute film with content from the Alor District and the WHO. Song using traditional music genre ("poco-poco"); a cassette with the song was given to minibus drivers and health centers to play.  - Visual communication material: A flipchart, poster, brochure and a sticker. Used consistent colors, images and fonts.  - Community volunteers (i.e. drug distributors and village leaders) supported in intervention activities.  **2) Competition analysis and action**:  - The intervention addressed the fear to adverse reactions to treatment.  **3) Systematic planning and evaluation**:  - Strategy and concept development (10 weeks) included design, testing and revision.  - Preparation of the campaign and drug distribution (3 weeks) included training of health staff and of community health workers (CHW).  - Campaign implementation (5 weeks).  **4) Insight-driven segmentation**:  - The 5 intervention villages were divided in clusters. Interviewees were selected randomly seeking an even participation of men and women.  - Based on findings from baseline study the communication material used simple language, visual and oral forms of media to reach most people, and addressed potential adverse reactions to treatment.  **5) Co-creation**:  - The interview team (individuals from the district health authority in the district capital, from the provincial capital or privately hired) received a 2-day training to administer the KAP baseline and evaluation surveys.  - Communication materials tested via focus group discussions with villagers and civil society members.  - A local music group collaborated to develop the song.  - Drug delivery method was decided between the health workers and the villagers. |
| **Intervention ID**: I8-LEP  **Author, year:**  Salgado, 1993; Williams et al., 1998; Wong, 2002; Brown, 2006  **Year of implementation**: 1990 - NA (Possibly 1994-1996)  **Location:** Sri Lanka / South-East Asia / Lower middle income | **NTD**: Leprosy  **Targeted stream:** Downstream / Midstream  **Public:**  - The key audiences were (1) Individuals with suspicious lessons on the skin, (2) Health care providers and (3) the general public  - Advertising campaign targeted young and middle-aged adults  **Setting:**  Country  **Sample size:**  - Pre-intervention KAP study: 1000 people from 4 districts | **Aim:** To prompt individuals with suspicious lessons on the skin to seek treatment by self-referral, to have health care providers to identify and refer leprosy cases for treatment, and to reduce fear to leprosy among the general population.  **Behaviors:**  - Seek diagnosis and treatment.  - Identify and refer leprosy cases for treatment.  - Reduce fear to leprosy (not a WHO priority strategy).  **Description:**  - National social marketing intervention.  - Training to health care staff provided.  - Increase in health centers capacity to provide quality care for Leprosy.  - MDT (Multi Drug Therapy) treatment promoted.  - Use of social advertising via different channels (e.g. TV, radio, spokesperson).  - Education sessions conducted in remote populations.  - Relationship with regional health authorities sustained.  **WHO NTD strategy:**  Innovative and intensified disease management (IDM) | **Concepts:** **1) Social Behavioral influence**:  - For individuals with suspicious skin lessons, seek diagnosis and comply to MDT (Multi Drug Therapy) treatment.  - For health care providers, recognize early signs of leprosy and refer cases for treatment.  - For the general public, attitudinal and behavioral change aiming at dispelling leprosy stigma and creating a friendlier environment.  **2) Public / people orientation:**  - KAP surveys and focus groups with patients and their families to understand perceptions about leprosy.  - Formative research informed about the poor awareness of the early signs of leprosy and fear of the disease, including among health care staff. This was addressed by the campaign messages and through training for health care providers.  - Focus groups to test the messages and to follow up their acceptability.  **3) Social offerings**:  - Treatment to cure leprosy without deformities and social acceptability.  - Primary health care staff and medical officers in out-patient departments of hospitals received training to identify leprosy and refer suspicious cases to the leprosy staff. This improved the quality of care.  **4) Relationship building**:  - The Ciba-Geigy Leprosy Fund proposed the intervention to the Sri Lankan Government and the other intervention partners.  - Government approval to design and implement the intervention.  - The Sri Lanka Anti-Leprosy Campaign was actively involved in all aspects of the intervention  - Regular meetings with regional health authorities to sustain political support.  - Strong links forged with dermatologists and regional health authorities.  - An actor was a spokesperson of the campaign  - Key leaders participated health education activities and discussions | **Techniques:** **1) Integrated intervention mix**:  - Primary health care staff (>4000) and hospital outpatient doctors (>1000) were trained to improve the skills to diagnose and treat the disease.  - Increase in the number of leprosy treatment points from 75 to 225, and of selected clinics.  - Medical care and treatment for citizens is free at government health centers  - MDT in calendar blister packs was provided for free by the Ciba-Geigy Leprosy Fund  - In some occasions people received reimbursement for their travel costs and lost pay  Place  - MDT was offered through the Sri Lankan leprosy control services and dermatological clinics.  - To respond to increased demand, the number of treatment centers and of selected clinics were increased, and primary health staff and medical officers received training for leprosy detection and referral.  - Messages highlighted: (i) the availability of a modern drug (Multi Drug Therapy MDT), (ii) that leprosy can be cured without deformities and (iii) the need to seek early help. The messages promoted differed from those of other diseases and were disseminated in a positive manner.  - A logo was created.  - Television (teledrama and ads), radio (soap opera, spots), newspapers, posters, hoardings, pamphlets, bus stickers and other printed material. All with one theme, message and source.  - Famous actor playing the role of a leprosy patient in a TV teledrama was spokesperson of the TV campaign.  - 1-week education sessions and "skin camps" held in remote areas.  - Skin camps: detect leprosy and free treatment for the skin.  - 5000 opinion leaders (priests, school teachers, village heads) participated in health education activities.  - Doctors received pocket calendars with information about the leprosy clinics locations, dates and times.  - Sri Lanka's clergy received letters and health education material.  - School teachers received flip charts with information.  - Traditional healers received information about the disease and MDT.  **2) Competition analysis and action**:  - The intervention addressed misconceptions about leprosy (e.g. deformities), guilt and shame by leprosy sufferers and their lack of acceptance of the biomedical explanations of the disease, and the difficulty to recruit health care staff due to leprosy stigma.  **3) Systematic planning and evaluation**:  - Actions undertaken to respond to the expected increased demand for diagnosis: the network prepared to receive greater number of people; training in leprosy management was offered to GPs, paramedics, village leaders and other health staff, including Ayurvedic doctors who were briefed.  *Monitoring*  - Post-test of the intervention after 6 months.  - Focus groups were done with regularity to follow up the acceptability of the messages  - Informal interviews conducted in public places to monitor impact and modify the campaign  -To monitor the epidemiological impact and sustain political support for controlling the disease, regular meetings were done with regional health authorities  *Evaluation*  - To monitor and evaluate the program, cross sectional surveys were conducted every 6 months  - Three years post intervention: Study to assess residual impact on knowledge, socio-cultural attitudes and practice, a study was conducted in 1997 among 1.800 non-affected persons (e.g. school teachers, midwives, 430 allopathic and ayurvedic health care providers.  **4) Insight-driven segmentation**:  - Pre-intervention KAP study informed the message content.  - Different messages were created for different target groups (young and middle-aged people, and doctors). The message for young adults focused on the curability of the disease and highlighted benefits of early treatment such as social acceptability by loved ones, getting married and having a family. The message targeting doctors served as a reminder to identify individuals with early signs of leprosy.  - Positive experiences shared by individuals seeking treatment were recreated in new communication material.  **5) Co-creation**: NA |
| **Intervention ID**: I9-SCH  **Author, year:** Freudenthal et al., 2006  **Year of implementation**: 2002  **Location:**  Kileo and Kivulini, Tanzania, Africa, Low income | **NTD**: Schistosomiasis  **Targeted stream:**  Downstream  **Public:**  Schoolchildren  **Setting:**  Two primary schools in the villages.  **Sample size:**  NA | **Aim:** Create enabling environments for schoolchildren and communities to adopt practices to reduce schistosomiasis transmission.  **Behaviors:** Create environments that facilitate the adoption of preventive/control practices (e.g. safe swimming places, school curriculum including schistosomiasis prevention).  **Description:**  - Intervention building on previous experience of research team in the communities.  - Screening of children for schistosomiasis and intestinal helminths.  - Treatment provided to infected children.  - School essay writing, video recorded dramas.  - Household sanitation observations by schoolchildren.  - Teachers participated in intervention development and implementation.  - Parents engaged.  **WHO NTD strategy:**  - Preventive chemotherapy and transmission control (PCT)  - Water, sanitation and hygiene | **Concepts:** **1) Social Behavioral influence**:  Create environments that facilitate the adoption of preventive/control practices.  **2) Public / people orientation:**  - The project team had implemented other research projects in the setting. Findings from these projects showing a high prevalence of schistosomiasis among children motivated the development of this intervention.  - Essay writing by 6-7 grade schoolchildren informed about their: (i) knowledge and perceptions about schistosomiasis and (ii) weekend chores.  - Household sanitation observations (e.g. presence of temporary or ordinary latrine).  **3) Social offerings**:  Use of participatory methods appropriate to the culture, to engage and facilitate dialogue among schoolchildren, teachers and community members about the schistosomiasis transmission cycle and preventive/control measures.  **4) Relationship building**:  - Previous experience with community members during a focus group study facilitated engagement.  - Meetings with school director and village leaders.  - Village leaders organized a community meeting in each village.  - Gradual involvement of teachers in screening procedures.  - Household sanitation observation form developed by a local NGO with a focus on primary healthcare. The NGO trained the pupils to conduct the survey. | **Techniques:** **1) Integrated intervention mix**:  - Screening in 2002 for schistosomiasis and intestinal helminths by research team and nurses from the local dispensary.  - Treatment for children infected: praziquantel (single oral dose of 40 mg/kg) for schistosomiasis, albendazole (single oral dose of 400 mg).  - A cohort of pupils screened again in 2003 and 2004. Teachers involved in the process (e.g. measurement of height and weight, offering meal prior to treatment).  - Prevention slogan developed by schoolchildren: "I am aware of kichocho (schistosomiasis). Are you?"  - T-shirts with slogan given to 6-7 grade pupils.  - School essay writing by children.  - Pupils and teachers had at least 3 moments to reflect about schistosomiasis: (i) while planning the dramas/songs, (ii)when performing them, and (iii) when viewing the video recordings of them.  - Children formed environmental health clubs and were "kichocho ambassadors" to teach younger children and community members about schistosomiasis.  **2) Competition analysis and action**: NA  **3) Systematic planning and evaluation**:  - The project was implemented within existing health institutions to facilitate continuity of actions post-intervention.  **4) Insight-driven segmentation**:  - Dramas, songs and dances were used because they are part of African performance traditions.  **5) Co-creation**:  - During schistosomiasis and intestinal helminths screening schoolchildren participated in discussions to develop slogans for t-shirts.  - Grade 6 pupils conducted household sanitation survey. Modification to the survey done by them and the teachers.  - Video recorded dramas, songs an dances by the schoolchildren.  - In one school teachers and students developed script and recorded a video using it. In the other school parents were invited to visit the school and listen the songs and dramas. In both cases the performances were video recorded.  - Feedback meetings in schools and the community to discuss findings from school essays, fieldnotes and videotapes. As a result, community members take the initiative of creating safe swimming places.  - Grade 6-7 pupils performed dramas about schistosomiasis, created songs and dances. These were video recorded.  - Workshop with teachers and grade 7 pupils on how to make an educational video about schistosomiasis. |
| **Intervention ID**: 10-SOI  **Author, year:** Bieri, Yuan, et al., 2013; Bieri, Gray, et al., 2013   **Year of implementation**: 2010 – 2011  **Location:**  Linxiang City District, Hunan province, Chin, Western Pacific, Upper middle income | **NTD**: Soil-transmitted helminths  **Targeted stream:**  Downstream  **Public:**  9-10 years old schoolchildren  **Setting:**  School  **Sample size:**  Study design: single blind, unmatched, cluster-randomized intervention trial. 38 schools (clusters), schoolchildren 9-10years old, control: 893 children, 19 schools; intervention: 825 children; 19 schools. 976 boys and 739 girls (no sex information available for 3 students) | **Aim:** Increase knowledge about soil-transmitted helminths (STHs), promote behavior change and reduce the rate of infection.  **Behaviors:** Handwashing after toilet use and adoption of other preventive behaviors.  **Description:**  - Culturally tailored 9-month intervention  - Teacher training workshop before baseline survey.  - Control package: health-education poster normally displayed in schools  - Intervention package: 12-minute animated narrative cartoon "The Magic Glasses", display of same poster as in control group, classroom discussions, drawing and essay writing competitions, pamphlet with transmission and prevention information  - 100-liter water container with a gravity tap installed at all intervention and control schools  - Albendazole administered to children found positive for STHs infection during baseline and follow-up.  **WHO NTD strategy:**  Water, sanitation and hygiene | **Concepts:** **1) Social Behavioral influence**:  Handwashing after toilet use and adoption of other preventive behaviors  **2) Public / people orientation:**  - Desk research: previous video-based studies, behavioral theories, didactic principles, teaching experiences, Chinese animation history and Chinese cartoons.  - Formative research for video development and production: Household survey, KAP questionnaire, 'Draw and Write' assessment, key informant interviews and field/household observations.  - The video was pilot tested in 6 schools with an audience of schoolchildren (N=80), teachers (N=11) and invited parents (N=9). After the video, the audience filled out a questionnaire to assess if the key messages were understood and the answers were discussed in small focus groups.  - Behavioral theories considered: Social Cognitive Theory, Health Belief Model, Transtheoretical Model, and the Cognitive Theory of Multimedia Learning.  **3) Social offerings**:  Involvement in the formative research and in the development of the educational package. This package was adapted to the local context and culture.  **4) Relationship building**: - Early involvement of the community, teachers, parents, schoolchildren, health and education officials and health workers.  - Chinese government supplied albendazole, which was paid by the Hunan Institute of Parasitic Diseases. | **Techniques:** **1) Integrated intervention mix**:  - Teacher training workshop before baseline survey.  - Educational cartoon video "The Magic Glasses" informing about transmission and prevention.  - Classroom discussions, drawing and essay competitions, pamphlet with transmission and prevention information.  - Albendazole (400 mg single oral dose) administered to children found positive for STHs infection during baseline and follow-up.  - 100-liter water container with a gravity tap installed at all intervention and control schools.  **2) Competition analysis and action**: NA  **3) Systematic planning and evaluation**:  - Steps to develop the video: formative research, production, pilot testing and revision.  - Baseline and follow-up evaluation: KAP questionnaire, parasitologic survey, behavior observation.  - Treatment adverse events recorded.  **4) Insight-driven segmentation**:  - Key messages of the video incorporated everyday situations of the target audience.  - Favorite children's cartoon characteristics (e.g. funny, colorful, fast-paced) were integrated into the cartoon.  - Cartoon scenes portrayed local risk behaviors observed during formative research.  - Culturally-tailored educational package.  **5) Co-creation**:  - Several brainstorming sessions of multidisciplinary team (epidemiologists, education experts, animators and scriptwriters) to draft the cartoon narrative. Chinese scientists consulted on Chinese cultural aspects.  - Key informants including teachers, doctors and parents and schoolchildren contributed to video development.  - Chinese researchers and educators participated in development and piloting of KAP questionnaire. |
| **Intervention ID**: I11-DEN  **Author, year:** Escudero-Támara and Villareal-Amaris, 2015  **Year of implementation**: NA  **Location:** Sincelejo, Colombia, Americas, upper middle income | **NTD**: Dengue  **Targeted stream:** Downstream  **Public:** Schoolchildren family members.  **Setting:** Household  **Sample size: -** Sample: 54 schoolchildren family members. The sample was divided in two groups of 27; 88.9% were women.  - Study design: Pre/post intervention, convenience sampling | **Aim:** To prompt behavior change to eliminate breeding places of dengue vector.  **Behavior:** Eliminate dengue vector breeding places.  **Description:**  The Precaution Adoption Process Model was used to segment and deliver the intervention to families according to the stage of the model at which they were. Some communication and participation activities targeted all participants. Elements of the communication for behavioral impact (COMBI) model were applied.  **WHO NTD strategy:**  Vector ecology and management | **Concepts:** **1) Social Behavioral influence**:  Eliminate breeding places of dengue vector at the household.  **2) Public / people orientation:**  - 3rd and 4th grade primary school students answered a questionnaire where they selected breeding sites present in their houses. The Public Health Office of the Municipality Health Secretary confirmed the presence of positive breeding sites in the selected households.  - Knowledge test and semi structured interviews to understand the motivational and cognitive aspects influencing the current behavior in relation to the desired behavior.  -The Precaution Adoption Process Model was used to identify in which stage intervention participants were in relation to the desired behavior, and to implement activities.  **3) Social offerings**: NA  **4) Relationship building**:  - Involving 3rd and 4th grade students in identifying the houses with breeding places helped reach their families and engage potential intervention participants.  - The Public Health Office of the Municipality Health Secretary was involved in the entomological assessment and provided information about the dengue epidemiological situation of the municipality.  - Intervention activities helped to engage the audience. | **Techniques:** **1) Integrated intervention mix**:  - Elements of the communication for behavioral impact (COMBI) model were used  - Community mobilization: Collective and participatory actions were implemented.  -Activities targeting all participants: (1) Educational session: (1.1) Inform the neighbor: Educate three close neighbors about dengue preventive actions (1.2) Game "Beat Dengue" using wall newspapers, flyers, models showing dengue transmission and images with disease sequence; (2) Journey to collect unusables; (3) Journey to wash and cover the family tanks; (4) Radio program "Against the mosquito for your health".  - Activities per stages of the Precaution Adoption Process Model:  - *Stage 1 - Ignores the problem*: Motivational workshops  - *Stage 2 - Recognizes the problem and Stage 3- Recognizes his/her own susceptibility*: Motivational and cognitive sessions  *- Stage 4 - Decided what to do and Stage 5 - Decided to act*: Discussion groups, flyers and the game "Beat Dengue" distributed. Periodic visits.  - *Stage 6 - Action and Stage 7- Maintenance stages*: Demonstration workshops of washing water collection tanks where participants could provide feedback together. Collective actions implemented.  **2) Competition analysis and action**: NA  **3) Systematic planning and evaluation**:  *Monitoring*  - Participants behavior was monitored to determine changes in the stages of the Precaution Adoption Process Model.  *Evaluation:*  - A survey was implemented at the beginning and 6 months after the intervention to know about disease knowledge, vector behavior and dengue prevention measures  - Pre and post semi-structured interviews to know about dengue risk perceptions, risks of illness or death and severity  - Pre and post check list to assess the presence of breeding places at the households  **4) Insight-driven segmentation**:  - The intervention was designed and implemented considering the stage in which participants were in relation to the Precaution Adoption Process Model. The stages used for this intervention were: (1) Ignores the problem, (2) Recognizes the problem, (3) Recognizes his/her own susceptibility, (4) Decides what to do, (5) Decides to act, (6) Action, (7) Maintenance  **5) Co-creation**: NA |
| **Intervention ID**: I12-DEN  **Author, year:** Abeyewickreme et al., 2012; Arunachalam et al., 2010   **Year of implementation**:  2009 - 2010  **Location:**  Gampaha District, Sri Lanka, South-East Asia, lower middle income | **NTD**: Dengue  **Targeted stream:** Midstream  **Public:**  Community members.  **Setting:**  Households located in rural and per-urban clusters.  **Sample size:**  - Prospective experimental study. Randomly selected clusters.  - 2 high and 2 low transmission clusters in the treatment arm. 803 households in the intervention group.  - 2 high and 2 low transmission clusters in the control arm. 790 households in the control group. | **Aim:** Vector ecology and management.  **Behaviors:**  - Compost biodegradable waste.  - Properly manage solid waste.  **Description:**  - 12-month intervention with community mobilization for waste management.  - Stakeholder involvement, informational sessions, community-led activities, introduction to household level composting systems, improved garbage collection by local authorities.  - The intervention was part of a South-East Asia multi-country study on community ecosystem management for dengue vector.  **WHO NTD strategy:**  Vector ecology and management | **Concepts:** **1) Social Behavioral influence**:  Properly manage solid waste at the household level.  **2) Public / people orientation:**  - A situation analysis consisting in household surveys, neighborhood background surveys and entomological surveys, was conducted to identify the strengths and weaknesses of the system in regards to the management of solids and dengue control. This analysis informed the focus on small discarded containers.  - Baseline studies pilot tested.  - Baseline household survey carried out in 1,585 households (April-May 2009)  - Stakeholder analysis prior the intervention to: (i) understand their characteristics and select the primary and secondary stakeholders, and to (ii) determine possible roles in the intervention.  - A gender analysis using data collected through key informant interviews and focus groups. Findings informed the focus on women as they could contribute better to the waste management system.  **3) Social offerings**:  - Information sessions, tools, community-based activities, involvement of key stakeholders and structural behavioral determinants were addressed to facilitate waste management at the household level.  **4) Relationship building**:  - Local government agencies and public health officials sensitized since the start of the project.  - Results of the situation analysis discussed and intervention effectiveness assessed with community representatives during focus groups.  - Through engagement, political support obtained from local authorities to facilitate garbage collection services.  - Female and male volunteers selected during field visits, focus groups and key informant interviews. They organized activities, were intermediaries between the community and intervention staff and collaborated with the local government authorities. | **Techniques:** **1) Integrated intervention mix**:  - The households received for free: (a) 150 liter compost bins made of plastic, (b) three bags made of polythene for separation of solid waste (plastic, glass and paper), (c) different varieties of vegetable and fruit plants for home gardening projects.  - Compost bins and garbage disposal distribution to households included (a) a lecture/demonstration on solid waste management conducted by an expert, and (b) a brief presentation on home gardening and organic farming.  - Plants for home gardening were given to households, 3-4 months after distributing the compost bins, so that they could use the compost made at home as a fertilizer for home gardening projects.  - Control clusters had access to information and services that are routinely available to the general public.  - In the intervention clusters, households went to a pre-determined place to receive the compost bins and garbage disposal bags. The distribution was done under patronage of a religious and/or political leader.  - A one-day awareness raising program with community volunteers was carried out by the intervention investigators.  - An awareness program directed to school children took place in 8 schools in all intervention clusters, with active participation of senior students, teachers and principals.  - A total of 8 cleaning campaigns of the environment, locally called "shramadana" were organized by volunteer groups, each had on average 100 participants.  - The Central Environmental Authority sold at a 50% discount rate the compost bins and the garbage bags that were given to the households.  - Intensified garbage collection during/after in the intervention clusters. Regular collection in the control clusters.  **2) Competition analysis and action**: NA  **3) Systematic planning and evaluation**:  - Several key informant interviews and focus groups were conducted at the planning and monitoring stages.  - To ensure sustainability of the activities post-intervention, collaboration between local government authorities and volunteers was facilitated and volunteers were encouraged to create their "Environmental and Health Associations".  - Before the completion of the project the investigators passed to the local health authorities the role of providing technical assistance and coordination.  *Monitoring*  - Entomological and sociological surveys were conducted at baseline (round 1: April-May 2009) and follow up (round 2: August-September 2009, round 3: February-March 2010, round 4: August-September 2010) to inspect the presence of *Aedes* larvae/pupae in water holding containers  - Monthly monitoring and quarterly assessments (e.g. checklists, quantitative/qualitative questionnaires) conducted in each cluster to monitor progress  *Evaluation*  - Community mobilization was assessed through: (a) Focus groups (FGDs) with community representatives and volunteers of the 4 intervention clusters in the planning and monitoring stage; (b) in-depth key informant interviews (KIIs) conducted at the planning and monitoring stages, and (c) by collecting the views of heads of households during surveys carried out in the intervention clusters (November-December 2009, July-August 2010) and in the control clusters (November-December 2009, December 2010).  - Entomological impact using as main outcome variable (pupae per 100 persons (PPP) index).  **4) Insight-driven segmentation**:  - Per results of focus groups the intervention focused on women because they were key players in the processes of cleaning homesteads and solid waste management at the household level.  - Results of the situational analysis informed about infrequent waste disposal services. Support was requested from the local government for garbage collection in the intervention clusters.  **5) Co-creation**:  - Volunteer groups organized environment cleaning campaigns in their clusters. Local authorities, health workers (PHI, MOH), and religious leaders of the area participated. |
| **Intervention ID**: I13-DEN  **Author, year:** NK Ibrahim et al., 2009  **Year of implementation**: 2005 - 2006  **Location:** Jeddah, Saudi Arabia, Eastern Mediterranean, high income | **NTD**: Dengue  **Targeted stream:**  Downstream  **Public:**  Female students, teachers and supervisors in high schools  **Setting:**  - 20 female high schools (16 government, 4 private) and 2 school supervision centers  - Study design: Multistage, stratified, random sampling method to select high schools and students  **Sample size:**  - Pre-intervention KAP survey: female students (n=2693), teachers (356) and (115) supervisors  - Post-intervention KAP survey: students (n=2433), teachers (n=310) and supervisors (n=70). | **Aim:** Improve knowledge, attitudes and practices related towards dengue fever (DF).  **Behavior:**  - Improve prevention practices (e.g. visit medical doctor if suspected DF, use of containers, use of mosquito repellents).  **Description:**  - Health education and discussion session using audiovisual aids  - Brochures, gifts with educational messages, posters, stickers and a CD-ROM with the lecture and film were used for dissemination  - Educational models constructed  **WHO NTD strategy:**  Vector ecology and management | **Concepts:** **1) Social Behavioral influence**:  Improve knowledge, practices and prevention/control practices (e.g. visit medical doctor if suspected DF, use of containers, use of mosquito repellents) towards dengue fever.  **2) Public / people orientation:**  - Findings of a KAP survey with high school female students, teachers and supervisors informed the intervention design.  **3) Social offerings**: NA  **4) Relationship building**: NA | **Techniques:** **1) Integrated intervention mix**:  - Health education and discussion session: 20 minutes lecture, audiovisual aids (film and cartoon) to the study sample, other school students, teachers, supervisors and school administrators.  - Information disseminated via brochures, gifts with educational messages, posters, stickers and a CD-ROM with the lecture and film sessions.  - Educational models constructed showing risk environments for mosquito breeding  - After the discussion sessions, study sample was asked to share the message with family and friends.  **2) Competition analysis and action**: NA  **3) Systematic planning and evaluation**:  - Questionnaire construction, development of message and of educational materials by 4th year medical students of the Department of Family and Community Medicine, King Abdul-Aziz University. They also conducted fieldwork.  - Design and implementation in 5 phases: (i) formative research, developing (ii) behavior change recommendations, (iii) educational messages, and (iv) educational material, and (v) producing the educational material  - Implementation and evaluation in 3 phases: (i) pre-intervention questionnaire, (ii) intervention delivery, and (iii) post-intervention questionnaire.  - Pre-intervention cross sectional survey conducted in 20 female high schools to assess knowledge, attitudes and practice (KAP) of high school female students from 15 to 21 years of age, teachers and supervisors.  - Post-intervention survey 1 week after the intervention.  **4) Insight-driven segmentation**:  NA  **5) Co-creation**: NA |
| **Intervention ID**: I14-SCH  **Author, year:** Hu et al., 2005   **Year of implementation**: 1992 – 2003  **Location:** Poyang Lake area, China, Western Pacific, upper middle income | **NTD**: Schistosomiasis  **Targeted stream:**  Downstream  **Public:**  Schoolchildren, adult women, adult men.  **Setting:**  6 villages located in the Poyang Lake area.  Villages: 1st Phase: experimental group (Fanhu - A), control group (Banshap - A'). 2nd Phase: Experimental groups: (Pianan - B, Xiejia - C, Nanyang - D), control group (Longkou - B').  **Sample size:**  Target population: (i) schoolchildren (n=120, 6-15 years), (ii) adult females (n=206, 16-60 years), (iii) adult males (n=194, 16-60 years). | **Aim:** To increase awareness and knowledge about schistosomiasis, reduce the frequency of infested water contact and increase compliance with praziquantel-based chemotherapy.  **Behaviors:**  -Decrease frequency of infested water contact (e.g. children - swimming/playing, women - washing clothes, men - fishing)  - Increase compliance towards chemotherapy  **Description:**  12-year intervention including a pilot.  Segmented offerings to three groups: schoolchildren, adult women, adult men consisting in health education and training.  Reward/punishment program for schoolchildren. Emphasis on praziquantel chemotherapy compliance on men.  **WHO NTD strategy:**  - Water, sanitation and hygiene.  - Preventive chemotherapy and transmission control (PCT). | **Concepts:** **1) Social Behavioral influence**:  - Reduce contact with snail infested water.  - Increase compliance with praziquantel-based chemotherapy treatment.  **2) Public / people orientation:**  - Pretest of evaluation questionnaire in a neighboring area.  **3) Social offerings**:  - Educational sessions and training used to increase knowledge, improve attitudes towards schistosomiasis control and chemotherapy treatment, and to reduce infested water contact and improve compliance with treatment.  **4) Relationship building**:  - School teachers engaged to support in the activities with schoolchildren. | **Techniques:** **1) Integrated intervention mix**:  - For all segments: Course on techniques for prevention of infection: video-tape, sample exhibition (e.g. adult schistosomes, intermediate host snails).  - Schoolchildren: Warning signs in high-transmission areas near them; reward or punishment (criticism) program with the support of teachers based on KAP evaluation results.  - Adult women: training course on the harmful effects of the disease and attitudes for schistosomiasis control. Use of multimedia (e.g. pictures, video-tapes, sample exhibits).  - Adult men: teaching focused on compliance with chemotherapy and early examination, use of video.  **2) Competition analysis and action**: NA  **3) Systematic planning and evaluation**:  Multi-staged approach used:  - 1st stage: examination of the short-term effects on disease control assessed comparing knowledge and attitude pre-intervention (1992) and post-intervention (1994). Study focused on two villages (Fanhu, Banshan).  - 2nd stage: Implementation and evaluation continued from 1994 to 1996. Four new villages incorporated to the study (Pianan, Longkou, Xiejia, Nanyang).  - Follow up of participants during a 12-year period (until 2003).  - Baseline survey in the experimental and control villages. Stool examination, single dose of 40mg/kg praziquantel. Stool examination one year after.  - Questionnaire: Questions related to KAP and snail infested water contact; conducted throughout the study to monitor changes over time.  - Recordings of compliance with chemotherapy.  - Evaluation population: Schoolchildren experimental (n=89), control (n=81); women experimental (103), control (n=101); men experimental (n=89), control (n=98).  **4) Insight-driven segmentation**:  - Segmentation of the population after baseline survey.  - Interventions develop for each segment.  **5) Co-creation**: NA |
| **Intervention ID**: I15-GUI  **Author, year:** Adeyanju, 1987  **Year of implementation**: NA  **Location:** Lagon and Ogun States, Nigeria, Africa, Low income | **NTD**: Guinea-worm disease (Dracunculiasis)  **Targeted stream:** Midstream  **Public:**  Community members  **Setting:**  Farming villages  **Sample size:**  - Two clusters of villages. Cluster one (experimental group): 7 villages in Ogun State, about 1.200 inhabitants. Cluster two (control group): 5 villages in Lagos State, about 1.500 inhabitants. The villages were 10 miles apart. | **Aim:** To increase knowledge and promote the adoption of preventive actions against guinea-worm resulting in the building of sanitary wells to reduce the incidence of the disease and of absenteeism from agricultural farming.  **Behaviors:** Adopt preventive measures (e.g. filtering and boiling water, using wells, avoiding ulcer contact with water sources).  **Description:** Intervention with community participation.  Three-month training using traditional communication methods to volunteer village health workers designated by the communities.  Community meetings to share knowledge, identify problems and seek solutions. Community-based action included making structural changes to facilitate adoption of preventive measures.  **WHO NTD strategy:**  Water, sanitation and hygiene | **Concepts:** **1) Social Behavioral influence**:  - Adopt guinea-worm preventive actions and collectively construct sanitary wells to have clean drinking water.  **2) Public / people orientation:**  - Limited lasting benefits from past experiences and reliance on government assistance motivated involving the community in the intervention.  - On-the-spot assessment of the situation, visits to some households selected randomly.  - Discussions with local leaders confirmed guinea-worm was considered an important problem for the community.  - Use of the PRECEDE model  - Baseline data collected via: (i) health committees where participants expressed needs and interests; (ii) diagnostic survey on local knowledge, beliefs, attitudes, values and practices.  - Behavioral, educational and administrative diagnoses.  **3) Social offerings**:  - Increased skills on preventive measures for guinea-worm, simple curative techniques and health services referral via a training program. Knowledge transfer from village health workers to community members. Community involvement in the implementation and mobilization of resources to facilitate structural changes (e.g. wells construction).  **4) Relationship building**:  - The Health Education Department, Federal Ministry of Health, had the initial idea for developing and implementing the intervention, which was accepted by the King and his chiefs (decision-makers).  - The King called for a meeting with leaders where the health educator and the student interns explained commitments and responsibilities that were accepted by the leaders. It was agreed that the interns and health educator would provide limited supervision, encouragement and external links, and that villagers would give moral and resource support to village health workers.  - Villagers selected a four-member health committee for each experimental village.  - Health committee selected and supervised a volunteer primary/village health worker per village based on selection criteria by the communities.  - Local religious leaders, school teachers and other volunteers administered baseline questionnaire in experimental and control villages. | **Techniques:** **1) Integrated intervention mix**:  - Three-month training program for the village health workers.  - Trainers: local government midwife, public health inspectors, 2 health educators from the national office.  - During/after training the health educators acted as consultants for the health workers to emphasize their role in the villages.  - Health care volunteers received small monetary incentive from the Health Department  - Training program conducted in afternoon sessions on Saturdays and Sundays held at a primary school site per decision of trainees.  - The training program used traditional communication methods to engage and educate participants (e.g. role plays, modeling, storytelling, songs, riddles, proverbs, humor, audio-visual aids, practical demonstrations).  Community mobilization  - Some villages initiated money collection to construct wells. Villages with limited economic resources considered other alternatives (e.g. use of water filters, avoid contact with water sources if infected).  **2) Competition analysis and action**:  - The intervention addressed people’s expectations on government agencies solving their problems, beliefs in traditional methods and barriers to adopt preventive measures.  **3) Systematic planning and evaluation**:  - The King and village leaders agreed with the plan and distribution of responsibilities presented by the intervention health educator and interns  - Goals: (i) Providing potable drinking water through community collective action - sanitary wells construction, (ii) reduce incidence of absenteeism from agricultural farming.  - Objectives: (i) 5 months post training program increased knowledge of preventive actions by household heads, (ii) 8 months post training program behavior changes noticed in the experimental villages, (iii) 18 months post training program at least one sanitary well constructed and available to use per experimental village.  *Monitoring*  - Periodic visits to the villages by the health educators to observe health workers in action, provide feedback and encouragement, and to facilitate the intervention progress.  *Evaluation*  - Short-term, intermediate results measured a few months after the training took place.  - Follow-up questionnaire based on the diagnostic survey.  **4) Insight-driven segmentation**:  - Training strategies developed considering the characteristics of the audience (e.g. literacy, structure, socioeconomic status, disease knowledge).  **5) Co-creation**:  - Regular community meetings were used by the village health workers (trainees) to share with the community what they had learned, identify community problems and identify possible solutions. |
| **Intervention ID**: I16-DEN  **Author, year:** Lloyd et al., 1992; Winch et al., 1991; Lloyd et al., 1994; Kendall et al., 1991  **Year of implementation**:  Whole duration: June 1989 - December 1990  Intervention: January - April 1990  **Location:**  Merida, Yucatan, Mexico, Americas, Lower middle income | **NTD**: Dengue  **Targeted stream:** Midstream  **Public:** Community men and women  **Setting:**  Intervention: 6 communities equivalent to 2 periurban neighborhoods (colonias)  Control: 6 communities equivalent to 2 neighborhoods  **Sample size:**  Baseline KBP survey: 577 women Larval survey: 616 house lots. | **Aim:** Elimination or control of larval production sites at the household.  **Behaviors**: After use empty containers that can hold water and turn them over or cover them to prevent the accumulation of water between uses. Tires and bottles prioritized.  **Description:**  - 4-month intervention. - Community-wide meetings, home visits and photo sessions. - Community based groups designed 3 pamphlets (by/for the specific target group), 2 photonovels and 1 invitation to a community meeting.  - Door-to-door education by fieldworkers using materials and mounted samples of mosquitoes.  **WHO NTD strategy:**  Vector ecology and management | **Concepts:** **1) Social Behavioral influence**:  - After use empty containers that can hold water and turn them over or cover them to prevent the accumulation of water between uses. Tires and bottles prioritized.  **2) Public / people orientation:**  - Formative research: (1) open in-depth semistructured interviews, (2) baseline Knowledge, Beliefs and Practices (KBP) survey to understand community responses to government dengue control programs; (3) entomologic survey to document Ae. aegypti larval production sites on 616 individual property lots in the area of study. All these informed behavior change recommendations and the need of involving the communities.  - Pre-intervention anthropological ethnographic research to understand community perceptions of dengue: 180 interviews in 2 rural villages and 4 colonias.  - Pamphlets pretested and modified accordingly.  **3) Social offerings**:  NA  **4) Relationship building**:  - Community groups were formed to develop messages and the educational materials  - A community group designed an invitation to participate in a community-wide meeting  - Field staff trained to deliver the message interactively. | **Techniques:** **1) Integrated intervention mix**:  - Product: 3 types of pamphlets, and 2 photonovels (similar to a comic book) using photographs of residents from each intervention colonias carrying out control activities were used as teaching aids.  - Place: Door-to-door distribution of material. Each household was visited twice by field staff who used the 3 pamphlets, mounted samples of mosquitoes and the photonovel to teach.  - An invitation to participate in the final community meeting was delivered to each household.  - Promotion: Message men pamphlet: "The tire which is in your backyard or workshop can cause the death of someone in your family"  Message women pamphlet: The mosquitoes which give us dengue can reproduce inside our houses".  **2) Competition analysis and action**:  - The intervention addressed community mis-perceptions of which mosquito breeding control measures are effective.  **3) Systematic planning and evaluation**:  - Intervention development consisted in 5 stages: (1) formative research, (2) developing recommendations for behavior change, (3) development of educational messages, (4) development and production of educational materials, (5) distribution of materials.  - Pretest and two post-test (one-week after, six months later).  **4) Insight-driven segmentation**:  - Householders perception of whether a container was disposable or nondisposable was used to provide recommendations for the control or elimination of containers.  - The intervention considered the multiple uses that each type of container could have to develop strategies for disposable and controllable containers.  - Community members segmented according to who is responsible (i.e. men, women) for the elimination or control of different types of containers. Messages and materials were designed specifically by/for the target audiences.  **5) Co-creation**:  - During community meetings, residents designed the layout and messages for pamphlets specific to their target group (e.g. women on pamphlet for women, women and men on pamphlet for families)  - Two photo sessions: one to determine the photonovel story line and the second to select the pictures and retake if needed. |
| **Intervention ID**: I17-DEN  **Author, year:** Leontsini et al., 1993; Kendall et al., 1991   **Year of implementation**: 1990  **Location:** El Progreso, Honduras, Americas, lower middle income | **NTD**: Dengue  **Targeted stream:** Midstream  **Public:**  8 neighborhoods grouped into 4 pairs, in the pair one randomly assigned to the intervention and the other to the untreated group.    **Setting:**  Neighborhood households  **Sample size:**  Anthropological interviews: 60 in total, 40 informants (37 females, 3 males)  Pre-intervention/post-intervention survey: 435 households. | **Aim:** Reduce Ae. aegypti larval infestation indices by promoting the control of 4 types of containers (i.e. tyres, pilas, can, drums).  **Behaviors:** Control or eliminate 4 types of containers (i.e. tyres, pilas, can, drums) that most frequently contain Ae. Aegypti larvae.  **Description:** 6-months intervention.  Project staff shared information about dengue and its prevention during meetings with community members/leaders.  Community health committees formed per initiative of the participants in 3/4 intervention neighborhoods.  1/2 day training workshop to health committees. Continuous training/support was provided to them.  Health committees with the support of the project staff planned activities: household visits to provide information about Ae. aegypti infestation problem and risk of dengue hemorrhagic fever (DHF), survey backyard, provide advice to control/eliminate containers. Advise to control Culex larvae was also provided. Clean-up campaigns and sewage maintenance organized and carried out by the health committees.  A calendar and a comic book was co-designed with health committees and distributed among householders.  Home visits were not possible in one intervention neighborhood, discussions were held during community meetings and material was distributed by project staff.  **WHO NTD strategy:** Vector ecology and management | **Concepts:** **1) Social Behavioral influence**:  - Reduce Ae. Aegypti larval production sites by controlling 4 types of containers: tyles, pilas, cans, drums.  **2) Public / people orientation:**  -Pre-intervention anthropological ethnographic research: 60 interviews, topics included: illness taxonomy and priority, febrile illnesses causes/treatments, types of common insects, mosquito control measures, familiarity with previous campaigns, illness prevention, health seeking behavior, community organization.  - Information about the community history and the structure of the 8 neighborhoods was collected.  - Entomological, ethnographic and socioeconomic data collected informed the design of the communication plan, intervention and evaluation.  **3) Social offerings**: NA  **4) Relationship building**: - Government vector control personnel conducted household survey for Ae. Aegypti larval production sites.  - Community members and leaders were reached via community meetings. During these meetings they proposed the creation of health committees. These committees with the training and continuous support from the project staff, helped to plan and implement the intervention.  - Community involvement in the planning and implementation of Ae. Aegypti control measures at community households. Householders in 3 of 4 intervention neighborhoods learned about dengue from other community members. Community members of one intervention neighborhood learned from project staff. | **Techniques:** **1) Integrated intervention mix**:  - In the four intervention neighborhoods community meetings used to disseminate information about dengue and its prevention, and to engage community members.  - Four dengue control measures promoted.  - Health committees organized and conducted community sewage campaigns and sewage maintenance work  - Health committees formed in 3 neighborhoods.  - Continuous training and support offered to health committees by project staff.  - In three out of four intervention neighborhoods: household visits by 2-3 persons of the health committees and project staff. This was not possible in one neighborhood.  - A calendar and a comic book with 4 household control measures was delivered to householders by the health committees or by the project staff in the neighborhood without a health committee.  **2) Competition analysis and action**: NA  **3) Systematic planning and evaluation**:  - Study design: Trial  Pre-intervention and post-intervention surveys conducted at 4-months intervals. Two times before, once during and one month after the intervention.  Entomological data in the form of Breteau indices and container-specific Breteau indices were calculated.  Outcome variables analysis conducted at the household and community levels.  **4) Insight-driven segmentation**: NA  **5) Co-creation**: - Community meetings and meetings with health committees were used to plan activities.  - Health committees organized and carried out clean-up campaigns and sewage maintenance work.  -Health committees created per suggestion of residents. |
| **Intervention ID:** I18-SCH  **Author, year:** Yuan et al., 2005  **Year of implementation:** 2000  **Location:** Junshan district, Hunan Province, China, Western Pacific,Upper middle income | **NTD:** Schistosomiasis  **Targeted stream:** Downstream  **Public:**  Fifth grade students in primary school.  **Setting:** 30 schools, 15 experimental/15 control.  **Sample size:**  Baseline/endpoint survey: 1137 pupils.  Treatment group: 604 (290 males, 314 females).  Control group: 527 (291 males, 236 females). | **Aim:** Increase children's knowledge and adherence to screening and chemotherapy treatment.  **Behaviors:** Increase adherence to screening and chemotherapy treatment.  **Description:** 2-month intervention.  Training program for anti-schistosomiasis station staff and teachers in experimental schools who would deliver the intervention.  Explanation of intervention purpose to parents and schoolchildren.  Display to schoolchildren and parents of a 15-min cartoon and film footage of schoolchildren discussions.  Distribution of a comic book.  Class discussion teacher-students, parents present.  A week after the intervention: school children designed poster to encourage classmates to adhere to screening and treatment.  **WHO NTD strategy:** Preventive chemotherapy and transmission control (PCT) | **Concepts:** **1) Social Behavioral influence:**  - Increase schistosomiasis screening (blood/stool samples) among schoolchildren and their parents.  **2) Public / people orientation:**  - Baseline survey provided information about children's primary media source for information and informed the development of a CD with messages related to signs, symptoms, diagnosis methods, drug treatment and places for examination.  - Experimental and control groups pre-tested.  - CD with multimedia material tested.  - Evaluation: pre-test and post-test  **3) Social offerings:** NA  **4) Relationship building:**  - At the initiation of the project efforts to draw attention to provincial and local health and education sectors about the problem (e.g. schistosome infection, poor compliance with screening/treatment).  - Intersectoral collaboration: Anti-schistosomiasis station staff (health sector) and teachers (education sector) involved in delivering the intervention. | **Techniques:** **1) Integrated intervention mix:**  - Training to local anti-schistosomiasis station staff and teachers to standardize the intervention delivery.  - CD with 15-min cartoon and film footage of discussions with schoolchildren were watched by schoolchildren and parents. Comic book using the cartoon characters complemented the CD. Cartoon and comic reinforced each other messages.  - Schoolchildren designed motivating posters.  - Parents participated in the CD screening and students-teacher discussions.  - The intervention was designed considering the provincial policy on health education for schistosomiasis  **2) Competition analysis and action:** NA  **3) Systematic planning and evaluation:**  - Evaluation methods: questionnaires, focus group discussions, behavioral observations.  - Databases from schistosomiasis screening conducted annually by the anti-schistosomiasis stations were used, with a focus on the years 1999 and 2000, to assess the behavioral effect of the intervention. In 1999 a total of 20 villages were included, 6 part of this study (3 experimental/3 control). In 2000 a total of 15 villages were screened, including the 6 villages part of the study. Blood examination was carried out eight months after the intervention.  **4) Insight-driven segmentation:**  - Based on local circumstances and to ensure usage, the cartoon and film were recorded in CD format because CD players are standard equipment in Chinese schools and some households have them  **5) Co-creation:**  - Students participated in designing posters to motivate behavior change among peers. |
| **Intervention ID:** I19-DEN  **Author, year:** Fernández et al., 1998  **Year of implementation:** 1996  **Location:** El Progreso, Honduras, Americas, lower middle income | **NTD:** Dengue  **Targeted stream:**  Midstream  **Public:** Community householders, focused on the people responsible for cleaning washbasins, usually the housewife.  **Setting:** 268 intervention households, 301 untreated households that were exposed to radio spots.  **Sample size:**  13 peri-urban neighborhoods (8 intervention, 5 untreated).  Total number of households in pre-intervention, 1st post-intervention and 2nd post-intervention surveys: n=268 intervention, n=301 untreated. | **Aim:** Promote the use of the cleaning method "La Untadita" to reduce mosquito infestation in cement washbasins and metal drums.  **Behaviors:** Implementation of cleaning method (La Untadita) for washbasins and metal drums.  **Description:**  5-steps cleaning method "La Untadita" (The Little Dab in English) was promoted in two intervention rounds with a 5-month interval in between.  Round 1: One week prior to the intervention banner announcements. Household visits by 248 volunteers and project staff to explain the method to householders. A sticker with the Untadita steps attached closed to the washbasin. Two weeks after, visits to 1784 houses to reinforce the method. Key chains and stickers were given as incentives if washbasins/drums were found without mosquito larvae.  Round 2: Modified Untadita method, face-to-face promotion at households, distribution of stickers, 5 promotion radio spots in 2 radio stations.  **WHO NTD strategy:**  Vector ecology and management | **Concepts:** **1) Social Behavioral influence:**  - Adopt and use the 5-steps Untadita method to clean washbasins.  Steps: mixing chlorine bleach and detergent to make a paste, applying the mixture to the walls of the washbasin/drum, waiting 10 minutes, scrubbing with a brush/sponge, and rinsing with water.  **2) Public / people orientation:**  - Previous experience of the research team in the setting informed the creation of the Untadita method for cleaning washbasins and drums, that was developed considering the practices and materials already used by householders for cleaning, availability (place) and affordability (price) of materials.  - The method and instructions were simplified in the second round of implementation.  **3) Social offerings:**  The Untadita cleaning method, using materials at the reach of the audience and not demanding drastic changes in behavior, was promoted.  **4) Relationship building:**  - Volunteers and project personnel conducted household visits to explain Untadita. | **Techniques:** **1) Integrated intervention mix:**  - Cleaning method "La Untadita"  - The cleaning method required common household materials easy to purchase and affordable; and was used in households  - One week before the start of the intervention, banner placement. The method was explained in the households by volunteers and project staff during household visits. Stickers near washbasins served as reminders. Key chains and stickers used as rewards. Radio announcements (spots, 10-min weekly program).  **2) Competition analysis and action:** NA  **3) Systematic planning and evaluation:**  - The Untadita method was developed in laboratory and field trials.  - Field trial with a pre-intervention and post-intervention surveys conducted after the first and second intervention rounds in intervention and untreated groups.  - Post-intervention surveys consisted on: (i) entomological evaluation for the presence of immature stages of Ae. aegypti and infestation intensity on washbasins and metal drums, (ii) knowledge, intervention exposure and reported behavior survey. In-depth interviews and observations of application of the method were conducted after the end of each survey.  - A variable, the Washbasin Infestation Index (WII), was created to summarize impact on immature stages of *Aedes* Aegypti.  **4) Insight-driven segmentation:**  - The cleaning method was created based on previous experience of the research team in the setting. To be sustainable, they decided to use materials already used by householders to clean washbasins and drums.  - The cleaning method was modified after the first round of intervention delivery due to limited impact on mosquito larvae and pupae. The instructions were simplified for the second round.  **5) Co-creation:** NA |
| **Intervention ID:** I20-SCH  **Author, year:** Yuan et al., 2000  **Year of implementation:** 1996  **Location:** Donting Lakes region, China, Western Pacific Region, upper middle-income country | **NTD: Schistosomiasis**  **Targeted stream:** Downstream.  **Public:** Fourth grade primary school students.  **Setting:** 50 primary schools located in the Donting Lakes region (north, east, south)  **Sample size:** Baseline survey: n=2263 school children from 50 schools;  Post-intervention survey: n=1739 school children from 48 schools; intervention group: 875 (438 males, 437 females), control group: 864 (485 males, 379 females). | **Aim:** Reduce contact with infested water by discouraging children from playing with it.  **Behaviors:** Reduce contact with unsafe water sources.  **Description:** 15-minute cartoon-style video and comic book with messages about transmission and prevention.  The video was screened twice on a same day to children in grade 4 in the intervention schools. The first screening was followed by a 10-minute class discussion. The comic book was distributed to the children who watched the video.  **WHO NTD strategy:** Water, sanitation and hygiene. | **Concepts:** **1) Social Behavioral influence:**  - Reduce contact with unsafe water.  - Observed water contact activities: play, swimming, washing hands and feet, and fishing to a less extent.  **2) Public / people orientation:**  - Baseline survey and observations informed the content of the video and comic book.  - Video and comic book pretested with students outside the study settings but in the same endemic region. The material was edited after the pretest.  **3) Social offerings:** NA  **4) Relationship building:** NA | **Techniques:** **1) Integrated intervention mix:**  - 15-minutes color video, video screening, discussions, color comic book. They reinforced each other.  - Took place in schools, delivered by anti-schistosomiasis station staff.  - Videos screened twice on one day. 10-minutes discussions.  **2) Competition analysis and action:** NA  **3) Systematic planning and evaluation:**  - Evaluation conducted.  - Evaluation design: Quasi-experimental study.  A month after the intervention: 10-day post-intervention observations.  Post-intervention survey (self-reported) on knowledge of schistosomiasis transmission and infection and on use of water (e.g. frequency, place).  **4) Insight-driven segmentation:**  - Educational material developed according to findings from baseline survey and edited after pre-testing. The video was screened twice responding to request from children during the pretesting.  **5) Co-creation:** NA |
