# Supplementary material for: Social marketing interventions for the prevention and control of neglected tropical diseases: A systematic review
Source: PLoS Negl Trop Dis. 2020 Jun 17;14(6):e0008360. doi: 10.1371/journal.pntd.0008360 (PMC7299328; doi:10.1371/journal.pntd.0008360)
Supplement: S3 File — (DOCX) [file pntd.0008360.s003.docx]

**S3 File. Intervention outcomes per priority strategy and disease.**

| **WHO Priority Strategy,**  **NTD, Author, year** | **Outcomes** | | |
| --- | --- | --- | --- |
|  | **Behavioral Determinants** | **Behavior Change** | **Health Outcomes** |
| ***Preventive chemotherapy and transmission control (PCT)*** | | | |
| *Lymphatic filariasis (LF)* | | | |
| Ramaiah et al., 2006 | *Knowledge:*  - Elephantiasis spread through mosquito bites: Pre-COMBI 34%, post-COMBI(+) 82%, post-COMBI(++) 73%.  - Believed the disease could be eliminated from their communities: Pre-COMBI 45%, post-COMBI(+) 51%, post-COMBI(++) 74%. | - Householders who had received the tablets: Pre-COMBI 71%, post-COMBI(+) 82%, post-COMBI(++) 88%.  - Householders who had ingested the tablets: Pre-COMBI 33%, post-COMBI(+) 37%, post-COMBI(++) 49%. | NA |
| King et al., 2011 | *Awareness:*  - Had heard of filariasis: Pre-MDA 2003 KAP survey (n=126, 82.4%); Post-MDA 2004 Survey (n=249, 95.4%). Increase (x2=19.2; p<0.001).  - Sources of information: Pre-MDA 2003 KAP survey: TV (39.8%), radio (34.5%), church (1.9%), village major (8.3%). Post-MDA 2004 Survey: TV (62.2%), radio (43%), newspaper (39%), church (27.3%).  *Knowledge:*  - 91.2% of respondents who had heard of filariasis knew what it was. Increase from the 2003 KAP survey (x^2^=20.1; *p*<0.001). | - Survey participants reporting taking tablets in the MDA-2002 (n=83, 54.3%), MDA-2004 (n=213, 81.6%).  - Coverage: MDA-2002 pre-modifications (49%). Post-modifications: MDA-2003 (71%), MDA-2004 (65%). The drug coverage survey reported a 86% coverage after the MDA-2004. Sustained increased coverage afterwards: MDA-2005 (67%), MDA-2006 (70%).  - Compliance MDA-2004: 86.4% (95%CI, 83.8-88.9%). | NA |
| Krentel et al., 2006 | *Awareness*  Had heard common LF Indonesian names:  - Filariasis: Men R1=26%, R2=91%; women R1=14%, R2=88%  - Kaki gajah (elephant leg): Men R1=43%, R2=94%; women R1=34%, R2=89%  - Boa besar (big fruit or ball): Men R1= 31% R2=84%; women R1=20%, R2=77%  *Attitudes*  Filariasis is a problem: R1=60%, R2=89%  *Knowledge*  - Knew filariasis is caused by worms in the blood R1=16% R2=67%  - Knew filariasis is transmitted by mosquitos R1=33% R2=77%  - Knew at least three symptoms for filariasis R1=37% R2=71%  - Knew that infection with filariasis can be prevented R1=57% R2=97%  *Policy:*  The intervention results supported the Indonesian Government in the policy change to accept the use of DEC and albendazole through MDA in endemic areas. | - Pre-MDA: Respondents that reported having had previously taken the treatment for filariasis= 21%. Post-MDA: Respondents who took the treatment during the intervention treatment period=88%.  - R2: 90% of the 321 individuals reporting compliance, experienced some adverse reaction to treatment, of these 75% sought side effect support from CHW. | NA |
| *Schistosomiasis* | | | |
| Yuan et al., 2005 | *Attitude towards adherence with screening and treatment for schistosomiasis:*  Baseline survey: No difference between experimental and control group.  Post-intervention survey: Differences found. Willing to provide stool sample: 96.4% experimental, 31.3% control; blood samples: 98% experimental, 32.1% control. Would take the drug: 98.2% experimental, 40% control.  *Knowledge*  Significant differences in the post-intervention survey between the experimental and control groups. The survey total scores were 100 points, 60 points was set as passing score; 99.8% of the experimental group passed, 16.7% of the control group passed. | - Case identification through blood examinations: Pre-intervention in 1999 (n=2318/15,900): 10-14 years (children) 13.4%, 30-49 years (parents) 29.4%. Post-intervention in 2000 (n=3270/12,500): 10-14 years (children) 18.7%, 30-49 years (parents) 33.4%.  - Children aged 10-14 years presenting for blood examination:  Year 1999 (n=318): 19.2% experimental village, 22% control village, 59.8% other village. Year 2000 (n=613) 38.2% experimental village, 22.4% control village, 39.4% other village. | NA |
| ***Innovative and intensified disease management (IDM)*** | | | |
| *Leprosy* | | | |
| Salgado, 1993; Williams et al., 1998; Wong, 2002; Brown, 2006 | *Awareness*  - Changed perception of leprosy by health care providers reflected in the recruitment of additional staff  *Structure*  - The national network of field clinics was increased from 75 to 225.  - Closer collaboration between the general health services and the anti-leprosy campaign.  - All the preventive health staff was trained to detect and treat the disease.  - Individual costs of transportation and lost wages were reduced due to the improved the network of clinics. | - Within 6 months of the campaign: 150% increase in new patients, of whom 40% were self-reporting. This number increased by 50% within a year.  - Leprosy was detected and treated for 16.700 sufferers since 1990, over 8.700 more than the pre-intervention average of about 1.000 cases per year. | - National prevalence rate for leprosy in 1989 before the start of the intervention was of 1.4 per 10.000 inhabitants, it increased to 1.6 in 1990 and decreased to 0.9 per 10.000 inhabitants in 1996.  - Sri Lanka achieved the WHO leprosy elimination target of less than one case in 10.000 by 1996. |
| ***Vector ecology and management (VEM)*** | | | |
| *Dengue* | | | |
| Caprara et al., 2015; Alfonso-Sierra et al., 2016 | *Knowledge:*  - Increase of people's knowledge of dengue and intention to participate in preventive actions.  *Policy:*  - Study findings motivated decision by the national government to implement the eco-health approach along with a monitoring program to measure the impact of dengue incidence and the costs for the program. | - Reduction in the dengue vector population.  - House index (%): Pre-intervention (dry season): control (0.8383), intervention (1.2944); post-intervention (rainy season): control (3.1664), intervention (2.0497); *p*-value (0.029).  - Container index: Pre-intervention (dry season): control (0.1625), intervention (0.1799); post-intervention (rainy season): control (0.7157), intervention (0.2228); *p*-value (0.020).  - Breteau index: Pre-intervention (dry season): control (1.0278), intervention (1.5991); post-intervention (rainy season): control (4.3158), intervention (2.4646); *p*-value (0.014).  - Pupae per person index: Pre-intervention (dry season): control (0.0104), intervention (0.0229); post-intervention (rainy season): control (0.0539), intervention (0.0292); *p*-value (0.023). | NA |
| Escudero-Támara and Villareal-Amaris, 2015 | B*eliefs:*  Inadequate: before 11 (20.4%), after 3 (5.6%); adequate: before 43 (79.6%), after 51 (94.4%)  *Knowledge:*  Inadequate: before 8 (14.8%), after 2 (3.7%); adequate: before 46 (85.2%), after 52 (96.3%) | *Practices:*  Inadequate: before (n=41, 76%), after (n=7, 13%); adequate: before (n=13, 24%), after (n=47, 87%)  *Presence of breeding places:* Absence: before (n=4, 7.4%), after (n=35, 64.8%). Presence: before (n=50, 92.6%), after (n=19, 35.2%).  *Types of breeding places:*  - Tanks with drinking water without lids: before (n=49, 48%), after (n=22, 46%).  - Trash cans without lids: before (n=18, 17%), after (n=10, 21%).  - Bottles and containers scattered around the patio: before (n=31, 30%), after (n=12, 25%).  - Tires with water: before (n=5, 5%), after (n=4, 8%).  *Change process:*  Stage 6 - Action: before 25.9% of participants, after 64.8% of participants.  Stage 7 - Maintenance: before 16.7% of participants, after 18.5% of participants. | NA |
| Abeyewickreme et al., 2012; Arunachalam et al., 2010 | *Structural*  - Improved garbage collection services.  - Collaboration between the volunteers and the local government authorities established.  - Communities surrounding the intervention clusters also benefited from have more frequent and more organized garbage collection. | - Number of *Aedes* larvae and pupae per household in the intervention and the control clusters were reduced.  - No significant differences were found on pupal and larval indices of *Aedes* mosquitos between the intervention and control clusters.  - Significant reduction in the number of pupae per 100 persons PPP at the end of the study between the intervention and control groups. | NA |
| NK Ibrahim et al., 2009 | *Knowledge*  - Poor score: pre-test 42.3% (n=1338), post-test 0.2% (n=6); fair/satisfactory score: pre-test 57.7% (n=1826), post-test 99.8% (n=2807).  *Attitudes*  Percentage of participants agreeing about the importance of:  - Dengue fever as a health problem in Jeddah: Pre-test 83.8%, post-test 95.7%.  - Making mosquito searchers at home: Pre-test 86.3%, post-test 96.1%.  - Spraying insecticides at home: Pre-test 88.8%, post-test 97.1%.  - Using mosquito repellents: Pre-test 79.9%, post-test 97.9%.  - Installing windows nets: Pre-test 80.2%, post-test 95.1%.  - Removing or covering stagnant water collection: Pre-test 92.2%, post-test 99.5%. | *Practices*  - Poor score: pre-test 32.6% (n=1031), post-test 4.6% (n=130); fair/satisfactory: pre-test 67.4% (n=2133), post-test 95.4% (n=2683). | NA |
| Lloyd et al., 1992; Winch et al., 1991; Lloyd et al., 1994; Kendall et al., 1991 | Knowledge:  - Changes observed in the intervention group in relation to larval production sites outside the home (23% stated tires, *P* < 0.02) and inside the home (11% stated vases, *P* < 0.0003).  - Effective methods for mosquito control: significant increases in the number of respondents mentioning cleanliness (*P* < 0.02), all patios should be clean (*P* < 0.05). | - Breteau index: intervention group baseline (126), 6-month post-intervention (129); control group baseline (113.4), 6-month post-intervention (151.3).  - Mean number of containers positive to *Ae. Aegypti* larvae per house lot decreased significantly in the intervention group (from 1.5 to 1.2, by paired *t-test*; *P* < 0.05). In the comparison group it increased significantly (from 1.2 to 1.6, by paired *t-test*; *P* < 0.03). | NA |
| Leontsini et al., 1993; Kendall et al., 1991 | *Knowledge:*  Post-intervention: relative increase of 57% for the intervention group compared to the control group and the pre-intervention levels (79% vs. 58% respectively, χ^2^ test, P<0.01). Comparing the intervention group with the control group, a significant increase was observed in the proportion of respondents who could mention (freely) 3 of the 4 control measures promoted. | - *Impact on larval densities:* *Ae. Aegypti* Breteu index: Pre-intervention, no significant difference found between intervention and control groups. Post-intervention, in the intervention group the number of positive containers was virtually not changed, in the control group a considerable increase was observed.  - *Breteau index:* Relative reductions in 3 intervention neighborhoods were observed. The other intervention neighborhood (number 3) had a relative increase.  - *Ae. aegypti* house index*:* Relative reduction in 3 intervention neighborhoods, relative increase in one (number 3).  - *Intervention effect on the 4 types of containers:* Reduced infestation in drums. No absolute reduction was observed in the other 3 types of containers (tyres, pilas, cans).  - *Culex* house index: relative reductions were observed in the intervention neighborhoods when compared with the untreated pair and the pre-intervention level. | NA |
| Fernández et al., 1998 | *Awareness:*  - Had heard of the Untadita: 1st post-intervention survey: 93.6% intervention, 10% untreated; 2nd post-intervention survey: 97.2% intervention, 42.8% untreated.  - Recalled hearing about Untadita from radio: 1st post-intervention survey: 44.4% intervention, 1.1% untreated; 2nd post-intervention survey: 32.9% intervention, 14.3% untreated.  *Knowledge:*  - Untadita steps mentioned (mean ± S.D.) 1st post-intervention survey: 3.24 ± 2.10 intervention, 1.04 ± 1.89 untreated; 2nd post-intervention survey: 4.4.± 1.15 intervention, 1.61 ± 2.07 untreated. (*t*-*test*, difference in number steps mentioned by intervention group at the two time points, *t* = -7.6, *p*<0.001).  - Recited steps in correct order: 1st post-intervention survey: 39.3% intervention, 14.8% untreated; 2nd post-intervention survey: 62.4% intervention, 17.8% untreated. | - Changes in human behavior were achieved but the level of reduction was not enough for adequate control of mosquito.  - Mean overall infestation index (WII) for washbasins: Pre-intervention: 0.59 ± 1.11 intervention, 0.72 ± 1.24 untreated, *t* = 1.299, n.s.; 1st post-intervention: 0.68 ± 1.22 intervention, 0.79 ± 1.33 untreated, *t* = 1.00, n.s.; 2nd post-intervention: 0.40 ± 0.91 intervention, 0.62 ± 1.22 untreated, *t* = 2.23, *p* = 0.026. | NA |
| ***Water, sanitation and hygiene (WASH)*** | | | |
| *Cysticercosis* | | | |
| Dickey et al., 2015; Dickey et al., 2016; Dickey, 2014 | *Knowledge:*  - Villagers increased knowledge and ability to build three-chamber toilets meeting sanitary criteria. Knowledge transfer to a neighboring village.  *Structural:*  - Households dissatisfied with smell: intervention (Q=0), comparison (Q=47)  - Households dissatisfied with convenience: intervention (Q=0), comparison (Q=43)  - Households dissatisfied with price: intervention (Q=0), comparison (Q=12) | - Households with members of the family refusing to use new toilet: intervention (Q=0), comparison (Q=36) corresponding to 42% of the toilets built by outside experts.  - Household toilets built in the intervention villages: before the intervention Q=4 in 2010, Q=1 in 2011. At the end of the intervention 70 toilets built.  - Toilets built in the comparison villages ("outside expert" program): 98 (85 built by outside experts).  - Other toilets built by or with the support from local builders of the intervention village: build in a school (Q=1), in a senior citizens center (Q=1), and in a neighboring village (Q=15). | NA |
| *Guinea-worm disease (Dracunliasis)* | | | |
| Brieger et al., 1989; Brieger et al., 1986; Brieger et al., 1990; Adeniyi and Brieger, 1983 | *Knowledge:*  - On first visit after purchase users remembered the steps for the correct use of the filters.  - Filter buyers remembered 7.6 out of the 10 items required for the safe and correct use the filter.  *Structural:*  - Total filter sales exceeded $700 USD, the amount was donated to the PHW Association to dig 2 community wells post-intervention. | *Monthly monitoring*  - 32.6% (n=254) of monitored households purchased a filter.  - Water filter users reported their use for: drinking (1st common use), cooking (mentioned in average by 40% of users per month) and washing dishes (7% average/month).  *Follow-up survey:*  - Out of 65 households from the town northeast (2nd sector), 54 were still using the filters.  - In average, the reported weekly use of the filter was of 4.2 times.  - Only 5 of the current filter users would be interested in buying a new filter. | NA |
| Adeyanju, 1987 | *Knowledge*  - Changes in knowledge among heads of household in the experimental villages.  - Knowledge of effective preventive measures in the experimental villages: Pre-intervention: 7% (n=200) heads of household. Post-intervention 68% (n=NA).  *Structural*  - 4 experimental villages completed sanitary wells.  - 2 experimental villages had begun well construction.  - 1 experimental village was considering plans for wells.  - No plans by the control villages to build wells. | - Behavior change among the villagers in relation to guinea-worm disease preventive actions.  Observed changes in behavior: use of cloth water filters, carrying clean water along for work in the farms, discouragement from entering water sources if having visible blisters, placement of stones at the edge of water supplies to avoid water contact.  - Case referrals to the health center by village health volunteers. | NA |
| *Schistosomiasis* | | | |
| Yuan et al., 2000 | *Knowledge*  - Experimental group more knowledgeable about schistosomiasis than the control group. | - *Self-reported frequency of water contact:* Statistically significant difference by frequency of water contact activities between the intervention and control groups (χ^2^=3.88, *P*<0.1).  - *Self-reported place of water contact:*  Intervention group: Decreased use of unsafe water, decreased used of safe/unsafe water, increased use of safe waters only.  Control group: Increased use of unsafe water as the only or as one of many swimming areas, decreased use of safe water sites.  - *Observed types of water contact activity:*  Pre-intervention no statistically difference found between experimental and control group.  Post-intervention statistically significant differences were found in all forms of water contact behavior (swimming, playing, hand wash, feet wash, fishing). | NA |
| *Soil-transmitted helminths* | | | |
| Bieri, Yuan, et al., 2013; Bieri, Gray, et al., 2013 | *Knowledge:*  - Follow-up: Knowledge of helminths was 90% higher in the intervention than in the control group (63.3 vs 33.4, *P*<0.001). | - Intervention associated with 50% efficacy (95% CI, 30 to 65) in preventing infection with STHs.  - Children who washed their hands after using the toilet: intervention group: 98.9%, control group: 54.2%: *P*<0.001. | - Incidence of STH infection: intervention: 4.1% (95% CI, 2.8 to 5.5); control: 8.4% (95% CI, 6.6 to 10.2); *P*<0.001.  - Intervention associated with 50% efficacy (95% CI, 30 to 65) in preventing infection.  - Follow-up incidence of infection higher in boys than among girls (*P*=0.002). |
| *Trachoma* | | | |
| Atkinson et al., 2014; Lange et al., 2014; Lange et al., 2017; Baunach et al., 2012; Stanford et al., 2016; Lange et al., 2016; Taylor et al., 2012; Lange et al., 2012; Lange et al., 2015; Jones et al., 2015**;** Lange, JR Atkinson, et al., 2013; Lange, J Atkinson, et al., 2013 | *Awareness*  69.2% of participants had seen/heard at least one of the health promotion initiatives  *Attitudes*  - Significant decrease in acceptance of dirty faces as "normal" (40.5% *cf* 29.6%; χ^2^ *p*=0.009)  *Knowledge/Skills*  - Significant increase in ability to teach others about trachoma prevention (70.8% *cf* 83.3%; χ^2^ *p*<0.001) | - In clinics: Marginal increase in respondents being able to screen for active trachoma (70.3% *cf* 75.4%; χ^2^ *p*=0.32)  - In schools: Significant increase in ability to teach others about trachoma prevention (50.0% *cf* 73.1%; χ^2^ *p*=0.04)  - In community workplace: Increase in ability to teach others about trachoma prevention (60.0% *cf* 79.2%; χ^2^ *p*=0.04) | Prevalence of trachoma in children aged 5-9 in screened communities in the Northern Territory: 2009 = 15%, 2010 = 15%, 2011 = 7%, 2012 = 4%, 2013=5%, 2014 = 5.9%, 2015 = 4.8%  Prevalence of clean faces in children aged 5-9 in screened communities in the Northern Territory: 2009 = 76%, 2010 = 74%, 2011 = 74%, 2012 = 75%, 2013 = 78%, 2014 = 86%, 2015 = 85% |
| ***Combined: PCT and WASH*** | | | |
| *Schistosomiasis* | | | |
| Freudenthal et al., 2006 | *Structural*  - Teachers initiated their own actions to develop a curriculum to improve schistosomiasis education in primary schools this included incorporating household sanitation surveys in school activities.  - Community members started to create safe swimming places. | NA | NA |
| Hu et al., 2005 | *Attitude about chemotherapy (% passed):*  - Schoolchildren: Experimental pre-intervention (55.1%), post-intervention (98.9%). Control pre-intervention (51.9%), post-intervention (49.4%).  - Women: Experimental pre-intervention (32%), post-intervention (91.9%). Control pre-intervention (31.7%), post-intervention (28.2%).  - Men: Experimental pre-intervention (67.1%), post-intervention (97.5%). Control pre-intervention (72.6%), post-intervention (74.3%).  *Knowledge of schistosomiasis (% passed):*  - Schoolchildren: Experimental pre-intervention (9%), post-intervention (94.4%). Control pre-intervention (7.4%), post-intervention (8.6%).  - Women: Experimental pre-intervention (55.3%), post-intervention (84.8%). Control pre-intervention (56.4%), post-intervention (57.3%).  - Men: Experimental pre-intervention (80%), post-intervention (91.4%). Control pre-intervention (78.6%), post-intervention (78.9%). | *- Post-intervention, experimental villages:* Decrease in the frequency of water contact of schoolchildren (to 0.6%) and women (to 5.6%). Increased in men (to 36.9%).  - 5 years post-intervention: Frequency of infested water contact remained low among schoolchildren 0.6% (17/3040) and women 5.9% (9/152) and high among men 35.4% (63/178).  *Compliance with chemotherapy:*  - Schoolchildren: experimental pre-intervention (68.5%), post-intervention (98.9%); control pre-intervention (70.4%), post-intervention (61.7%).  - Women: experimental pre-intervention (52.4%), post-intervention (92.9%); control pre-intervention (60.4%), post-intervention (50.5%).  - Men: experimental pre-intervention (75.3%), post-intervention (92.1%); control pre-intervention (81.6%), post-intervention (69.6%).  - Chemotherapy compliance increased by: (44.4%) schoolchildren, (77.3%) women, (22.3%) men. | *Experimental villages:*  - Prevalence of S. japonicum reduced by: (83.7%) schoolchildren and (63.4%) women. Slight decrease in men's rate of infection but not statistically significant (*P*>0.05).  *Control villages:*  Re-infection rates of S. japonicum similar throughout the study. |
