# Supplementary material for: Social marketing interventions for the prevention and control of neglected tropical diseases: A systematic review
Source: PLoS Negl Trop Dis. 2020 Jun 17;14(6):e0008360. doi: 10.1371/journal.pntd.0008360 (PMC7299328; doi:10.1371/journal.pntd.0008360)
Supplement: S4 File — (DOCX) [file pntd.0008360.s004.docx]

## S4 File. Results of the quality assessment per intervention.

| **Criteria** | **Brieger et al., 1989; Brieger et al., 1986; Brieger et al., 1990; Adeniyi and Brieger, 1983** | **Dickey et al., 2015; Dickey et al., 2016; Dickey, 2014** | **Caprara et al., 2015; Alfonso-Sierra et al., 2016** | **Ramaiah et al., 2006Ramaiah et al., 2006** | **Atkinson et al., 2014; Lange et al., 2014; Lange et al., 2017; Baunach et al., 2012; Stanford et al., 2016; Lange et al., 2016; Taylor et al., 2012; Lange et al., 2012; Lange et al., 2015; Jones et al., 2015; Lange, JR Atkinson, et al., 2013; Lange, J Atkinson, et al., 2013** |
| --- | --- | --- | --- | --- | --- |
| Q1. Explicit theoretical framework | 3 | 3 | 1 | 2 | 3 |
| Q2. Statement of aims/objectives in main body of report | 2 | 2 | 2 | 2 | 3 |
| Q3. Clear description of research setting | 3 | 3 | 3 | 3 | 3 |
| Q4. Evidence of sample size considered in terms of analysis | 0 | 1 | 1 | 1 | 1 |
| Q5. Representative sample of target group of a reasonable size | 2 | 1 | 3 | 3 | 3 |
| Q6. Description of procedure for data collection | 2 | 3 | 3 | 2 | 2 |
| Q7. Rationale for choice of data collection tool(s) | 2 | 3 | 2 | 3 | 2 |
| Q8. Detailed recruitment data | 0 | 1 | 2 | 2 | 1 |
| Q9. Statistical assessment of reliability and validity of measurement tool(s) (Quantitative only) | 0 | 0 | 0 | 0 | 1 |
| Q10. Fit between stated research question and method of data collection (Quantitative only) | 2 | 1 | 2 | 2 | 2 |
| Q11. Fit between stated research question and format and content of data collection tool e.g. interview schedule (Qualitative only) | 2 | 3 | 2 | 2 | na |
| Q12. Fit between research question and method of analysis | 2 | 2 | 2 | 2 | 2 |
| Q13. Good justification for analytical method selected | 2 | 2 | 0 | 1 | 2 |
| Q14. Assessment of reliability of analytical process (Qualitative only) | 0 | 2 | 0 | 0 | na |
| Q15. Evidence of user involvement in design | 3 | 3 | 3 | 2 | 2 |
| Q16. Strengths and limitations critically discussed | 2 | 1 | 2 | 1 | 2 |
| Score total | 27 | 31 | 28 | 28 | 29 |
| **Percentage of the maximum quality score obtained = (Score / maximum quality score) × 100** | **56,3%** | **64,6%** | **58,3%** | **58,3%** | **69,0%** |
|  |  |  |  |  |  |
| **Criteria** | **King et al., 2011** | **Krentel et al., 2006** | **Salgado, 1993; Williams et al., 1998; Wong, 2002; Brown, 2006** | **Freudenthal et al., 2006** | **Bieri, Yuan, et al., 2013; Bieri, Gray, et al., 2013** |
| Q1. Explicit theoretical framework | 2 | 2 | 2 | 3 | 3 |
| Q2. Statement of aims/objectives in main body of report | 2 | 3 | 1 | 3 | 3 |
| Q3. Clear description of research setting | 2 | 2 | 2 | 3 | 3 |
| Q4. Evidence of sample size considered in terms of analysis | 2 | 3 | 0 | 1 | 3 |
| Q5. Representative sample of target group of a reasonable size | 3 | 3 | 0 | 1 | 3 |
| Q6. Description of procedure for data collection | 2 | 3 | 1 | 3 | 3 |
| Q7. Rationale for choice of data collection tool(s) | 2 | 3 | 1 | 3 | 3 |
| Q8. Detailed recruitment data | 2 | 3 | 0 | 0 | 3 |
| Q9. Statistical assessment of reliability and validity of measurement tool(s) (Quantitative only) | 0 | 0 | 0 | na | 2 |
| Q10. Fit between stated research question and method of data collection (Quantitative only) | 3 | 2 | 0 | na | 3 |
| Q11. Fit between stated research question and format and content of data collection tool e.g. interview schedule (Qualitative only) | 3 | 3 | 1 | 3 | 3 |
| Q12. Fit between research question and method of analysis | 2 | 2 | 1 | 3 | 3 |
| Q13. Good justification for analytical method selected | 0 | 2 | 1 | 2 | 3 |
| Q14. Assessment of reliability of analytical process (Qualitative only) | 0 | 1 | 0 | 0 | 3 |
| Q15. Evidence of user involvement in design | 3 | 3 | 0 | 3 | 3 |
| Q16. Strengths and limitations critically discussed | 3 | 1 | 0 | 2 | 3 |
| Score total | 31 | 36 | 10 | 30 | 47 |
| **Percentage of the maximum quality score obtained = (Score / maximum quality score) × 100** | **64,6%** | **75,0%** | **20,8%** | **71,4%** | **97,9%** |
|  |  |  |  |  |  |
| **Criteria** | **Escudero-Támara and Villareal-Amaris, 2015** | **Abeyewickreme et al., 2012; Arunachalam et al., 2010** | **NK Ibrahim et al., 2009** | **Hu et al., 2005** | **Adeyanju, 1987** |
| Q1. Explicit theoretical framework | 1 | 2 | 2 | 2 | 3 |
| Q2. Statement of aims/objectives in main body of report | 3 | 2 | 3 | 3 | 3 |
| Q3. Clear description of research setting | 3 | 3 | 3 | 2 | 3 |
| Q4. Evidence of sample size considered in terms of analysis | 3 | 3 | 3 | 0 | 1 |
| Q5. Representative sample of target group of a reasonable size | 2 | 3 | 3 | 1 | 1 |
| Q6. Description of procedure for data collection | 2 | 3 | 2 | 3 | 3 |
| Q7. Rationale for choice of data collection tool(s) | 2 | 3 | 3 | 2 | 3 |
| Q8. Detailed recruitment data | 1 | 1 | 2 | 1 | 1 |
| Q9. Statistical assessment of reliability and validity of measurement tool(s) (Quantitative only) | 0 | 1 | 0 | 1 | 0 |
| Q10. Fit between stated research question and method of data collection (Quantitative only) | 2 | 3 | 2 | 2 | 1 |
| Q11. Fit between stated research question and format and content of data collection tool e.g. interview schedule (Qualitative only) | 2 | 2 | na | na | 3 |
| Q12. Fit between research question and method of analysis | 2 | 3 | 3 | 3 | 1 |
| Q13. Good justification for analytical method selected | 1 | 2 | 2 | 1 | 0 |
| Q14. Assessment of reliability of analytical process (Qualitative only) | 0 | 1 | na | na | 0 |
| Q15. Evidence of user involvement in design | 0 | 2 | 1 | 1 | 3 |
| Q16. Strengths and limitations critically discussed | 1 | 1 | 1 | 1 | 3 |
| Score total | 25 | 35 | 30 | 23 | 29 |
| **Percentage of the maximum quality score obtained = (Score / maximum quality score) × 100** | **52,1%** | **72,9%** | **71,4%** | **54,8%** | **60,4%** |
|  |  |  |  |  |  |
| **Criteria** | **Lloyd et al., 1992; Winch et al., 1991; Lloyd et al., 1994; Kendall et al., 1991** | **Leontsini et al., 1993; Kendall et al., 1991** | **Yuan et al., 2005** | **Fernández et al., 1998** | **Yuan et al., 2000** |
| Q1. Explicit theoretical framework | 3 | 2 | 3 | 3 | 2 |
| Q2. Statement of aims/objectives in main body of report | 3 | 3 | 2 | 2 | 2 |
| Q3. Clear description of research setting | 3 | 2 | 2 | 2 | 3 |
| Q4. Evidence of sample size considered in terms of analysis | 1 | 0 | 1 | 0 | 2 |
| Q5. Representative sample of target group of a reasonable size | 3 | 2 | 1 | 1 | 1 |
| Q6. Description of procedure for data collection | 3 | 3 | 2 | 2 | 1 |
| Q7. Rationale for choice of data collection tool(s) | 2 | 2 | 2 | 2 | 2 |
| Q8. Detailed recruitment data | 1 | 1 | 2 | 1 | 1 |
| Q9. Statistical assessment of reliability and validity of measurement tool(s) (Quantitative only) | 1 | 3 | 1 | 3 | 0 |
| Q10. Fit between stated research question and method of data collection (Quantitative only) | 3 | 2 | 2 | 2 | 1 |
| Q11. Fit between stated research question and format and content of data collection tool e.g. interview schedule (Qualitative only) | 3 | 2 | 1 | 3 | 3 |
| Q12. Fit between research question and method of analysis | 2 | 3 | 3 | 3 | 2 |
| Q13. Good justification for analytical method selected | 2 | 3 | 2 | 3 | 1 |
| Q14. Assessment of reliability of analytical process (Qualitative only) | 0 | 0 | 0 | 0 | 0 |
| Q15. Evidence of user involvement in design | 2 | 1 | 2 | 1 | 1 |
| Q16. Strengths and limitations critically discussed | 1 | 3 | 1 | 2 | 1 |
| Score total | 33 | 32 | 27 | 30 | 23 |
| **Percentage of the maximum quality score obtained = (Score / maximum quality score) × 100** | **68,8%** | **66,7%** | **56,3%** | **62,5%** | **47,9%** |
|  |  |  |  |  |  |
| **Average all interventions** | **62,5%** |  |  |  |  |
