# Supplementary material for: Social marketing interventions for the prevention and control of neglected tropical diseases: A systematic review
Source: PLoS Negl Trop Dis. 2020 Jun 17;14(6):e0008360. doi: 10.1371/journal.pntd.0008360 (PMC7299328; doi:10.1371/journal.pntd.0008360)
Supplement: S5 File — (DOCX) [file pntd.0008360.s005.docx]

**S5 File. List of Abbreviations**

AF Africa

AM Americas

DALYs disability-adjusted life-years

DEC diethylcarbamazine

DF dengue fever

EM Eastern Mediterranean

HIC high-income economies

IDM innovative and intensified disease management

KAP knowledge, attitudes, and practices

KBP knowledge, beliefs, and practices

LIC low-income economies

LMIC lower-middle-income economies

MDA mass drug administration

MFC Melbourne Football Club

NTDs neglected tropical diseases

PCT preventive chemotherapy and transmission control

PHWs primary health workers

PRISMA preferred reporting items for systematic review and meta-analysis

PRISMA-P preferred reporting items for systematic review and meta-analysis protocols

SEA South-East Asia

UMIC upper-middle-income economies

VEM vector ecology and management

WASH water, sanitation, and hygiene

WHO World Health Organization

WP Western Pacific
